# A Bibliometric Analysis of Microalgae Research in the World, Europe, and the European Atlantic Area

**DOI:** 10.3390/md18020079

**Published:** 2020-01-26

**Authors:** Judith Rumin, Elodie Nicolau, Raimundo Gonçalves de Oliveira Junior, Claudio Fuentes-Grünewald, Kevin J. Flynn, Laurent Picot

**Affiliations:** 1 La Rochelle Université, UMRi CNRS 7266 LIENSs, Avenue Crépeau, 17042 La Rochelle, France; judith.rumin@univ-lr.fr (J.R.); rgonca01@univ-lr.fr (R.G.d.O.J.); 2 IFREMER, Laboratoire BRM/PBA, Rue de l’Ile d’Yeu, 44311 Nantes, France; elodie.nicolau@ifremer.fr; 3 Department of Biosciences, Swansea University, Singleton Park, Swansea, Wales SA2 8PP, UK; c.fuentesgrunewald@swansea.ac.uk (C.F.-G.); k.j.flynn@swansea.ac.uk (K.J.F.)

**Keywords:** biotechnology, European Atlantic Area, microalgae, phytoplankton, research, market, bibliometrics

## Abstract

A bibliographic database of scientific papers published by authors affiliated worldwide, especially focused in Europe and in the European Atlantic Area, and containing the keywords “microalga(e)” or “phytoplankton” was built. A corpus of 79,020 publications was obtained and analyzed using the Orbit Intellixir software to highlight the evolution of the research domain. Publication rates from 1960 to 2019, organization of the research, collaboration networks between countries and organizations, emerging and fading research concepts, major studied species, and associated concepts, as well as journals publishing microalgae research were considered. As a result, of the 79,020 papers published worldwide, 26,137 included authors from Europe (33% of world production) and 6989 from the European Atlantic Area (AA) (27% of European production, 9% of world production). The main worldwide scientific research topics found in this study were *phytoplankton*, *community*, *bloom*, *diatoms*, *distribution*, *ecosystem*, *coastal*, *chlorophyll*, *zooplankton*, *photosynthesis*, and *primary* *production*. At the European scale, the most studied topics were related to the *environment*, *food*, *chemicals*, *pigments*, *protein*, *feed*, and *drugs*. The highest scientific trends and market opportunities analysis identified *bioplastics* and *biostimulants* as top emerging concepts at the European level and *agricultural*, *animal* *feed*, and *blue* *biotechnology* at the European AA level.

## 1. Introduction 

Microalgae, with an estimated number of 30,000 to 1,000,000 species, constitute a vast group of microorganisms extensively studied for their ecological functions in marine and freshwater environments and possible use as a source of feed, food, cosmetics, biofuels, nanomaterials, and pharmaceuticals [1]. Active research on microalgae started in western countries in the early 1950s, when growth systems allowing their production at the lab and industrial scale were first developed [2,3]. Since these pioneering works, research has progressively increased, diversified, and spread all over the world. The current research effort is mainly dedicated to isolate new strains, characterize the ecophysiology and metabolites of microalgae, model their productivity, control and improve the production of high added-value compounds, and develop sustainable and innovative applications. From the industrial and market perspective, microalgae production is identified as a business sector with high opportunities. Europe and the European Atlantic Area (AA) present a strong potential for research, innovation, and industrial development as they bring together a critical number of expert researchers, host biotech companies, and technological platforms working in international research networks and sustainably producing high-quality microalgae biomass. In this work, we present an in-depth review of microalgae research in a worldwide context, in Europe, and the European AA. Emerging research concepts were highlighted, from a perspective of regional bioeconomy development, differentiating from the global European and world activities.

## 2. Results

### 2.1. Overview of Microalgae Research in the World 

#### 2.1.1. Bibliometric Production 

**Figure 1:**
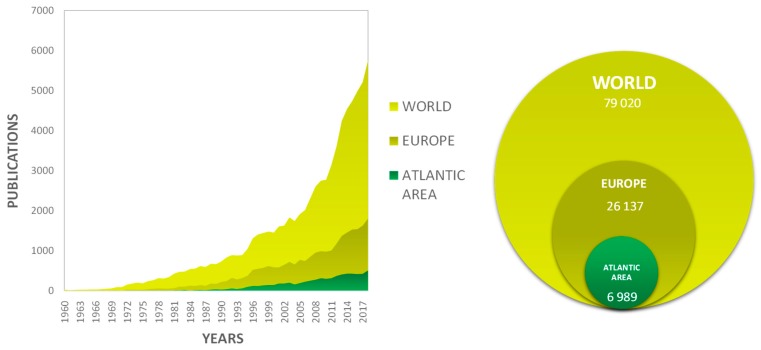
Evolution of the number of scientific papers published about microalgae and phytoplankton at the world, European, and European Atlatic Area (AA) level and contribution of the AA and Europe to world production.

Figure 1 presents the number of scientific papers published about microalga(e) and phytoplankton at the world, European, and European AA levels since the 1960s. A total of 79,020 papers were published, from which 26,137 included authors from Europe (33% of world production) and 6989 from the European AA (27% of the European production, 9% of the world production) (Table 1).

In 2018, the scientific production reached 5747 publications per year in the world, 1806 (31% of the world production) publications per year in Europe, and 509 (28% of the European production) publications per year in the AA. From the results explained above, it can be noted that Europe and the European AA follow roughly the same publication trend as the world trend. An increased interest of the scientific community in microalgae can be noticed since 2005, as demonstrated by the rapid increase in the world and European publication rates and the important inflection of the publication slope, as highlighted by Garrido-Cardenas et al. (2018). These authors estimated that a 15-fold acceleration of the publication rate was observed after 2005 [4]. In our data including environmental studies about microalgae and phytoplankton, this acceleration was lower, suggesting that the fast acceleration of publication rates may be mostly attributed to publications dealing with biotechnological applications of microalgae. This fast acceleration after 2005 could be due to the irruption of the use of microalgae as raw materia for the production of biodiesel [5]. Chisti’s paper [5] is one of the most cited papers in the microalgae field, with nearly 5000 citations so far. Concerning the scientific production of the European AA, a linear evolution of the number of publications can be observed from 1990 to 2018, with a slope coefficient of 0.96, suggesting that the scientific production does not follow the European and world publication production trends. This observation suggests that European countries and regions located outside the European AA contributed more to the European publication rate increase (Figure 1). Table 1 details the number of authors, affiliations, and concepts listed in the three databases. Europe occupies an important place in the world scientific production, as 33% of total publications were written by authors containing at least one European co-author. A total of 42% of the total world authors or co-authors were European, and these authors represented 54% of the world affiliations. In the same way, by comparing the scientific output of the AA with Europe, a large part of AA institutes/affiliations contributed to the European production of publications: 27% of the total European scientific publications were written by a list of authors containing at least one AA author or co-author, 37% of the total European authors or co-authors came from the AA, and these authors represented 69% of the European affiliations.

#### 2.1.2. International Collaborations with Europe and the European AA 

**Figure 2:**
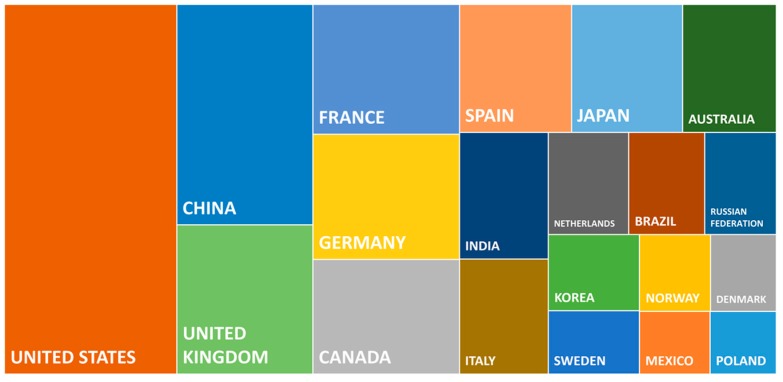
Top 20 countries publishing research about microalgae and phytoplankton. The square size represents graphically the number of publications by country.

Figure 2 presents the top 20 publishing countries in the field of microalgae and phytoplankton. The two main publishing countries are the United States (U.S.) and China, with 18,269 and 8601 publications, respectively. These results are in line with the data published by Garrido-Cardenas et al. (2018). The U.S. researchers are the most represented authors or co-authors in microalgae scientific publications worldwide. In total, 2368 publications involved the U.S. and European authors and 836 publications involved U.S. and European Atlantic authors. 

Concerning European countries, the United Kingdom, France, Germany, Spain, and Italy were the main European countries producing scientific publications in the field of microalgae worldwide, with 5818, 5448, 5248, 4106, and 2925 publications, respectively. The five countries of the European AA (Spain, Portugal, France, United Kingdom, and Ireland) were among the seven that published the most with co-authors in the AA. In this top seven, the U.S. and Germany published 836 and 494 scientific publications, respectively, with co-authors from the European AA. 

#### 2.1.3. Main Research Concepts

**Figure 3:**
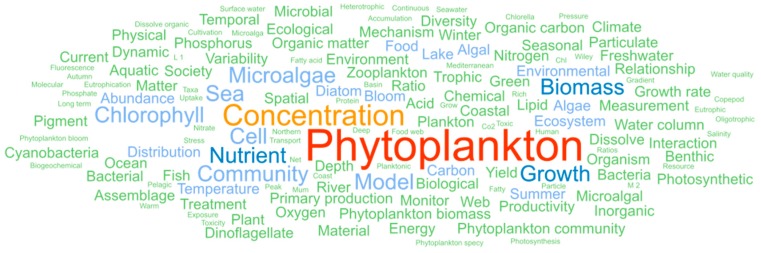
A total of 150 main common concepts of microalgae scientific publications in the world, the European, and the AA databases. The most prevalent concepts are identified in red and orange.

Figure 3 presents the main common concepts appearing in scientific publications at the world, European. and European AA levels. Overall, research concepts were the same at these three levels and correspond to environmental concepts such as *phytoplankton*, *community*, *bloom*, *diatoms*, *distribution*, *ecosystem*, *coastal*, *chlorophyll*, *zooplankton*, *photosynthesis*, and *primary production*. This environment/phytoplankton research domain can be differentiated from the microalgae/high-added-value molecules field that contained less and more recent publications, as previously reported [4]. 

#### 2.1.4. Emerging Research Concepts

Table 2 shows the list of emerging concepts that have shown the highest growth factor over the 2017–2019 period. These concepts represent scientific trends and opportunities at the world, European, and AA levels. *Feed* was the predominant emerging concept for the three geographical areas, with a very high growth factors (GF) compared to the rest of the emerging concepts (Table 2). Concepts in the field of biotechnology/high-added-value molecules were more prominent compared to concepts in the environmental/phytoplankton field. Interestingly, the emerging concepts of worldwide scientific publications covered the current societal and environmental issues of society, such as *organic*, *ecology*, *drugs*, *contamination*, *nutraceuticals*, *by-products*, and *genetic data*, as well as *green extraction techniques*. 

Table 2 also demonstrates a research interest for green and large scale treatment and harvest systems for microalgae, which represent a major challenge for the industrial development of microalgae through *mechanical pre-treatment* or *biosurfactant* emerging concepts appearing in publications worldwide. Another scientific emerging concept is related to *large-scale culture contamination*. Research on the genetic data of microalgae and metabolic pathways also emerged several years ago with concepts such as *Crispr/cas9*, *recombinant enzyme*, *transcription factor Nrf2*, *omics*, and *genome editing* in the world and European scientific publications. Furthermore, the table also highlights the emerging microalgae markets such as *bioplastics* and *biostimulants* in European scientific publications. Table 2 also shows some concepts specific to the AA that have emerged over the two last years and deal with the field of animal agriculture. These include new antibiotics and nutraceuticals for animals through the emerging concepts of *bovine*, *cattle*, and *veterinary medicine*. The interest of the European AA in the blue economy is also highlighted by the concepts of *circular economy* and *blue biotechnology*. The European AA is wellknown for its agricultural activities, and microalgae-based technologies will certainly play a key positive role in the near future as bioremediation environmental services tools related to these two concepts (*circular economy* and *blue technology*) [6].

### 2.2. Focus on the European Scientific Production 

The 28 countries of the European Union produced 26,137 publications in the field of microalgae and phytoplankton; these papers are analyzed in detail in this section.

#### 2.2.1. Collaboration Networks

**Figure 4:**
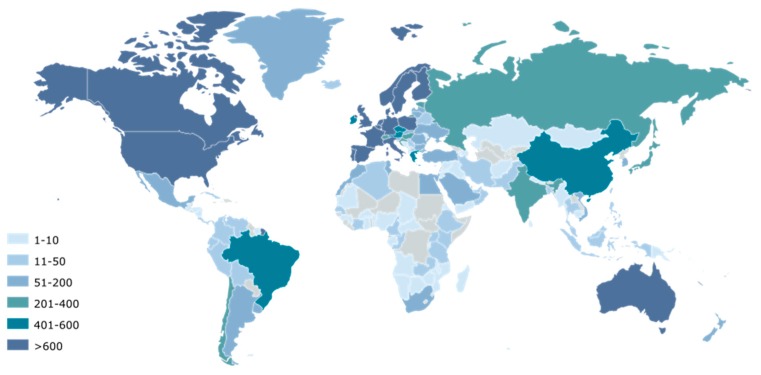
Main countries identified as scientific collaborators in the microalgae European publications. The color gradient indicates the number of joint publications of each country with the European union.

Figure 4 shows the network of worldwide collaborators by highlighting co-authorship of scientific publications with European authors in the field of microalgae and phytoplankton. The main collaborating countries of the European Union, with more than 600 joint scientific publications, are the U.S. (2668), Canada (877), Norway (735), and Australia (651). In addition, 400 to 600 scientific publications have been published in collaboration with China (562) and Brazil (452). Smaller collaborations leading to 200 to 400 joint publications with Europe have been listed for Russia (303), Japan (287), Chile (250), and India (203). Finally, collaborators having published between 50 and 200 scientific publications with Europe are Argentina (198), Mexico (198), New Zealand (186), Israel (161), South Africa (154), South Korea (118), Denmark (including Greenland) (105), Saudi Arabia (90), and Uruguay (67). 

Within the European Union, countries producing the largest number of publications are France (5445), Germany (5258), Spain (4100), Italy (2852), Netherlands (2353), the United Kingdom (2030), Sweden (1654), Denmark (1446), Poland (1192), Belgium (1189), Portugal (1164), and Finland (904). In addition, between 400 and 600 scientific publications have been published by Greece (556), the Czech Republic (555), Austria (484), and Ireland (416). The production of scientific publications was lower for Switzerland (348), Croatia (343), Hungary (338), and Estonia (319). 

**Figure 5:**
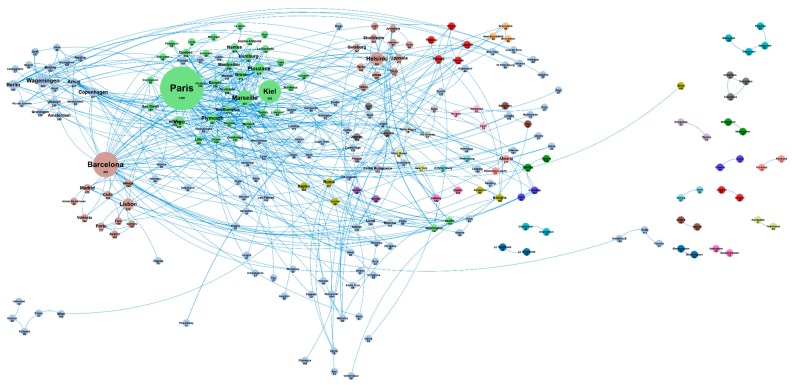
Main European cities and collaboration networks in the microalgae European publications (500 links; 9 co-occurrences; 10 occurrences; 24 clusters).

Figure 5 shows the collaboration networks existing between European cities involved in scientific publications produced in the European Union in the field of microalgae and phytoplankton. These networks highlight national and regional geographic clusters such as those of Wageningen, Lisboa, Helsinki, or Vigo. This figure also provides an overview of strong collaborations in Europe and highlights the lack of collaboration between some cities, suggesting potential opportunities for new collaborations.

#### 2.2.2. Scientific Production over the Years

Regarding the number of scientific papers published until 2019 for the 15 top publisher countries in Europe, France and Germany were the first to produce an important number of publications in Europe, with 100 publications per year since 1996 and 1997, respectively. Spain reached 100 publications per year in 2001, and the U.S. and Italy produced 100 per year in 2007. The number of scientific publications per country continues to increase each year. The city of Paris produced more than 50 scientific publications per year since 2007 and reached more than 100 publications per year since 2017. Wageningen, Lisbon, Kiel, and Barcelona each produced 50 scientific publications per year since the year 2000.

#### 2.2.3. Main Domains of Application 

**Figure 6:**
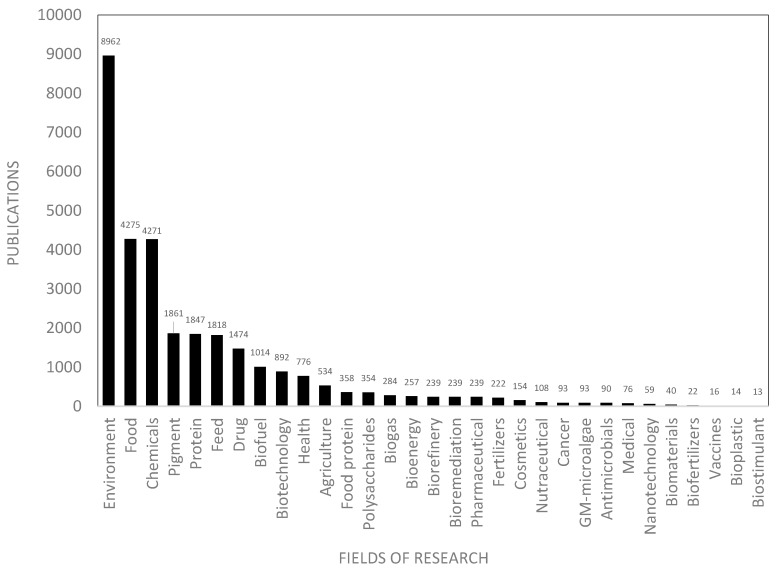
Main research domains identified in the microalgae scientific production in Europe.

Figure 6 shows the number of scientific publications by research area in Europe. The main research topic in Europe is the *environment*, with 8962 publications, dominating the domains related to *food* and *chemicals* that contain 4275 and 4271 publications, respectively. These keywords cover very broad topics and more specific markets have fewer publications (less than 2000 publications). For example, many publications have been produced in Europe in the field of *pigments* (1861), *proteins* (1847), *feed* (1818), *drug* (1474), *biofuel* (1014), and *biotechnologies* (892). Figure 6 shows the most recent niche markets such as *biostimulant* (13), *bioplastic* (14), *vaccines* (16), *biofertilizers* (22), and *nanotechnology* (59).

#### 2.2.4. Main Publishing Journals

Table 3 shows the main journals publishing European papers in the field of microalgae. *Hydrobiologia* was the top publisher in Europe, with 1162 publications and an impact factor of 2.165. The majority of these journals relate to environmental/phytoplankton research and have impact factors ranging from 1.897 to 4.61 for an average of 2.69. The main journals related to the microalgae/high-added value molecules field are *Bioresource Technology* and *Algal Research*, with impact factors of 5.807 and 3.745, respectively.

#### 2.2.5. Top 15 Studied Microalgae Genus in Europe

**Figure 7:**
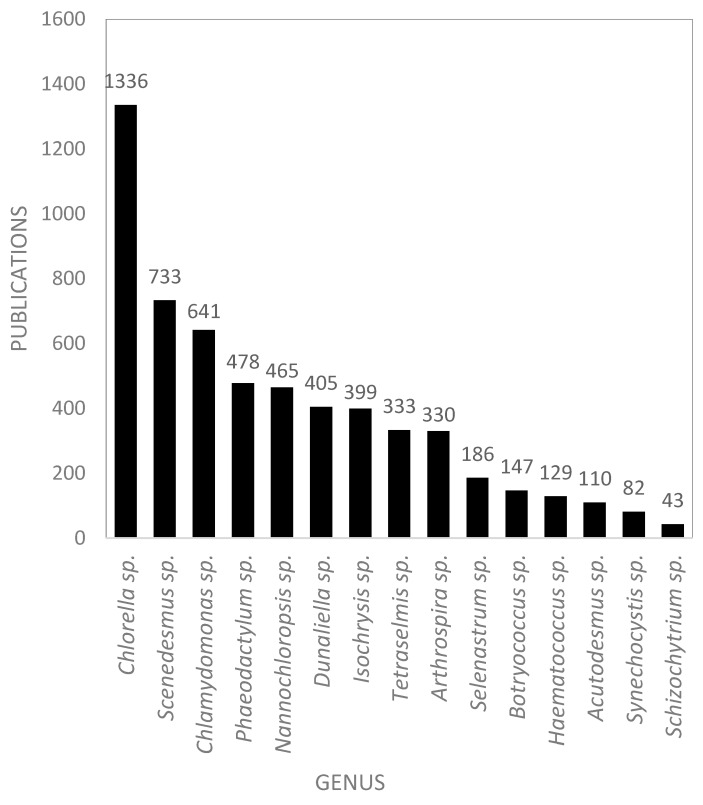
Top 15 microalgae and cyanobacteria genera in European scientific publications.

Figure 7 shows the ranking of the 15 most published genera in scientific publications in Europe. With 1336 publications, *Chlorella* sp. is the most published genus with almost twice as many publications as *Scenedesmus* sp. (733) and *Chlamydomonas* sp. (641). For each of genera, the corresponding emerging concepts are listed in Table 4. These concepts highlight the recent applications and research in the last two years, as well as the growth factors of these concepts. For example, in the 1336 publications of *Chlorella sp*., the top emerging concepts were *biostimulant* and *agro industrial waste* (Table 4).

It should be noted that most microalgae species studied in research labs and their extracts are not authorized for commercialization to consumers by European or foreign regulations. These logically include toxic dinoflagellates and toxic diatoms, but also the vast majority of microalgae and cyanobacteria species whose safety for humans has not been assessed yet. Additionally, specific national or international regulations limit the number of species authorized for some applications (e.g., cosmetics in China—personal communication of the President of the European Algae Biomass Association—or Generally Recognized As Safe (GRAS) species).

#### 2.2.6. Focus on the Top 15 Studied Genera in Europe 

Following the identification of the top 15 microalgae genera studied in Europe, a detailed study of associated and emerging concepts, scientific consortia working on these genera in Europe, research cities, temporal evolution of publications by country, top journals, citations, and top cited papers was performed. The results of this advanced analysis are presented below for *Chlorella* sp. in Figure 8 and equivalent figures for the 14 remaining genera are presented in the Supplementary Materials section in Figures S1–S14. 

### 2.3. Potential of the European Atlantic Area

The EnhanceMicroalgae project is a transnational and inter-regional Atlantic project that aims to evaluate the potential of the European AA in the microalgae industrial sector [7] (https://www.enhancemicroalgae.eu/). To compare on a global scale, 9% of world publications came from the AA, 15% of world authors were from the AA, and 37% of affiliations working in the field of microalgae were located in the AA. In this section, the AA was compared with Europe for (i) the main concepts and topics of scientific publications, (ii) the national contribution to publications, (iii) the networks of collaborations by city and country, as well as (iv) the main journals and citations in the field of microalgae. 

#### 2.3.1. Research Concepts

**Figure 9:**
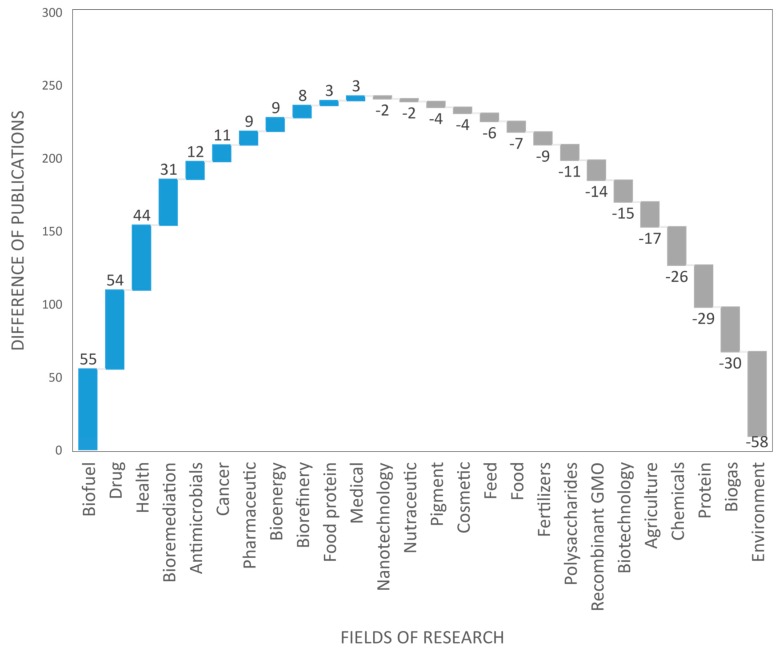
Difference in the number of publications in the European AA by research field between the publications in AA and the publications in the European database (for example: the European AA published more in the fields of biofuel/drug and less in the fields of environment/biogas than the European trend). GMO: Genetically-Modified Organism.

**Figure 10:**
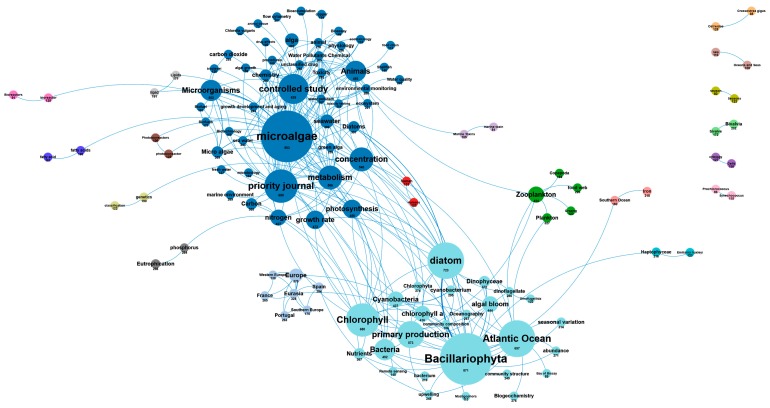
Main concepts networks and importance of concepts in the 6989 AA scientific publications.

The AA database contains 6989 scientific publications with an increasing annual production that reached 509 publications in 2018. The main fields of research were analyzed in this section and compared with the overall European research. Figure 6 shows the number of scientific publications by research field within Europe. By comparing these research fields to those of the AA, Figure 9 highlights differences in the number of publications dealing with biofuels, health, cosmetics, agriculture, and highlights the most published research (on the left of the chart) and the least published (on the right of the chart) in the AA compared to the European research fields. The AA researchers were more interested in research topics such as *biofuel* (+55 publications), *drug* (+54), *health* (+44), and *bioremediation* (+31). In contrast, research topics such as *environment* (-58), *biogas* (-30), *protein* (-29), and *chemicals* (-26) were less published in the AA as compared to the European average. Other research fields ranging from +10 to -10 are in line with European research priorities (Figure 9). The concepts network in the AA publications highlights the dominant regional scientific axes that group in thematic research clusters (Figure 10). The main research topics in the European AA are in line with the most relevant industrial activities in the area, where drug development and health, biofuel, and bioremediation from agricultural activities are massive industries.

#### 2.3.2. Emerging Concepts Related to High Added-Value Microalgae Molecules in the Atlantic Area

**Figure 11:**
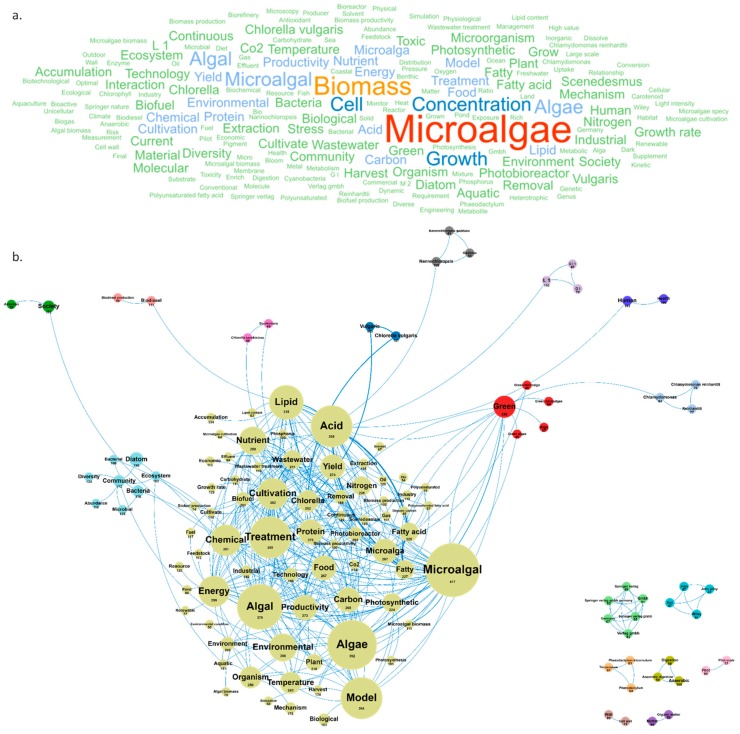
Main concepts (**a**) and concepts network (**b**) in 1882 AA publications published since 2017 related to high-added value microalgae molecules (400 links; 44 co-occurrences; 45 occurrences; 18 clusters).

This last section shows the results of a focused bibliometric analysis on scientific publications produced during the last 3 years in the European AA, excluding publications related to the environment/phytoplankton field. A total of 3393 papers were published between 2017 and 2019, and 1882 deal with microalgae high added-value molecules. Among these publications, 637 were published in 2017, 1005 in 2018, and 232 until February 2019. The two journals dominating this topic at the AA level (as well as at the European level) are *Algal Research* with 151 publications and *Bioresource Technology* with 87 publications. From a geographical point of view, analysis of the countries and cities producing these recent publications reveals that many of them are located outside the European AA and therefore published with co-authors from the European AA. The four major publishing countries, with more than 300 publications, were Spain (374), Italy (325), Germany (322), and France (310), followed by Portugal, the Netherlands, the U.S., Belgium, and the United Kingdom that have published each between 100 and 150 publications on microalgae high added-value molecules since 2017. The major AA cities working in this field are Wageningen, Paris, Rome, Lisbon, Barcelona, Almeria, and Nantes with 85, 66, 64, 63, 58, 57, and 50 publications including at least one co-author in the AA, respectively. As shown in Figure 11, the main concepts emerging from this AA database are *microalgae*/*biomass*/*growth*/*cell*/*concentration*. By dismissing these ubiquitous concepts, a network of 18 clusters was created in which *model*, *treatment*, *acid*, *lipid*, *chemical*, *energy*, *nutrient*, *cultivation*, *green* and *carbon* were the dominant concepts (Figure 11).

#### 2.3.3. National Contribution to the AA Publications

**Figure 12:**
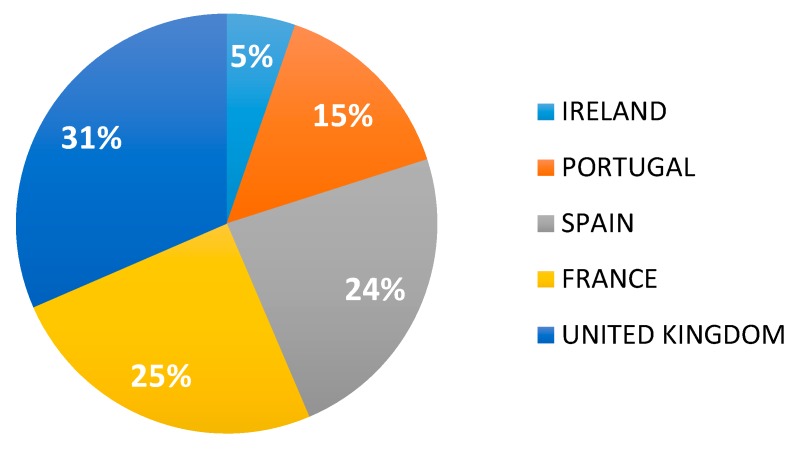
Contribution of the European AA countries in microalgae publications.

Regardless of their size, the contribution of each country for publications in the AA, namely, Ireland, the United Kingdom, France, Spain, and Portugal, was compared in this section. The United Kingdom mainly contributed to these publications, as the United Kingdom affiliations were identified in 33% of the publications compared to 25% and 24% for France and Spain, respectively. Portugal produced 15% of the scientific publications in the European Area and Ireland was associated with 5% of the publications (Figure 12).

#### 2.3.4. International Partnership with the European Atlantic Area

**Figure 13:**
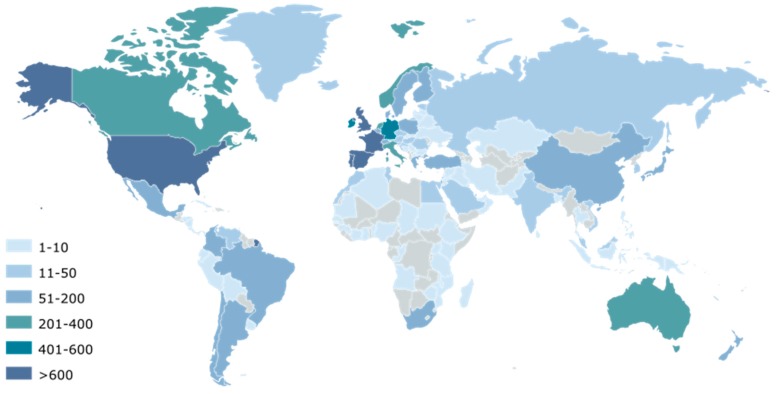
Main countries identified as scientific collaborators in the microalgae European AA publications.

The international scientific collaborations of the European AA were analyzed in this section in order to highlight strong collaborations but also to identify collaborations that could be developed for a better sharing of research at geographical and thematic levels. Figure 13 and Table 5 show the world collaborators associated to scientific publications of the European AA. Beyond a strong national network for the United Kingdom, France, Spain, and Portugal with 2484, 1961, 1855, and 1164 publications, respectively, the AA published numerous publications with the U.S. (836), Germany (494), Canada (275), Netherlands (252), Italy (242), Australia (231), and Norway (230). The AA has also developed contacts with most other European countries, but these networks have a low scientific productivity, with only 1 to 10 joint papers.

**Figure 14:**
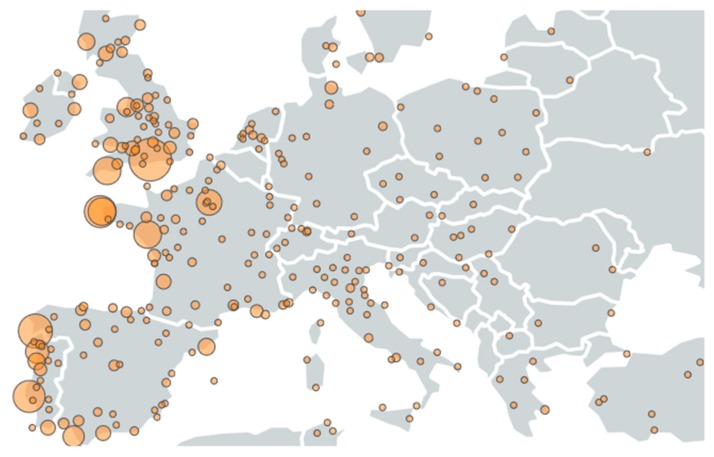
Main cities identified as scientific collaborators in the microalgae European AA publications. Some important publishing cities may not appear on this map (e.g., Bremerhaven) because they are not indexed in the mapping system of the Intellixir software.

**Figure 15:**
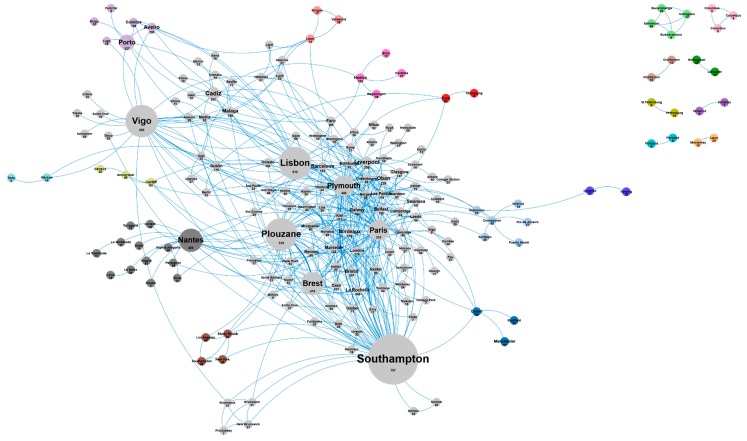
Collaboration networks of scientific collaborators in the microalgae European AA publications (500 links; 5 co-occurrences; 5 occurrences; 20 clusters).

Figure 14 and Table 6 show the main AA cities identified as scientific collaborators in the microalgae European AA publications. With 707 publications, Southampton is the city with the highest number of publications in the AA, followed by Vigo, Plouzané, Lisbon, Brest, Nantes, and Plymouth as the most productive AA cities, with more than 500 publications each. Paris is the first city outside the AA to publish the most publications with AA co-authors (375 publications). The network of all these collaborations is shown in Figure 15.

#### 2.3.5. Journals

This section highlights the main journals and citations from AA publications (Table 7). The main journals in which AA authors publish are *Marine Ecology Progress Series*, *Estuarine Coastal and Shelf Science*, and *Journal of Plankton Research* with 263, 209, and 193 publications, respectively. As observed in the European database, the journals publishing the highest number of microalgae AA studies are related to environmental/phytoplankton research. The main journals publishing papers related to microalgae/high-added value molecules are *Bioresource Technology* and *Algal Research*-*Biomass Biofuels and Bioproducts*, with impact factors of 5.8 and 3.7, respectively, and *Progress in Oceanography* (IF 4.27). Despite the dominance of environmental science journals, the two scientific publications with the highest citations in the AA deal with the production of high-added value molecules from microalgae and are entitled “Microalgae for biodiesel production and other applications: A review” and “Biofuels from microalgae-A review of technologies for production, processing and extractions of biofuels and co-products“. These papers were published in 2010 and cited 2610 times and 2124 times, respectively (Table 8). 

#### 2.3.6. Main Species Studied in the AA

**Figure 16:**
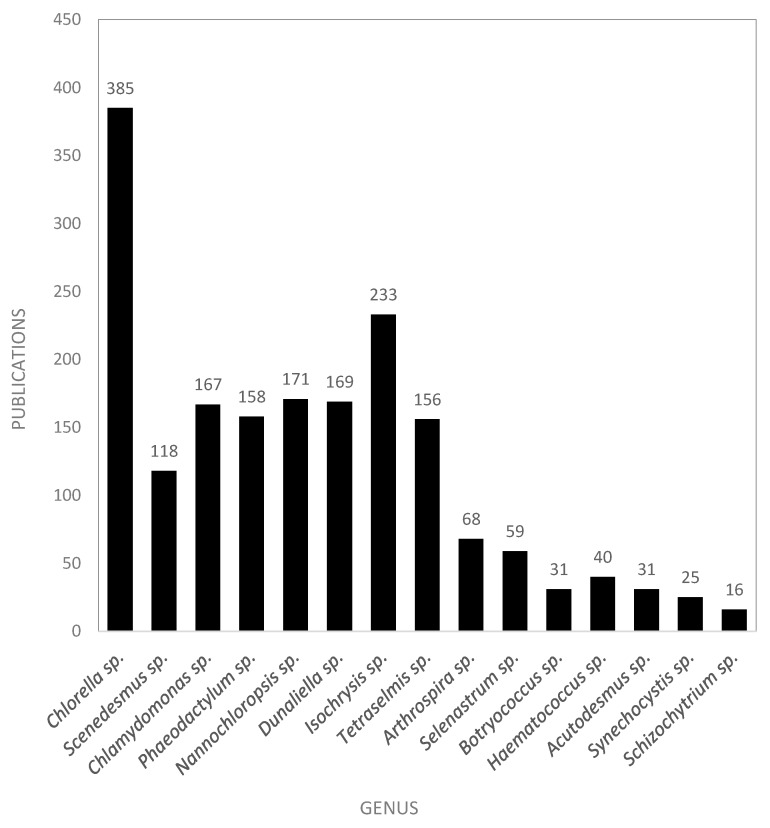
Top 15 microalgae and cyanobacteria genera published in the European AA scientific publications.

Figure 16 shows the number of scientific publications by microalgae genus in the European AA. This figure can be compared with Figure 7 that shows comparable data at the European level. The most published and studied genus in the AA, as well as in the European database, was *Chlorella* genus, with 835 publications. This high citation index can be explained because it is a well-known GRAS alga, and this species has been commercialized worldwide since decades ago. *Isochrysis* sp. was the second most studied, with 233 total publications, whereas it ranked seventh at the European level. This difference may be explained by highest investments of the AA for aquaculture-related research and feeding of bivalve larvae, because of its proximity to the Atlantic coast. *Chlamydomonas reinhardtii*, *Phaeodactylum tricornutum*, *Nannochloropsis oculata*, *Dunaliella salina*, and *Tetraselmis suecica* species were also widely studied in the AA, with about 150 scientific papers for each published until February 2019. The majority of scientific studies on *Scenedesmus* sp. were published from Spain (particularly from Almeria), Germany, Italy, and the Netherlands (particularly from Wageningen).

## 3. Materials and Methods

### 3.1. Building the Bibliographic Database

**Figure 17:**
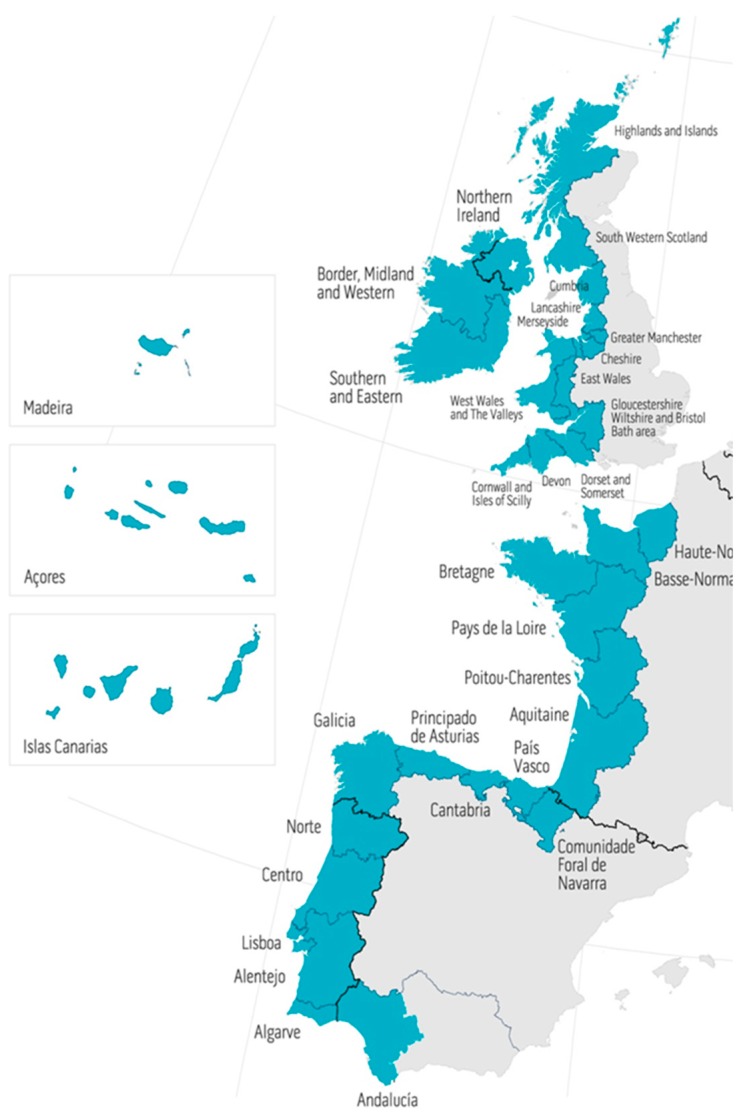
The European AA, as defined in the InterReg AA research program. This area, identified in blue on the map, includes administrative regions bordering the Atlantic Ocean in Portugal, Spain, France, the United Kingdom, and Ireland.

A bibliographic database was built through a literature search performed in February 2019 including all reports published to date. The use of the Scopus database was compulsory to obtain a format compatible with the bibliometric analysis using the Orbit Intellixir software. The keywords “microalgae” and “phytoplankton” were used to list world publications, European publications (including authors from Austria, Belgium, Bulgaria, Cyprus, Croatia, the Czech Republic, Denmark, Estonia, Finland, France, Germany, Greece, Hungary, Ireland, Italy, Latvia, Lithuania, Luxembourg, Malta, Netherlands, Poland, Portugal, Romania, Slovakia, Slovenia, Spain, Sweden, and the United Kingdom), and European publications including at at least one author from the Atlantic regions of Portugal, Spain, France, the United Kingdom, and Ireland, as defined in the interregional (Interreg) AA research programs (Figure 17). The keywords “microalga(e)” and “phytoplankton” were both selected to include environmental/ecophysiological studies as well as research and development projects dedicated to biotechnological applications for bioremediation, energy, feed, food, cosmetics, and pharma, among others. We did not exclude publications dealing with cyanobacteria, considering that the research domains for these prokaryotic organisms were similar to those of microalgae.

### 3.2. Bibliometric Analysis: Data Extraction, Analysis, and Graphical Formatting

The bibliographic database was last updated in February 2019 and it contained 79,020 publications, 111,975 authors, 4446 affiliations, and 931,299 concepts. These references are available on demand to the corresponding author.

A “concept” designates a word (or group of words) present in the title, summary, or keywords of a publication that can be extracted and identified using a bibliometric software. The occurrence of a concept is the number of documents containing this concept, and co-occurrence the number of documents linking several concepts. The bibliographic database, including references without duplicates, was imported from Scopus (Editor Elsevier) into the Orbit Intellixir bibliometric software and analyzed to quantify the scientific production per year, country, organization, and annual evolution of publication rates. Collaboration networks between countries; public and/or private organizations; as well as major, fading, and emerging research concepts, were graphically represented using the most relevant formats available in the Orbit Intellixir software. Data were analyzed to highlight the latest trends in research topics; identify the most explored research concepts; point out the most studied species; and highlight strengths, opportunities, and collaborations in the research organizations from the European AA. Emerging concepts were defined as concepts that showed the greatest increase in frequency of use in the database over the last 2 years. A manual selection of emerging concepts was performed, as some of them were relevant for our study (e.g., name of molecules, application domains), whereas others were less (publisher name, etc.). A growth factor (GF) was calculated to highlight the concepts with the highest emergence over the past 2 years (2017-2019). GF was calculated as Equation (1).

(1) $$G=\frac{P_{2019}-P_{2017}}{P_{2017}}$$

with *P* being the number of cumulative scientific publications containing the concept at one time.

## 4. Conclusions

On the basis of the analysis of 79,020 publications at the world, European, and European AA levels, our study aimed to give a relevant overview of microalgae research until 2019, an in-depth analysis of research concepts and collaborations, and a European perspective on emerging topics. Studied microalgae species were also analyzed in detail to highlight their associated concepts, the networks of researchers working in the field, and the potential of development for new applications or new species. The scientific research topics were essentially the same at the world, European, and AA levels, with the main research concepts corresponding to *phytoplankton*, *community*, *bloom*, *diatoms*, *distribution*, *ecosystem*, *coastal*, *chlorophyll*, *zooplankton*, *photosynthesis*, and *primary production*. Focusing at the European AA level in which the Interreg EnhanceMicroalgae project is positioned, the analysis of the 6989 publications revealed that the *biofuel*, *drug*, *health*, and *bioremediation* topics are more published than at the European average. The major publishers of these AA publications were located in the United Kingdom, France, and Spain, particularly in research poles of Southampton, Vigo, Plouzané, Lisbon, Brest, Nantes, and Plymouth. In contrast, compared to Europe, the AA produced less scientific publications in the field of microalgae-based high added-value molecules (1882 publications), suggesting a scope to extend in term of research, collaboration, and industrial development. The highest scientific trends and market opportunities were highlighted by identifying top emerging concepts such as *bioplastics* and *biostimulants* at the European level and *agricultural, animal feed*, and *blue biotechnology* at the European AA level. By focusing at the European scale, the most studied topics found in the 26,137 European publications were related to the *environment*, *food*, *chemicals*, *pigments*, *protein*, *feed*, and *drugs*. Analysis of international cooperation highlighted the strong links and partnerships with the United States, Canada, Norway, and Australia, but identified the possible development of new collaborations with most other countries. Within the European Union, France, Germany, and Spain dominated the scientific productivity, and the most studied and published species were *Chlorella* sp. and *Scenesdesmus* sp. A large number of microalgae and cyanobacteria species have received minimal attention, indicating a significant innovation potential for new molecules, new applications, and markets. Finally, this study provides an updated review of quantitative data at three different reading levels, and should allow microalgae stakeholders to guide their investments and projects for future research opportunities and cooperation in the field of microalgae research and bioeconomy.

## Figures and Tables

**Figure 8:**
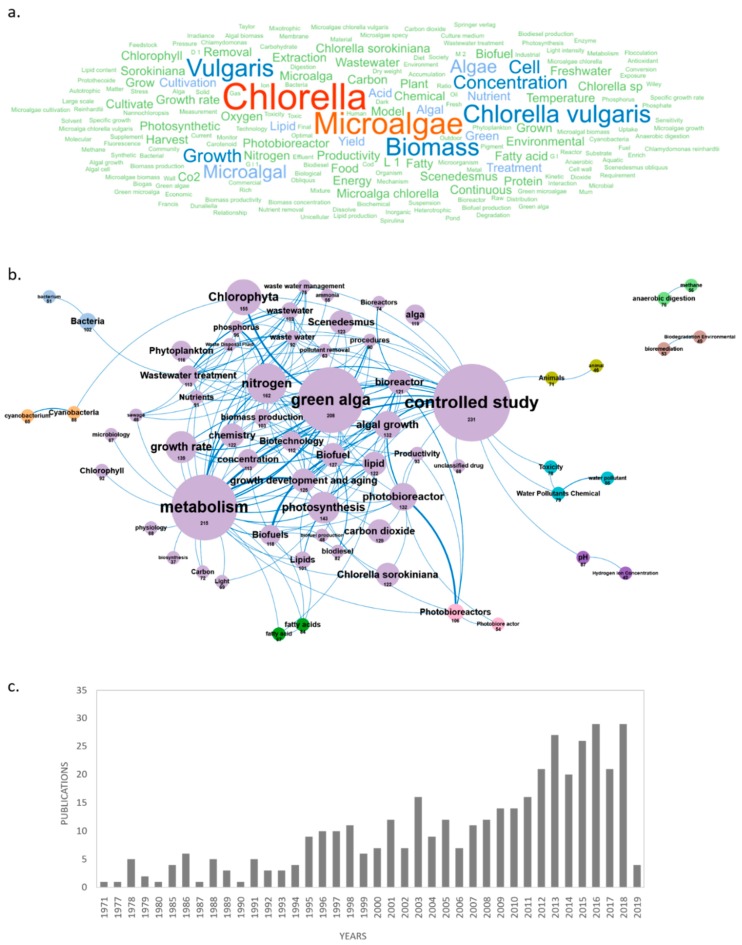

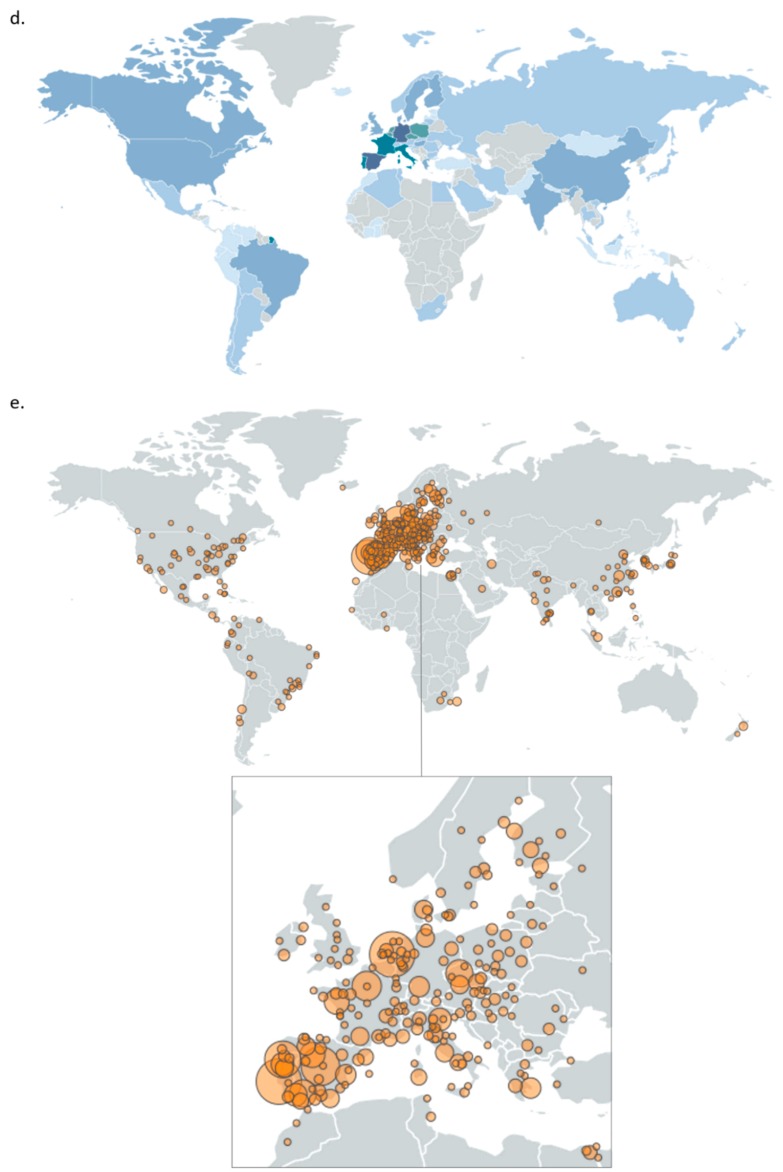

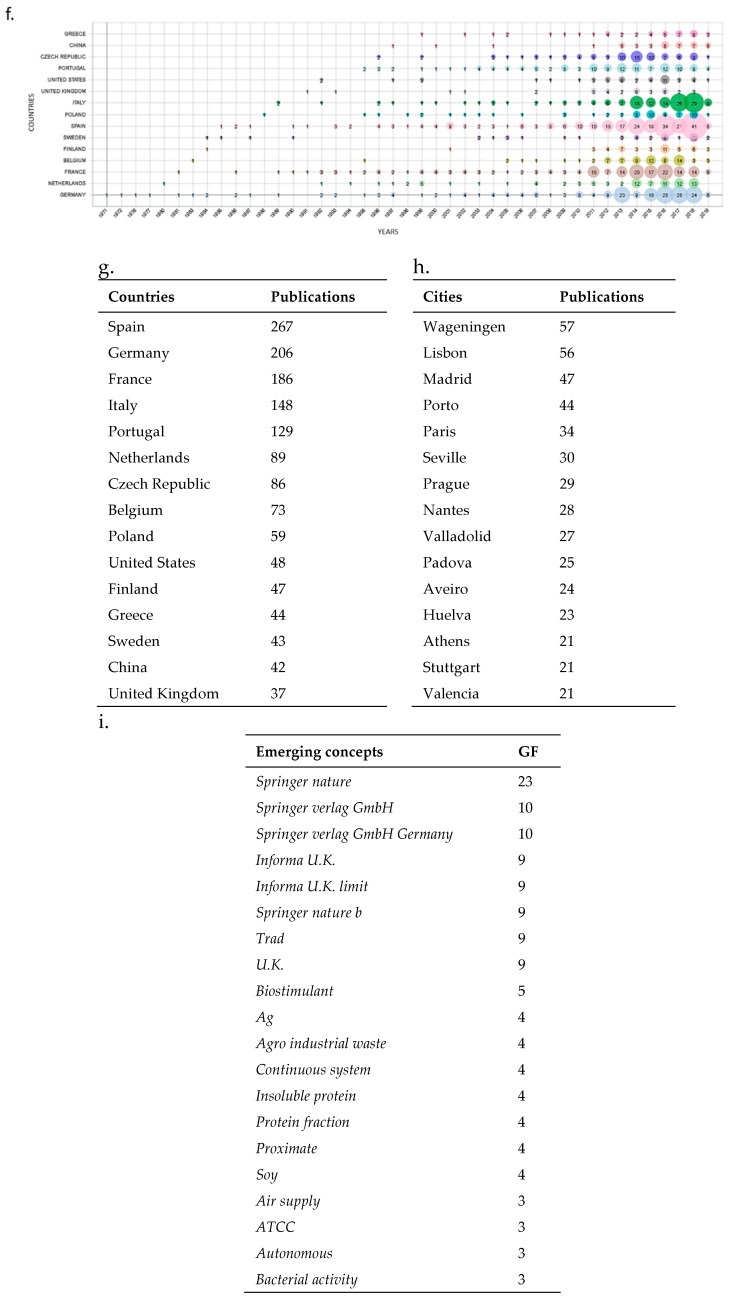

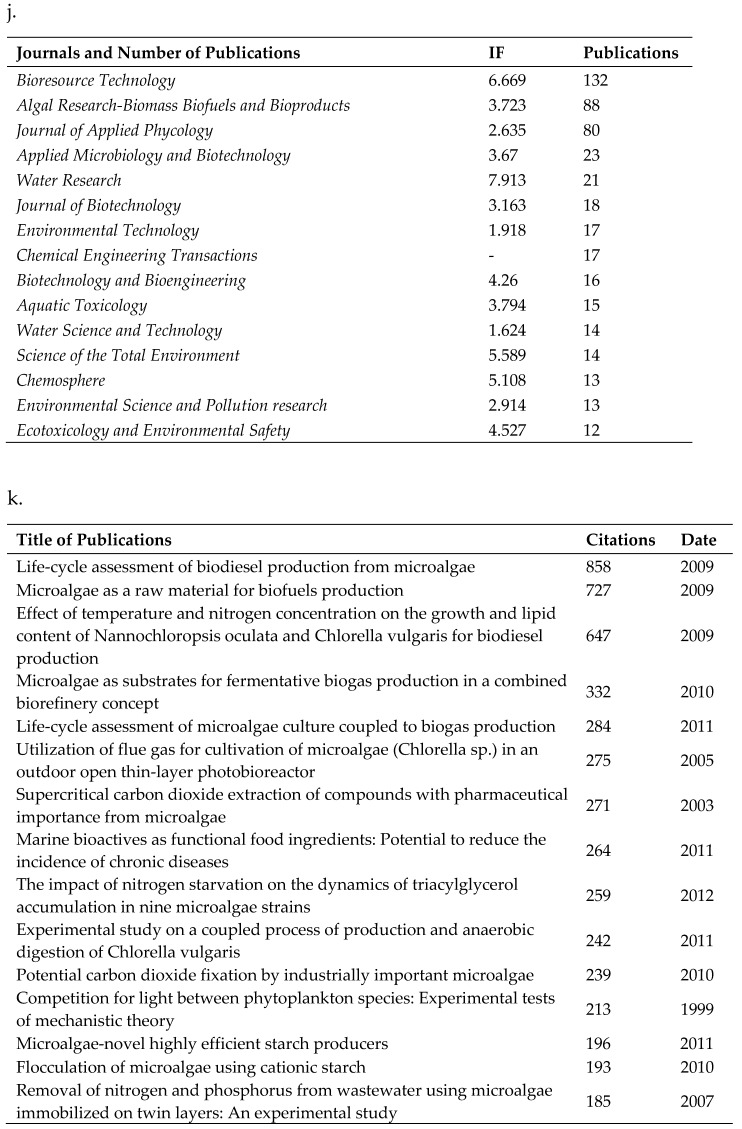
Bibliometric overview of the research on *Chlorella* sp. in 1336 European scientific papers. Main concepts (**a**), concepts network (**b**), annual production (**c**), global collaborations (**d**), European collaborations (**e**), annual production by countries (**f**), main countries (**g**), main cities (**h**), emerging concepts (**i**), main journals (**j**), and main citations (**k**).

**Table 1:** Number of publications, authors, affiliations, and concepts in the three databases.

Database	Publications	Authors	Affiliations	Concepts
World	79,020	111,975	4446	931,299
Europe	26,137	46,789	2393	423,567
AA	6989	17,304	1657	163,218

**Table 2:** Top emerging concepts in 2017–2019 and growth factors (GF) identified in the microalgae scientific publications in the world database, the European database, and the AA database.

Concepts in the WORLD	GF	Concepts in EUROPE	GF	Concepts in the AA	GF
Feed	11,192	Feed	3562	Feed	1007
Byproduct	7	Marine alga	12	Cell	13
Cell	7	Resource recovery	8	Bovine	5
Hypoxic conditions	7	Cell	5	Cattle	4
Body weight gain	6	Cell component	5	Fatty acid ester	4
Bio surfactant	5	Contaminants of emerging concerns	5	Marine alga	4
Contaminants of emerging concerns	5	Omics	5	Symbiodinium	4
CRISPR/CAS9	5	Bioplastics	4	Veterinary medicine	4
Marine oil snow	5	Biostimulants	4	Blue biotechnology	3
Mechanical pre treatment	5	EC10	4	Cell component	3
Recombinant enzyme	5	Genome editing	4	Circular economy	3
Transcription factor Nrf2	5	Surf zone	4	Cryptophytes	3
		Tropical forest	4	Dietary exposure	3

**Table 3:** Main journals publishing microalgae research in Europe, impact factors, and number of publications.

Journals	Impact Factor	Number of Publications
*Hydrobiologia*	2.165	1162
*Marine Ecology Progress Series*	2.276	783
*Journal of Plankton Research*	1.897	614
*Estuarine Coastal and Shelf Science*	2.413	473
*Bioresource Technology*	5.807	416
*Limnology and Oceanography*	3.595	416
*Journal of Marine Systems*	2.506	380
*Biogeosciences*	3.441	350
*Science of the Total Environment*	4.61	349
*Journal of Applied Phycology*	2.401	335
*Algal Research-Biomass Biofuels and Bioproducts*	3.745	334
*Freshwater Biology*	3.767	321
*Aquatic Microbioal Ecology*	2.024	298
*Marine Biology*	2.215	289
*Deep-sea Research Part II-Topical studies in Oceanography*	2.451	286
*PLoS One*	2.766	276
*Journal of Experimental Marine Biology and Ecology*	1.99	257
*Ecological Modelling*	2.507	233
*Aquaculture*	2.71	209

**Table 4:** Top 15 emerging concepts in 2017-2019 and growth factors (GF) identified for the major studied genera in European microalgae and cyanobacteria scientific publications.

**Emerging Concepts**																
***Chlorella* sp.**		***Scenedesmus* sp.**			***Chlamydomonas* sp.**		***Phaeodactylum* sp.**		***Nannochloropsis* sp.**		***Dunaliella* sp.**		***Isochrysis* sp.**		***Tetraselmis* sp.**	
Springer nature	23	Springer nature		14	Friendly	5	Springer nature	7	Springer nature	8	Ag	3	Springer nature	10	Single species	4
Springer verlag GmbH	10	Springer verlag GmbH		9	Springer nature	5	Edit	5	Switzerland	5	Membrane filtration	3	Almeriensis	4	Bass	3
Springer verlag GmbH germany	10	Springer verlag GmbH germany		7	Harbor	4	Genome edit	5	Basel	4	Pilot scale	3	Blend	4	Dicentrarchus labrax	3
Informa uk	9	Tetradesmus		5	Hinder	4	pH value	5	Farm	4	Raceway	3	Isolipidic	4	European sea bass	3
Informa uk limit	9	Tetradesmus obliquus		5	Intron	4	Cas9	4	John	4	Affinis	2	Microalga Tisochrysis lutea	4	Labrax	3
Springer nature b	9	Biostimulant		4	Confocal	3	Crispr	4	John Wiley	4	Algal pond	2	Aminopeptidase	3	Sea bass	3
Trad	9	Informa		4	Cyclase	3	Crispr cas9	4	Maintenance	4	Algal productivity	2	Anti-inflammatory	3	Share	3
U.K.	9	Informa U.K.		4	ELISA	3	Effector	4	Son	4	Algal productivity model	2	Functionality	3	Alkaline phosphatase	2
Biostimulant	5	Informa U.K. limit		4	European society	3	mRNA	4	Bioavailability	3	Bioactivity	2	Germany	3	Anthropogenic	2
Ag	4	Livestock		4	Green cell factory	3	pH 8	4	Coal	3	Biomass concentration	2	Oxidative stress	3	Associate bacteria	2
Agro industrial waste	4	Phosphorus removal rate		4	Isoprenoid	3	Bioactivity	3	Economy	3	Cell disruption	2	Pesticide	3	Bacterial community	2
Continuous system	4	Root		4	Membrane bioreactor	3	Delivery	3	Fishery	3	Cheaper	2	Potential effect	3	Biotechnological application	2
Insoluble protein	4	Tailor		4	Microalgae population	3	Drug	3	Food chain	3	Combine diet	2	Protease	3	Blend	2
Protein fraction	4	Trad		4	Photobiology	3	Nannochloropsis oceanica	3	Gamma	3	Dry sample	2	Scale production	3	Call	2
Proximate	4	Uk		4	Photobiology 2018	3	Native	3	Human consumption	3	Dynamic filtration	2	Scenedesmus almeriensis	3	Chlorella sorokiniana	2
Soy	4	Batch operation		3	Proline	3	Overview	3	Nan	3	Elongase	2	Senegalensis	3	CO_2_	2
Air supply	3	Biomass grown		3	RNA-seq data	3	Volumetric productivity	3	Nannochloropsis oceanica ccmp1779	3	Eventually	2	Senegalese	3	CO_2_ enrichment	2
ATCC	3	Centrate		3	Surprisal analysis	3	Algal strain	2	Oceanica ccmp1779	3	Explosion	2	Solea	3	CO_2_ injection	2
Autonomous	3	Corn		3	Agar plate	2	Architecture	2	Render	3	Final concentration	2	Solea senegalensis	3	Complete diet	2
Bacterial activity	3	Cylindrical		3	Alternative strategy	2	Autofluorescence	2	Separately	3	Food supplement	2	Springer verlag GmbH	3	Consecutive	2
**Growth factors**																
***Arthrospira* sp.**			***Selenastrum* sp.**				***Botryococcus* sp.**		***Haematococcus* sp.**		***Acutodesmus* sp.**		***Synechocystis* sp.**		***Schizochytrium* sp.**	
Springer nature		5	Biochemical			3	Absorption	2	Diverse	4	Total Phosphorus	5	Springer nature	3	Bioactive	2
Alternative protein		4	Freshwater microalga			3	Fatty acid composition	2	Medicine	4	Cod	4	Highest value	2	Chemical	2
Alternative protein source		4	Additivity			2	Accumulation response	1	Bar	3	Cell number	3	Pharmaceutical	2	Concentrate	2
Bean		4	Algal culture			2	Adaptive	1	Microalgal specy	3	Continuous mode	3	Production process	2	Consume	2
Proximate		4	Amend			2	Adaptive cell response	1	Nutraceutic	3	Dynamic	3	Springer nature b	2	Enzymatic	2
Soy		4	Biochemical composition			2	a DNA	1	Almeriensis	2	Exogenous	3	Synthase	2	Food application	2
Stream		4	Biomass production			2	a DNA sequence	1	Aquatic	2	Tn	3	Technological	2	Glycerol	2
Substitution		4	Calibrate			2	Algae cultivation	1	Art	2	Aeration	2	Acclimation	1	Limacinum	2
Biomass cultivation		3	DHA			2	Algae species	1	Bench	2	Antibacterial	2	Acclimation process	1	Microalgal oil	2
Continuous system		3	Ecosar			2	Algal system	1	Bench scale	2	Aquatic organism	2	Acid phosphatase	1	N-6 PUFA	2
Corn		3	Euglena			2	Alkaline medium	1	Bench scale reactor	2	Ascorbate	2	Acid phosphatase activity	1	NMR	2
Differential		3	Euglena gracilis			2	Ally	1	Biofilm	2	Ascorbate peroxidase	2	Acting	1	Phaeodactylum	2
Energy return		3	Fate			2	Alpha linolenic	1	Carbohydrate	2	Auxin	2	Adaptation process	1	Porphyridium	2
Fourier		3	Freshwater microalga Pseudokirchneriella subcapitata			2	Alpha linolenic acid	1	Carotenoid extraction	2	Batch operation	2	Adhere	1	Pure	2
Fourier transform		3	Friendly			2	Analize	1	Chloroform	2	Bioaccumulation	2	Adhesion	1	Rapeseed	2
Gastrointestinal		3	Gracilis			2	Ancient	1	Contaminant	2	Bioenergy	2	Adhesion assay	1	Rapeseed oil	2
Glycerol		3	Macrolide			2	Ancient DNA	1	Contamination	2	Bioremediation	2	Adsorbent	1	Regardless	2
Grain		3	Macrolide antibiotic			2	Aquaculture	1	Crucial	2	Breed	2	Adsorbent material	1	Rhizomucor *miehei*	2
Grass		3	Mix algal			2	Aquaculture effluent	1	Deal	2	Carotene	2	Adsorption	1	Ruminant	2
Hydrothermal liquefaction		3	Mix algal culture			2	Aquaculture production	1	Delivery	2	Catalase	2	Adsorptive	1	Schizochytrium limacinum	2

**Table 5:** Top 16 countries identified as scientific collaborators in the microalgae European AA publications and numbers of publications.

Countries	Publications
United Kingdom	2484
France	1961
Spain	1855
Portugal	1164
United States	836
Germany	494
Ireland	416
Canada	275
Netherlands	252
Italy	242
Australia	231
Norway	230
Brazil	164
Belgium	137
Denmark	134
Sweden	119

**Table 6:** Top 20 cities identified as scientific collaborators in the microalgae European AA publications and numbers of publications.

Cities	Publications
Southampton	707
Vigo	530
Plouzané	518
Lisbon	515
Brest	419
Nantes	409
Plymouth	406
Paris	375
Porto	327
Cadiz	302
Liverpool	255
Oban	228
Aveiro	200
Barcelona	193
Bordeaux	170
Malaga	168
Faro	159
Bristol	157
Galway	154
Belfast	150

**Table 7:** Main journals publishing microalgae research in the AA, impact factors, and number of publications.

Journals	Impact Factor	Number of Publications
*Marine Ecology Progress Series*	2.276	263
*Estuarine Coastal and Shelf Science*	2.413	209
*Journal of Plankton research*	1.897	193
*Deep-Sea research Part II-Topical Studies in Oceanography*	2.451	157
*Hydrobiologia*	2.165	144
*Bioresource Technology*	5.807	129
*Journal of Experimental Marine Biology and Ecology*	1.99	126
*Journal of Marine Systems*	2.506	119
*Biogeosciences*	3.441	111
*Limnology and Oceanography*	3.595	107
*Journal of Applied Phycology*	2.401	107
*Aquaculture*	2.71	101
*Progress in Oceanography*	4.27	99
*Algal Research-Biomass Biofuels and Bioproducts*	3.745	92
*Deep-Sea research Part I-Oceanographic Research Papers*	2.384	85

**Table 8:** Main microalgae publications cited in the AA.

Title of Publications	Citations	Reference
Microalgae for biodiesel production and other applications: A review	2610	[8]
Biofuels from microalgae-A review of technologies for production, processing, and extractions of biofuels and co-products	2124	[9]
Microbial carbonates: The geological record of calcified bacterial-algal mats and biofilms	847	[10]
Comparative toxicity of nanoparticulate ZnO, bulk ZnO, and ZnCl2 to a freshwater microalga (*Pseudokirchneriella subcapitata*): The importance of particle solubility	829	[11]
The relative influences of nitrogen and phosphorus on oceanic primary production	827	[12]
Mesoscale iron enrichment experiments 1993-2005: Synthesis and future directions	787	[13]
The potential of sustainable algal biofuel production using wastewater resources	737	[14]
Microalgae as a raw material for biofuels production	727	[15]
Oceanic 18S rDNA sequences from picoplankton reveal unsuspected eukaryotic diversity	643	[16]
Increase in *Chlorella* strains calorific values when grown in low nitrogen medium	623	[17]
Microalgae as biodiesel & biomass feedstocks: Review & analysis of the biochemistry, energetics & economics	615	[18]
Lake responses to reduced nutrient loading - An analysis of contemporary long-term data from 35 case studies	615	[19]
Separation of chlorophylls and carotenoids from marine phytoplankton: A new HPLC method using a reversed phase C8 column and pyridine-containing mobile phases	555	[20]
Processes and patterns of oceanic nutrient limitation	530	[21]
North Pacific Gyre Oscillation links ocean climate and ecosystem change	527	[22]

## Supplementary Materials

The following are available online at https://www.mdpi.com/1660-3397/18/2/79/s1, Figure S1–S14: Detailed study of associated and emerging concepts, scientific consortia working in Europe, research cities, temporal evolution of publications by country, top journals, citations, and top cited papers for the genera *Scenedesmus* sp., *Chlamydomonas* sp., *Phaeodactylum* sp., *Nannochloropsis* sp., *Dunaliella* sp., *Tisochrysis* sp., *Tetraselmis* sp., *Arthrospira* sp., *Selenastrum* sp., *Botryococcus* sp., *Haematococcus* sp., *Acutodesmus* sp., *Synechocystis* sp. and *Schizochytrium* sp.

## References

[1] Spolaore P., Joannis-Cassan C., Duran E., Isambert A. (2006). Commercial applications of microalgae. J. Biosci. Bioeng..

[2] Oswald W.J., Gotaas H.B., Golueke C.G., Kellen W.R., Gloyna E.F., Hermann E.R. (1957). Algae in Waste Treatment [with Discussion]. Sew. Ind. Wastes.

[3] Oswald W.J., Golueke C.G., Umbreit W.W. (1960). Biological Transformation of Solar Energy. Advances in Applied Microbiology.

[4] Garrido-Cardenas J.A., Manzano-Agugliaro F., Acien-Fernandez F.G., Molina-Grima E. (2018). Microalgae research worldwide. Algal Res..

[5] Chisti Y. (2007). Biodiesel from microalgae. Biotechnol. Adv..

[6] Stiles W.A.V., Styles D., Chapman S.P., Esteves S., Bywater A., Melville L., Silkina A., Lupatsch I., Fuentes Grünewald C., Lovitt R. (2018). Using microalgae in the circular economy to valorise anaerobic digestate: challenges and opportunities. Bioresour. Technol..

[7] Rumin J., Martins J., Cruz J., Vasconcelos V., Grünewald C.F., Flynn K.J., Sabin A., Paredes M., Conde E., Vilarino J. (2018). EnhanceMicroalgae: An European Interregional Project Stimulating Research, Innovation, Industrial Development and Transnational Cooperation within the Atlantic Area Microalgae Sector. J. Oceanogr. Mar. Res..

[8] Mata T.M., Martins A.A., Caetano Nidia S. (2010). Microalgae for biodiesel production and other applications: A review. Renew. Sustain. Energy Rev..

[9] Brennan L., Owende P. (2010). Biofuels from microalgae—A review of technologies for production, processing, and extractions of biofuels and co-products. Renew. Sustain. Energy Rev..

[10] Riding R. (2000). Microbial carbonates: the geological record of calcified bacterial–algal mats and biofilms. Sedimentology.

[11] Franklin N.M., Rogers N.J., Apte S.C., Batley G.E., Gadd G.E., Casey P.S. (2007). Comparative toxicity of nanoparticulate ZnO, bulk ZnO, and ZnCl2 to a freshwater microalga (Pseudokirchneriella subcapitata): the importance of particle solubility. Environ. Sci. Technol..

[12] Tyrrell T. (1999). The relative influences of nitrogen and phosphorus on oceanic primary production. Nature.

[13] Boyd P.W., Jickells T., Law C.S., Blain S., Boyle E.A., Buesseler K.O., Coale K.H., Cullen J.J., de Baar H.J.W., Follows M. (2007). Mesoscale Iron Enrichment Experiments 1993-2005: Synthesis and Future Directions. Science.

[14] Pittman J.K., Dean A.P., Osundeko O. (2011). The potential of sustainable algal biofuel production using wastewater resources. Bioresour. Technol..

[15] Gouveia L., Oliveira A.C. (2009). Microalgae as a raw material for biofuels production. J. Ind. Microbiol. Biotechnol..

[16] der Staay S.Y.M., Wachter R.D., Vaulot D. (2001). Oceanic 18S rDNA sequences from picoplankton reveal unsuspected eukaryotic diversity. Nature.

[17] Illman A.M., Scragg A.H., Shales S.W. (2000). Increase in Chlorella strains calorific values when grown in low nitrogen medium. Enzyme Microb. Technol..

[18] Williams P.J., Le B., Laurens L.M.L. (2010). Microalgae as biodiesel & biomass feedstocks: Review & analysis of the biochemistry, energetics & economics. Energy Environ. Sci..

[19] Jeppesen E., Søndergaard M., Jensen J.P., Havens K.E., Anneville O., Carvalho L., Coveney M.F., Deneke R., Dokulil M.T., Foy B. (2005). Lake responses to reduced nutrient loading – an analysis of contemporary long-term data from 35 case studies. Freshw. Biol..

[20] Zapata M., Rodríguez F., Garrido J.L. (2000). Separation of chlorophylls and carotenoids from marine phytoplankton: a new HPLC method using a reversed phase C8 column and pyridine-containing mobile phases. Mar. Ecol. Prog. Ser..

[21] Moore C.M., Mills M.M., Arrigo K.R., Berman-Frank I., Bopp L., Boyd P.W., Galbraith E.D., Geider R.J., Guieu C., Jaccard S.L. (2013). Processes and patterns of oceanic nutrient limitation. Nat. Geosci..

[22] Lorenzo E.D., Schneider N., Cobb K.M., Franks P.J.S., Chhak K., Miller A.J., McWilliams J.C., Bograd S.J., Arango H., Curchitser E. (2008). North Pacific Gyre Oscillation links ocean climate and ecosystem change. Geophys. Res. Lett..

