# Supplementary material for: A Bibliometric Analysis of Microalgae Research in the World, Europe, and the European Atlantic Area
Source: Mar Drugs. 2020 Jan 26;18(2):79. doi: 10.3390/md18020079 (PMC7074000; doi:10.3390/md18020079)
Supplement: Supplementary file 1 [file marinedrugs-18-00079-s001.pdf]

***Scenedesmus* sp.**

a.

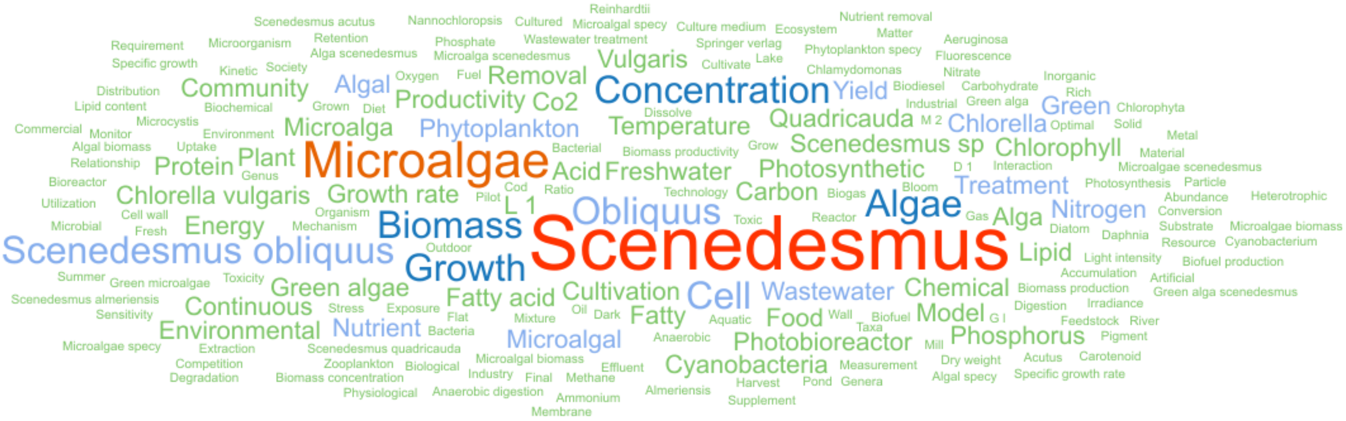

b.

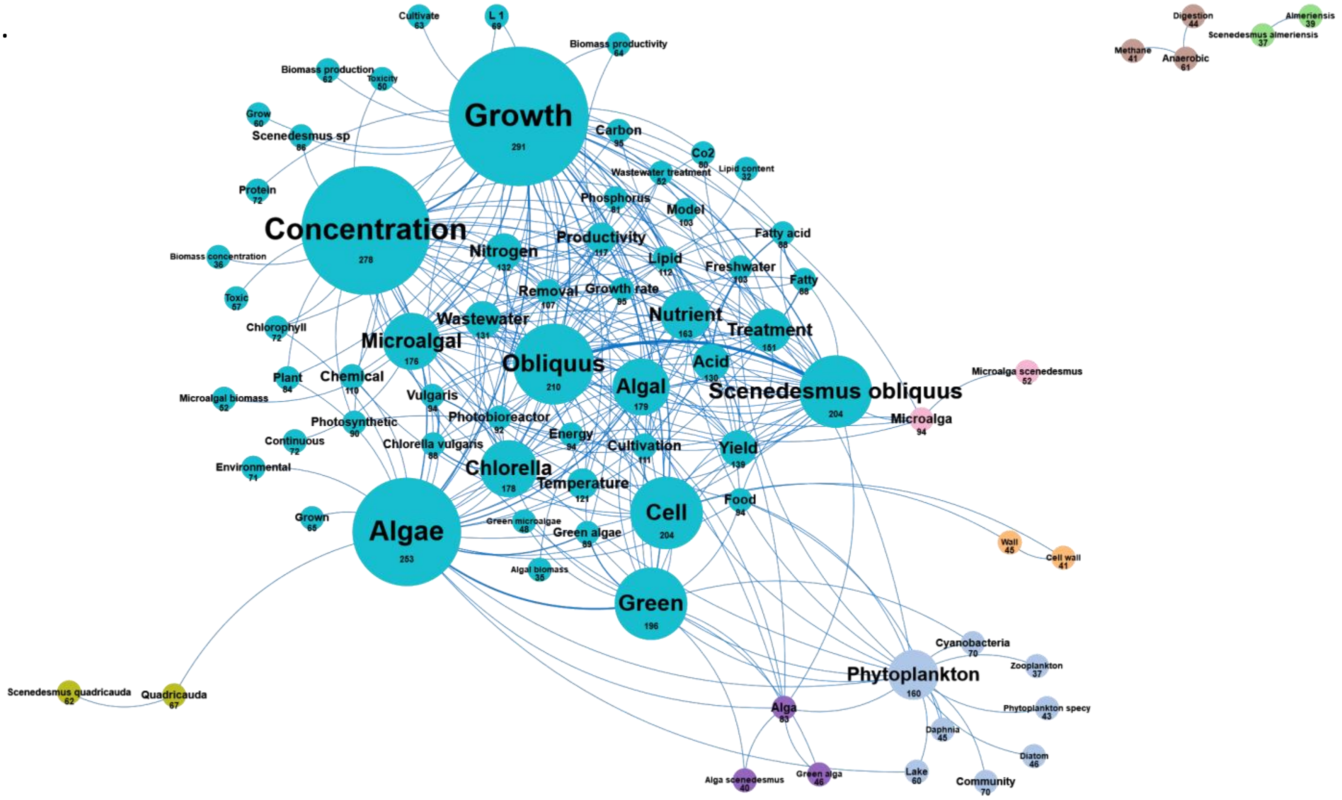

c.

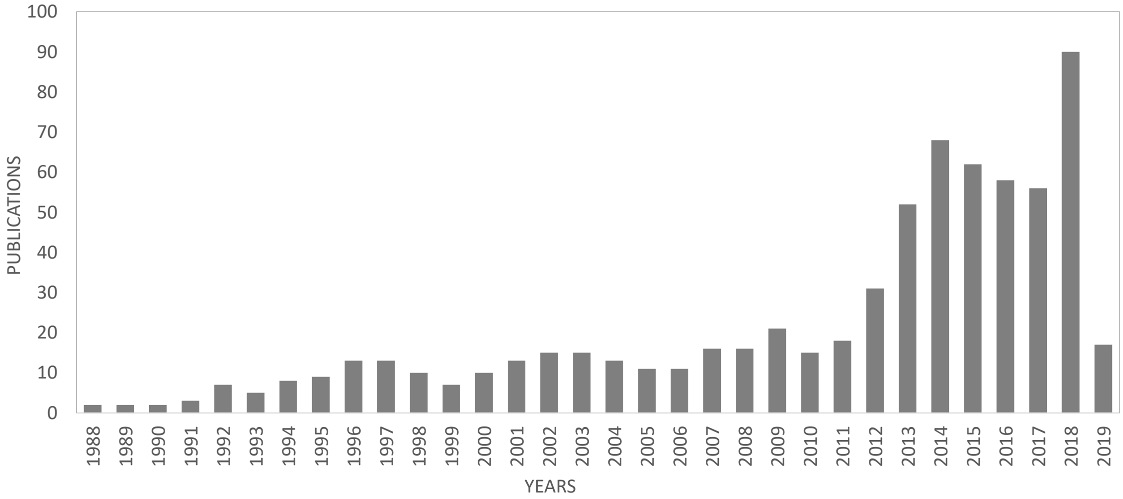

d.

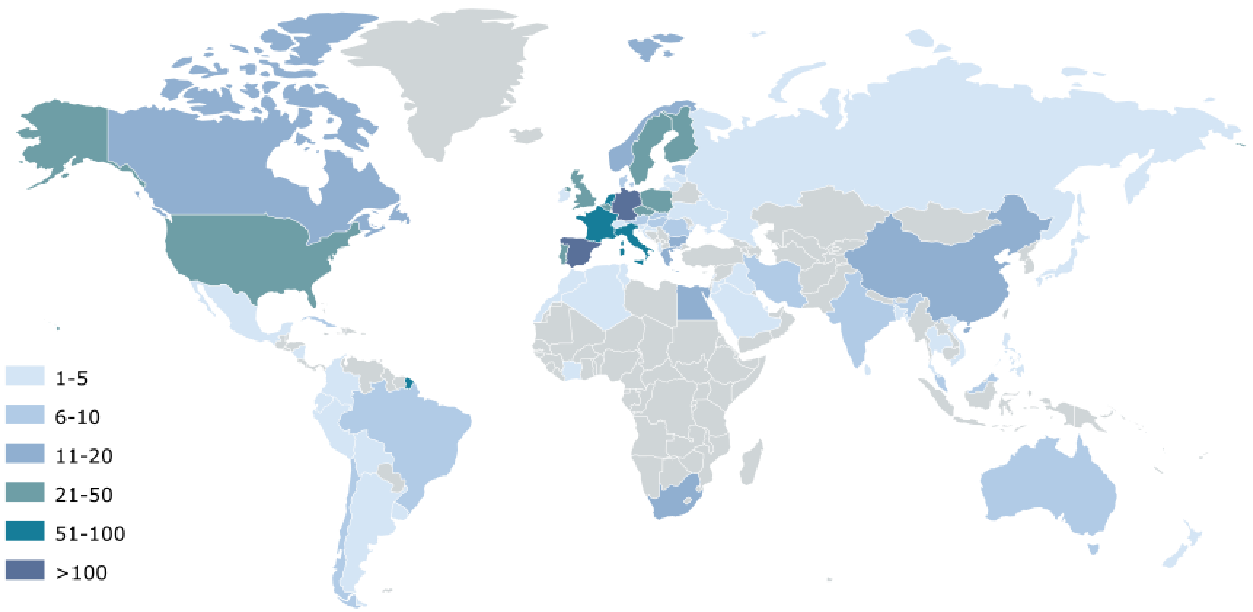

e.

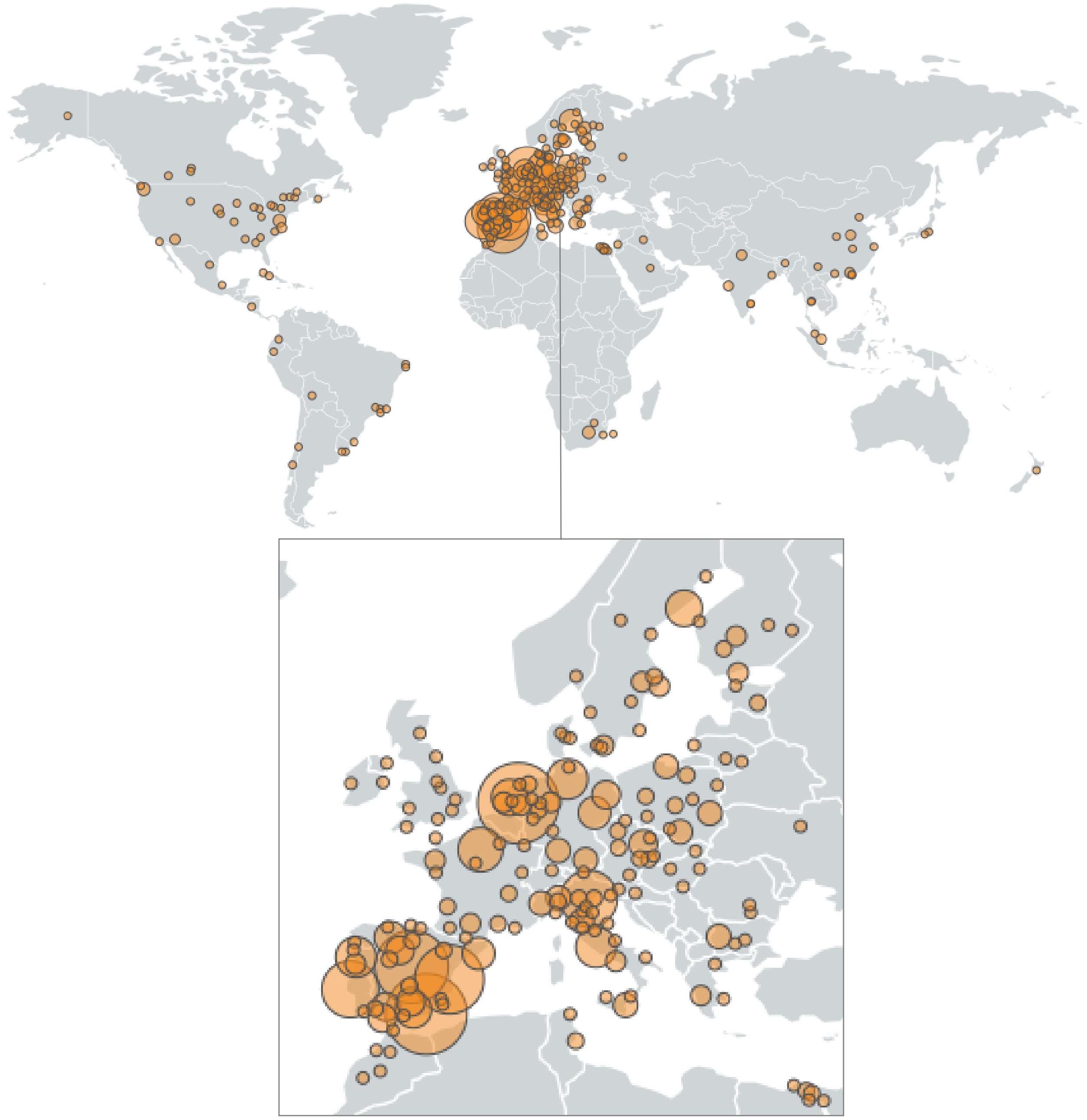

f.

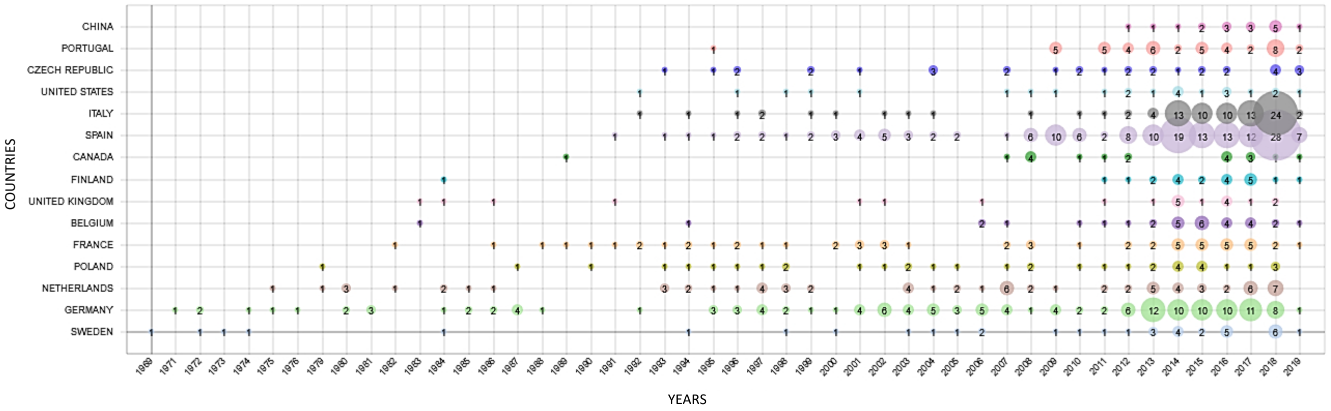

g.

Countries	Publications
SPAIN	165
GERMANY	144
ITALY	91
NETHERLANDS	74
FRANCE	58
PORTUGAL	44
SWEDEN	38
POLAND	37
BELGIUM	32
CZECH REPUBLIC	32
UNITED STATES	24
FINLAND	22
UNITED KINGDOM	22
CANADA	19
CHINA	17

h.

Cities	Publications
Almeria	38
Wageningen	38
Madrid	33
Valencia	31
Lisbon	25
Padova	24
Paris	18
Hamburg	17
Porto	17
Rome	16
Granada	14
Umea	14
Amsterdam	13
Barcelona	11
Leipzig	11

i.

Emerging concepts	GF
Springer nature	14
Springer verlag gmbh	9
Springer verlag gmbh germany	7
Tetradesmus	5
Tetradesmus obliquus	5
Biostimulant	4
Informa	4
Informa uk	4
Informa uk limit	4
Livestock	4
Phosphorus removal rate	4
Root	4
Tailor	4
Trad	4
Uk	4
Batch operation	3
Biomass grown	3
Centrate	3
Corn	3
Cylindrical	3

j.

Journals and number of publications	IF	Publications
BIORESOURCE TECHNOLOGY	6,669	70
JOURNAL OF APPLIED PHYCOLOGY	2,635	34
Algal Research-Biomass Biofuels and Bioproducts	3,723	31
HYDROBIOLOGIA	2,325	23
WATER RESEARCH	7,913	19
Chemical Engineering Transactions	-	13
CHEMOSPHERE	5,108	11
APPLIED MICROBIOLOGY AND BIOTECHNOLOGY	3,67	10
JOURNAL OF BIOTECHNOLOGY	3,163	10
AQUATIC TOXICOLOGY	3,794	9
ENVIRONMENTAL SCIENCE AND POLLUTION RESEARCH	2,914	9
FRESHWATER BIOLOGY	3,404	8
JOURNAL OF PLANKTON RESEARCH	2,209	8
PHYTOCHEMISTRY	2,905	8
WATER SCIENCE AND TECHNOLOGY	1,624	8

k.

Title of publications	Citations	Date
Microalgae as a raw material for biofuels production	727	2009
Microalgae as substrates for fermentative biogas production in a combined biorefinery concept	332	2010
The impact of nitrogen starvation on the dynamics of triacylglycerol accumulation in nine microalgae strains	259	2012
Nitrogen and phosphorus removal from urban wastewater by the microalga *Scenedesmus obliquus*	250	2000
Protein measurements of microalgal and cyanobacterial biomass	233	2010
Production cost of a real microalgae production plant and strategies to reduce it	218	2012
Competition for light between phytoplankton species: Experimental tests of mechanistic theory	213	1999
Allelopathic growth inhibition of selected phytoplankton species by submerged macrophytes	209	2002
Phytoplankton, not allochthonous carbon, sustains herbivorous zooplankton production	199	2009
Photosynthetic inorganic carbon use by freshwater plants	199	1983
Flocculation of microalgae using cationic starch	193	2010
Determination of limiting polyunsaturated fatty acids in *Daphnia galeata* using a new method to enrich food algae with single fatty acids	187	2002
Removal of nitrogen and phosphorus from wastewater using microalgae immobilized on twin layers: An experimental study	185	2007
Use of chlorophyll fluorescence in metal-stress research: A case study with the green microalga *Scenedesmus*	165	2003
Chemical evidence of kerogen formation in source rocks and oil shales via selective preservation of thin resistant outer walls of microalgae: Origin of ultralaminae	160	1991

S1. Bibliometric overview of the research on *Scenedesmus* sp. in 733 European scientific papers. Main concepts (a), concepts network (b), annual production (c), global collaborations (d), European collaborations (e), annual production by countries (f), main countries (g), main cities (h), emerging concepts (i), main journals (j) and main citations (k).

***Chlamydomonas* sp.**

a.

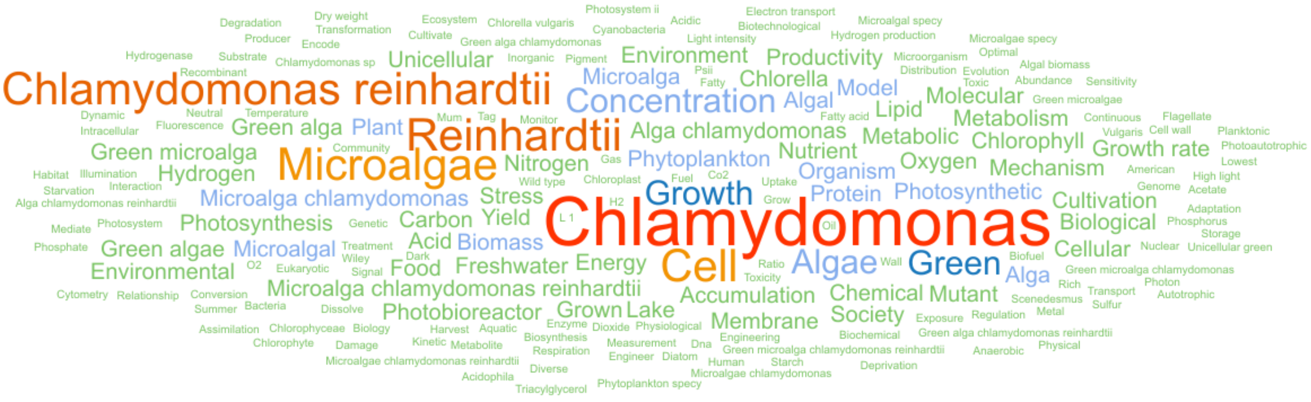

b.

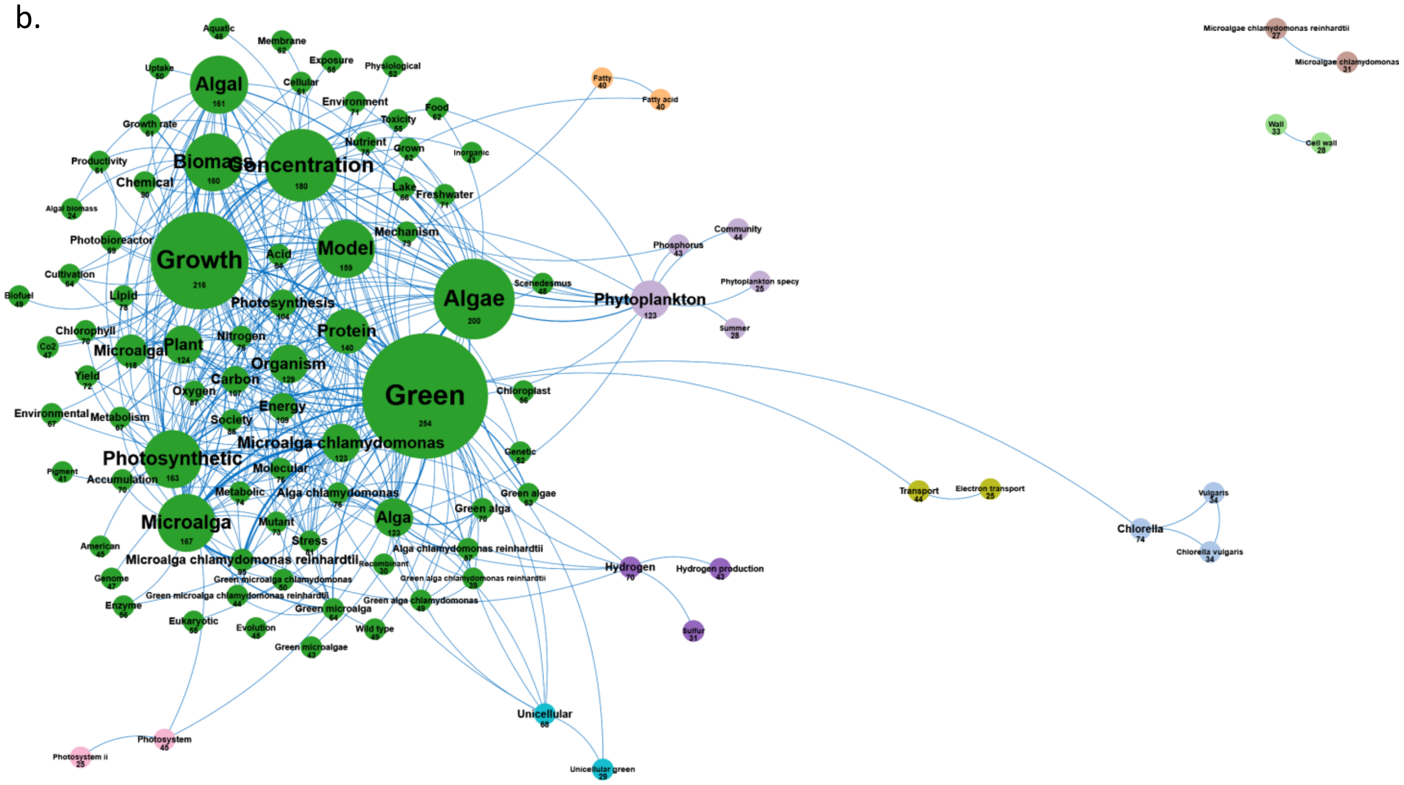

c.

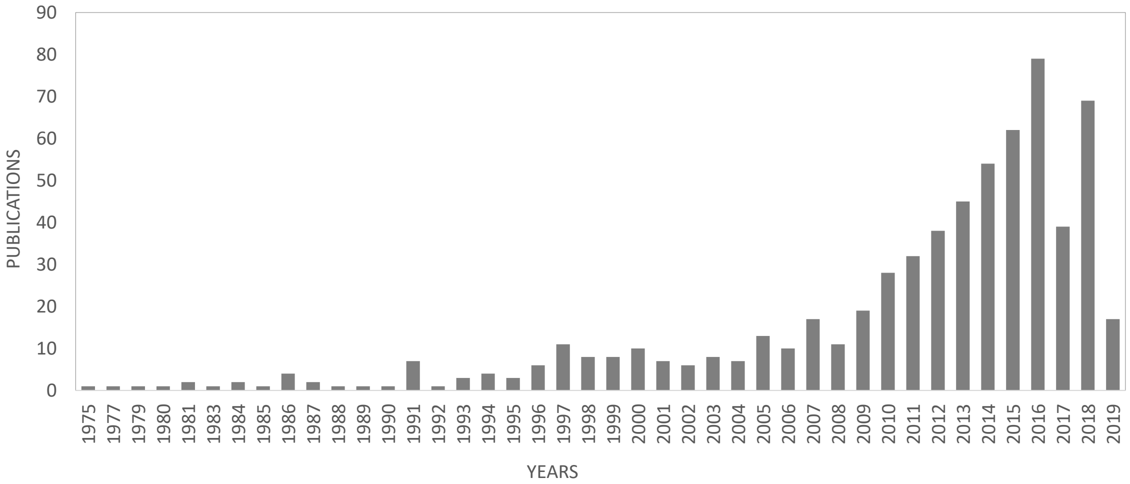

d.

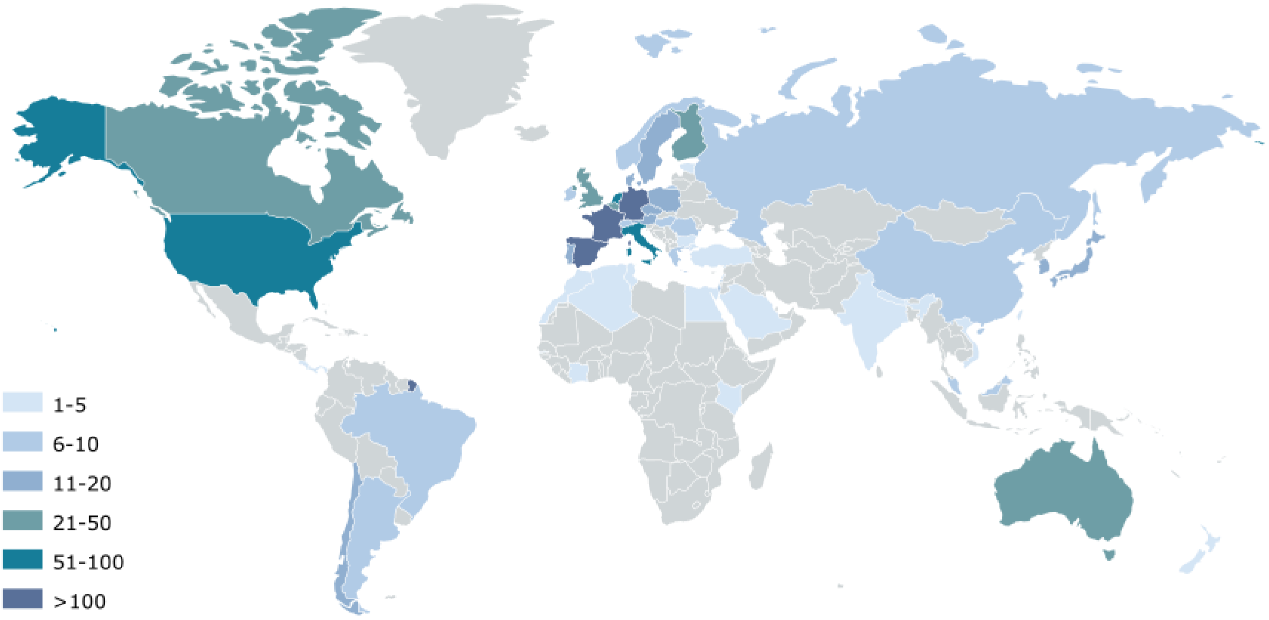

e.

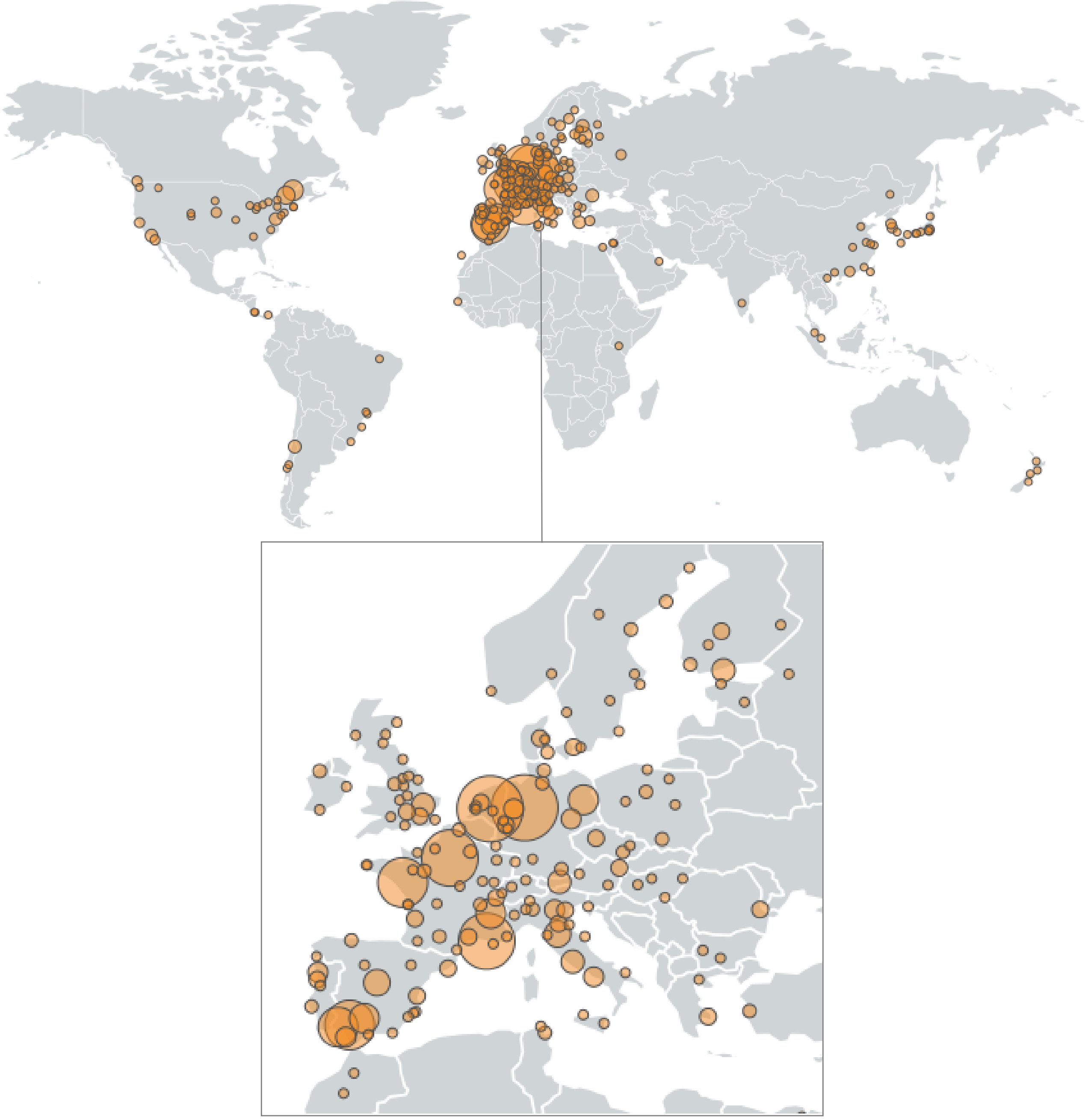

f.

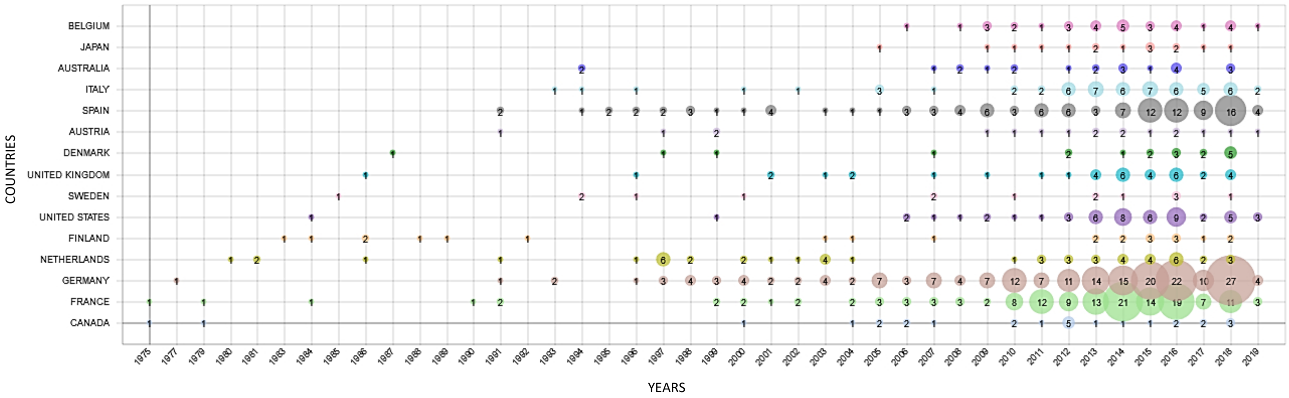

g.

Countries	Publications
GERMANY	199
FRANCE	146
SPAIN	115
ITALY	58
NETHERLANDS	52
UNITED STATES	52
UNITED KINGDOM	37
BELGIUM	33
CANADA	27
FINLAND	23
AUSTRALIA	22
DENMARK	19
AUSTRIA	18
JAPAN	15
SWEDEN	15

h.

Cities	Publications
Bielefeld	41
Wageningen	40
Marseille	34
Paris	33
Nantes	30
Seville	30
Huelva	22
Berlin	16
Grenoble	16
Cordoba	14
Florence	12
Madrid	12
Quebec	12
Montreal	11
Cambridge	10

i.

Emerging concepts	GF
Friendly	5
Springer nature	5
Harbor	4
Hinder	4
Intron	4
Confocal	3
Cyclase	3
Elisa	3
European society	3
Green cell factory	3
Isoprenoid	3
Membrane bioreactor	3
Microalgae population	3
Photobiology	3
Photobiology 2018	3
Proline	3
Rna seq data	3
Surprisal analysis	3
Agar plate	2
Alternative strategy	2

j.

Journals and number of publications	IF	Publications
Algal Research-Biomass Biofuels and Bioproducts	3,723	28
HYDROBIOLOGIA	2,325	22
BIORESOURCE TECHNOLOGY	6,669	21
JOURNAL OF BIOTECHNOLOGY	3,163	19
JOURNAL OF APPLIED PHYCOLOGY	2,635	17
PLANT PHYSIOLOGY	6,305	15
PHOTOSYNTHESIS RESEARCH	3,057	12
AQUATIC TOXICOLOGY	3,794	12
FRESHWATER BIOLOGY	3,404	11
PLoS One	2,776	11
EUROPEAN JOURNAL OF PHYCOLOGY	2,526	10
INTERNATIONAL JOURNAL OF HYDROGEN ENERGY	4,084	9
APPLIED MICROBIOLOGY AND BIOTECHNOLOGY	3,67	9
BIOTECHNOLOGY AND BIOENGINEERING	4,26	9
Scientific Reports	4,011	9

k.

Title of publications	Citations	Date
An outlook on microalgal biofuels	1049	2010
Toxicity of silver nanoparticles to *Chlamydomonas reinhardtii*	965	2008
Oil accumulation in the model green alga *Chlamydomonas reinhardtii*: Characterization, variability between common laboratory strains and relationship with starch reserves	372	2011
The dynamics of photosynthesis	351	2008
Microalgae as substrates for fermentative biogas production in a combined biorefinery concept	332	2010
Chlamydomonas starchless mutant defective in ADP-glucose pyrophosphorylase hyper-accumulates triacylglycerol	202	2010
Altered cell wall morphology in nutrient-deficient phytoplankton and its impact on grazers	183	1997
Potential of industrial biotechnology with cyanobacteria and eukaryotic microalgae	177	2013
Transgenic microalgae as green cell-factories	159	2004
Effective viscosity of microswimmer suspensions	149	2010
A fully predictive model for one-dimensional light attenuation by *Chlamydomonas reinhardtii* in a torus photobioreactor	140	2005
UV-absorbing mycosporine-like compounds in planktonic and benthic organisms from a high-mountain lake	138	1999
Microalgal carbohydrates: An overview of the factors influencing carbohydrates production, and of main bioconversion technologies for production of biofuels	131	2012
Central carbon metabolism and electron transport in *Chlamydomonas reinhardtii*: Metabolic constraints for carbon partitioning between oil and starch	130	2013
The genome of the polar eukaryotic microalga *Coccomyxa subellipsoidea* reveals traits of cold adaptation	130	2012

S2. Bibliometric overview of the research on *Chlamydomonas* sp. in 641 European scientific papers. Main concepts (a), concepts network (b), annual production (c), global collaborations (d), European collaborations (e), annual production by countries (f), main countries (g), main cities (h), emerging concepts (i), main journals (j) and main citations (k).

***Phaeodactylum* sp.**

a.

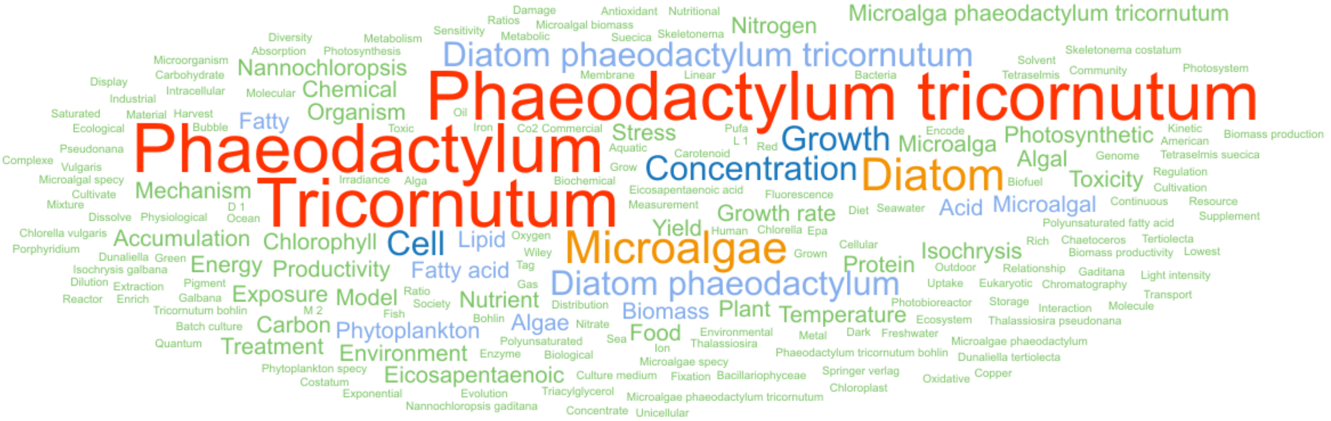

b.

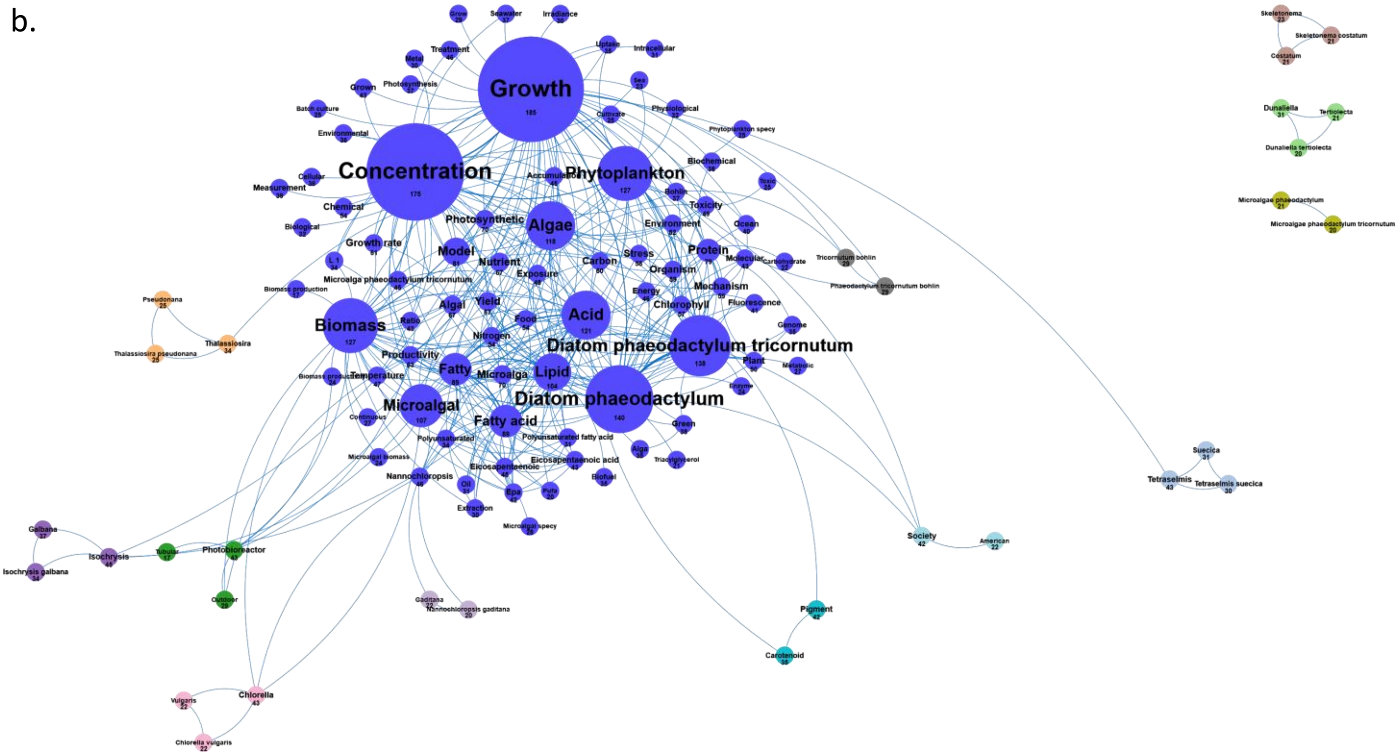

c.

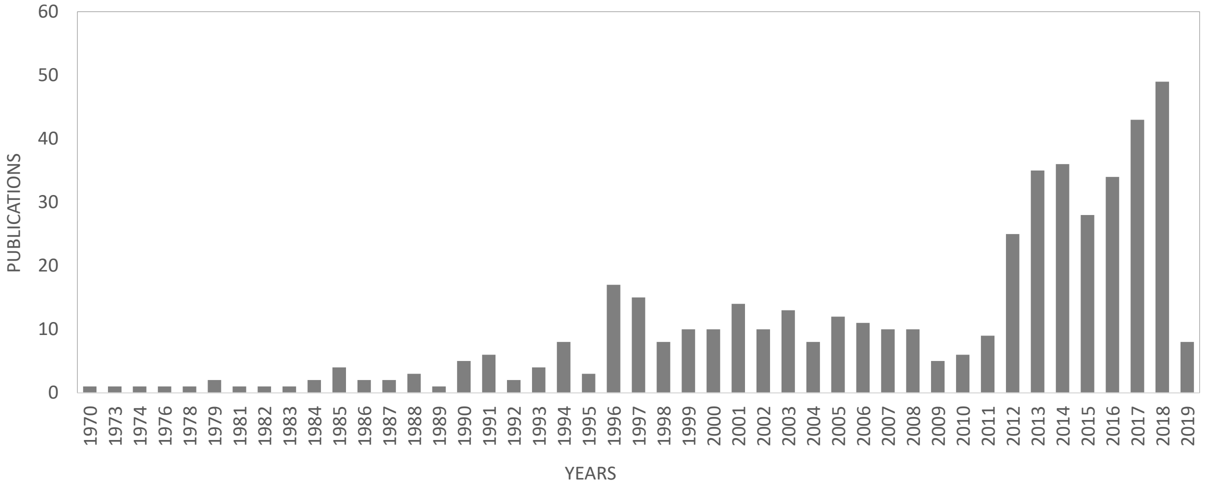

d.

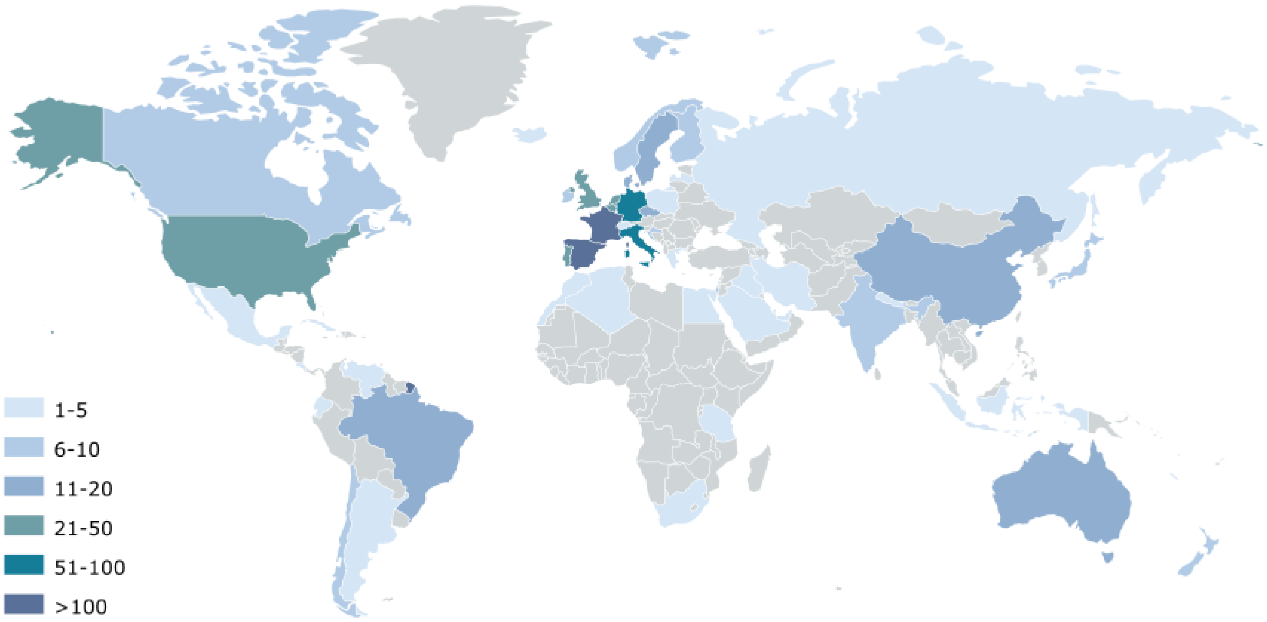

e.

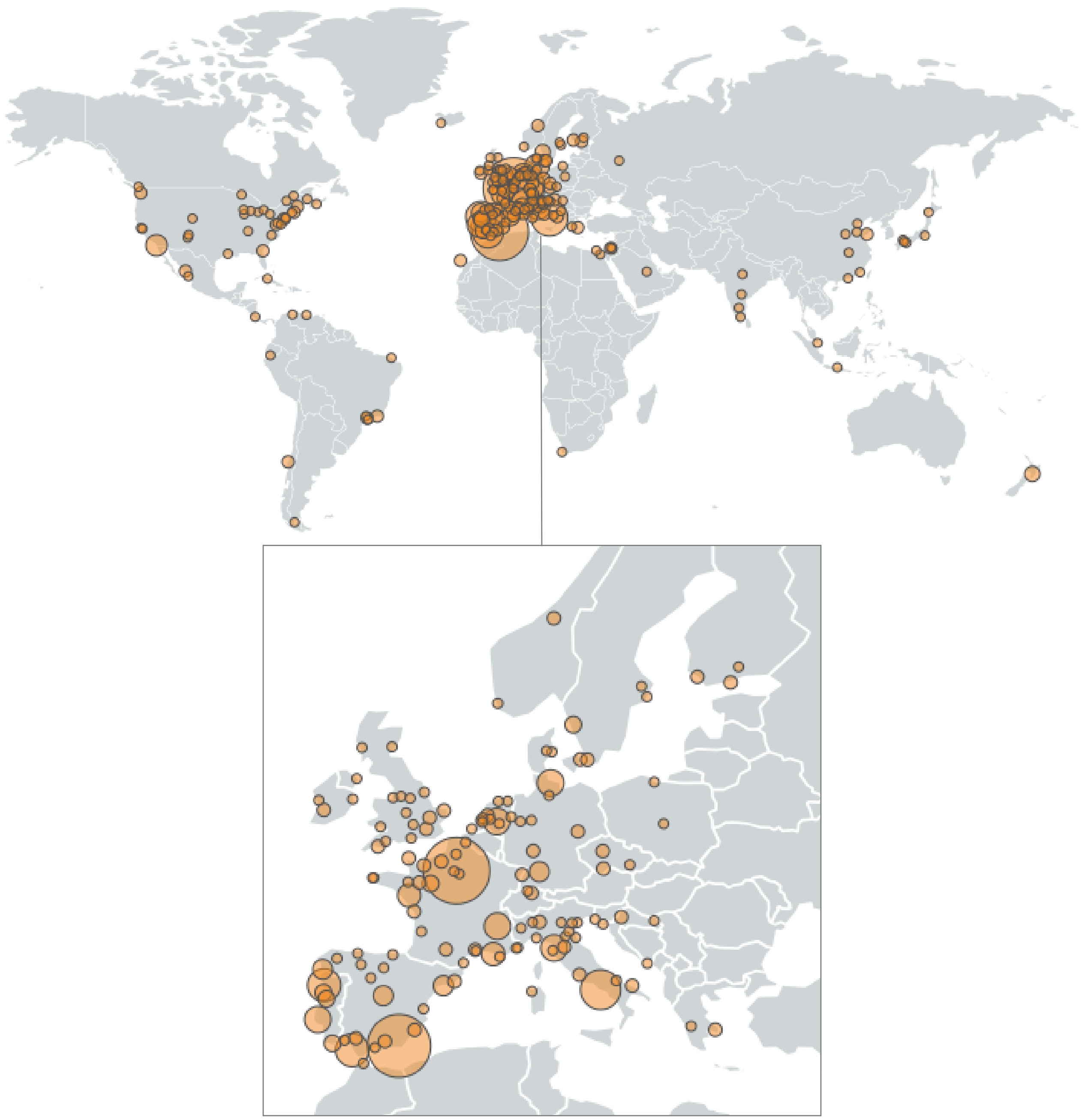

f.

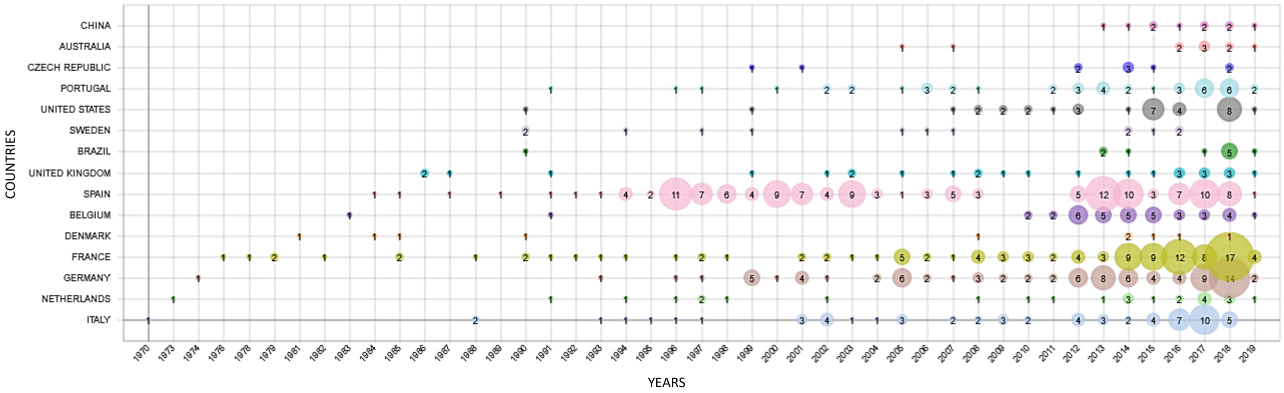

g.

Countries	Publications
SPAIN	141
FRANCE	110
GERMANY	88
ITALY	64
PORTUGAL	44
BELGIUM	38
UNITED STATES	34
UNITED KINGDOM	27
NETHERLANDS	26
SWEDEN	13
BRAZIL	11
AUSTRALIA	10
CHINA	10
CZECH REPUBLIC	10
DENMARK	10

h.

Cities	Publications
Paris	48
Almeria	44
Naples	27
Cadiz	21
Porto	19
Kiel	16
Lisbon	16
Pisa	16
Grenoble	14
Wageningen	14
Marseille	12
Nantes	12
San Diego	11
Stuttgart	9
Tarragone	9

i.

Emerging concepts	GF
Springer nature	7
Edit	5
Genome edit	5
Ph value	5
Cas9	4
Crispr	4
Crispr cas9	4
Effector	4
Mrna	4
Ph 8	4
Bioactivity	3
Delivery	3
Drug	3
Nannochloropsis oceanica	3
Native	3
Overview	3
Volumetric productivity	3
Algal strain	2
Architecture	2
Autofluorescence	2

j.

Journals and number of publications	IF	Publications
JOURNAL OF APPLIED PHYCOLOGY	2,635	22
BIORESOURCE TECHNOLOGY	6,669	20
Algal Research-Biomass Biofuels and Bioproducts	3,723	16
JOURNAL OF PHYCOLOGY	2,831	13
SCIENCE OF THE TOTAL ENVIRONMENT	5,589	11
PLANT PHYSIOLOGY	6,305	10
JOURNAL OF EXPERIMENTAL MARINE BIOLOGY AND ECOLOGY	2,365	10
AQUACULTURE	3,022	10
BIOTECHNOLOGY AND BIOENGINEERING	4,26	8
PLoS One	2,776	8
Marine Drugs	3,772	7
MARINE BIOLOGY	2,134	7
MARINE CHEMISTRY	2,713	6
MARINE ECOLOGY PROGRESS SERIES	2,359	6
MARINE ENVIRONMENTAL RESEARCH	3,445	6

k.

Title of publications	Citations	Date
Recovery of microalgal biomass and metabolites: Process options and economics	1146	2003
An outlook on microalgal biofuels	1049	2010
Tubular photobioreactor design for algal cultures	336	2001
Feeding and digestion by the mussel *Mytilus edulis* L. (Bivalvia: Mollusca) in mixtures of silt and algal cells at low concentrations	246	1987
Whole-cell response of the pennate diatom *Phaeodactylum tricornutum* to iron starvation	222	2008
Effects of growth rate, CO2 concentration, and cell size on the stable carbon isotope fractionation in marine phytoplankton	197	1999
Flocculation of microalgae using cationic starch	193	2010
Biomass production and variation in the biochemical profile (total protein, carbohydrates, RNA, lipids and fatty acids) of seven species of marine microalgae	190	1989
Coagulation efficiency and aggregate formation in marine phytoplankton	187	1990
Influence of the diadinoxanthin pool size on photoprotection in the marine planktonic diatom *Phaeodactylum tricornutum*	182	2002
Gene silencing in the marine diatom *Phaeodactylum tricornutum*	180	2009
Airlift-driven external-loop tubular photobioreactors for outdoor production of microalgae: Assessment of design and performance	173	2001
A model for light distribution and average solar irradiance inside outdoor tubular photobioreactors for the microalgal mass culture	167	1997
Algae displaying the diadinoxanthin cycle also possess the violaxanthin cycle	162	1999
The application of micro-FTIR spectroscopy to analyze nutrient stress-related changes in biomass composition of phytoplankton algae	161	2005

S3. Bibliometric overview of the research on *Phaeodactylum* sp. in 478 European scientific papers. Main concepts (a), concepts network (b), annual production (c), global collaborations (d), European collaborations (e), annual production by countries (f), main countries (g), main cities (h), emerging concepts (i), main journals (j) and main citations (k).

Nannochloropsis sp.

a.

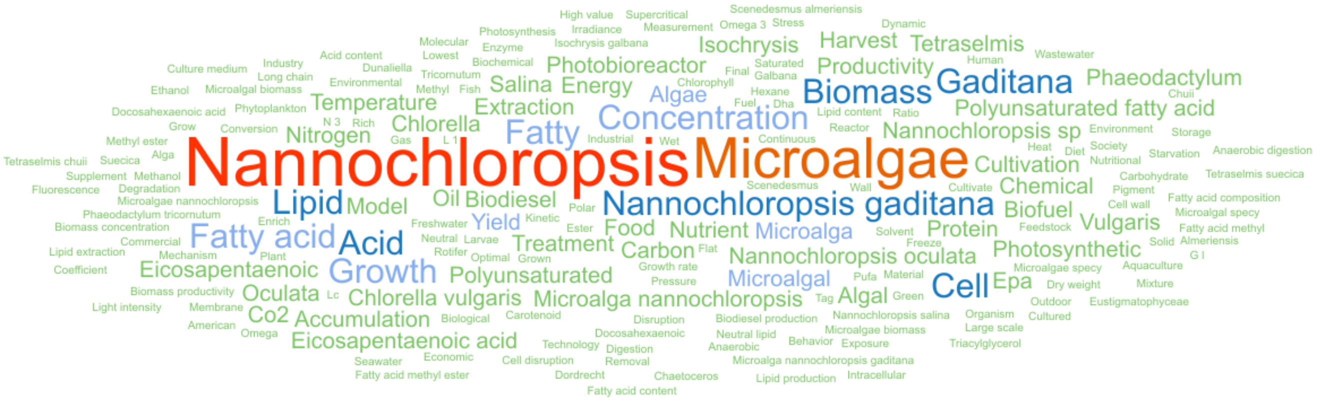

b.

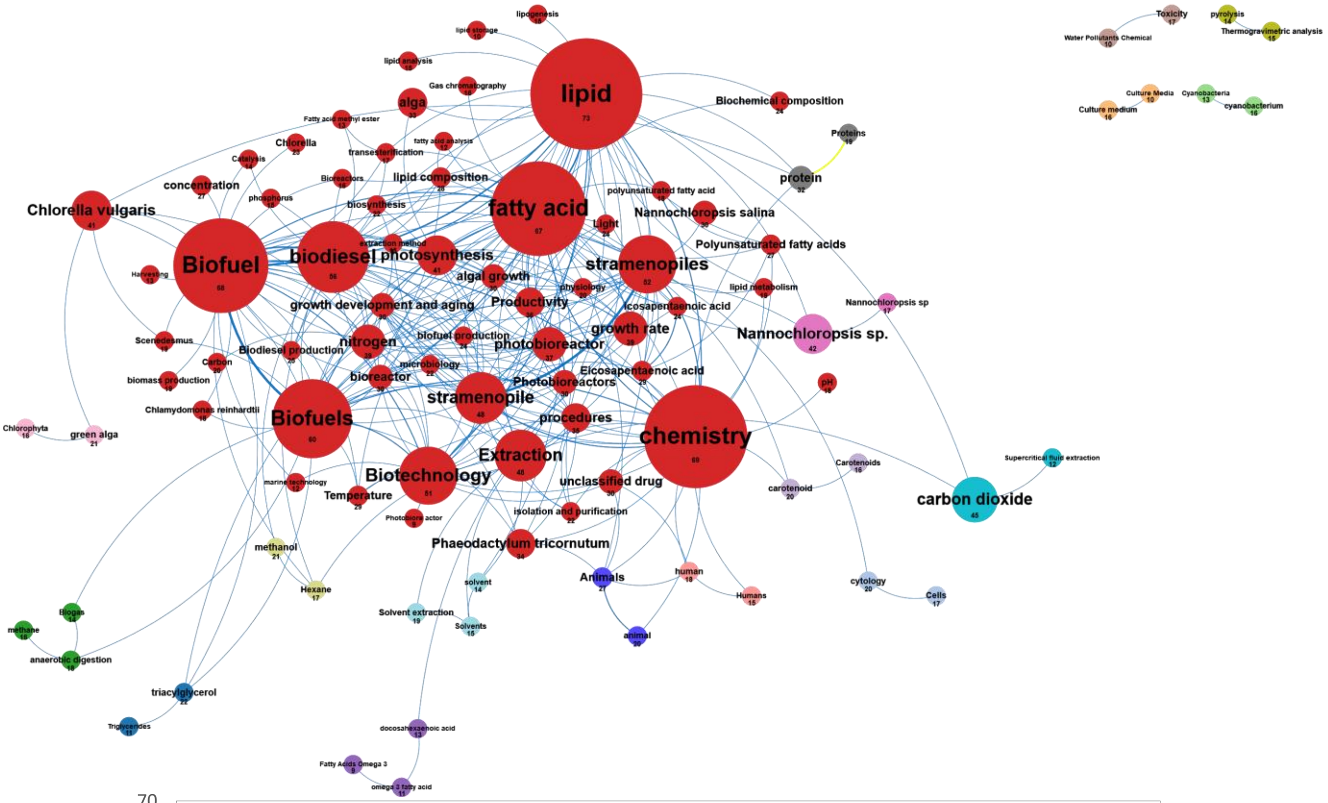

c.

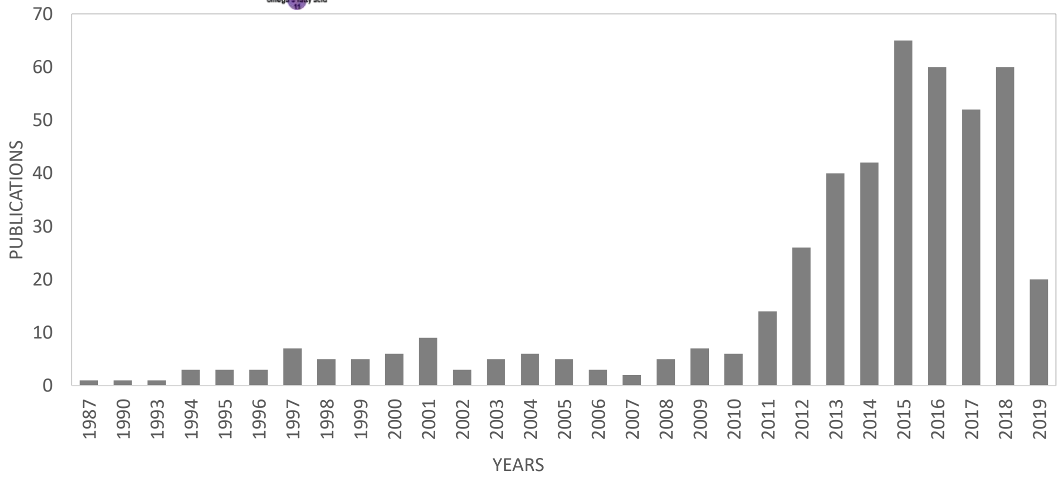

d.

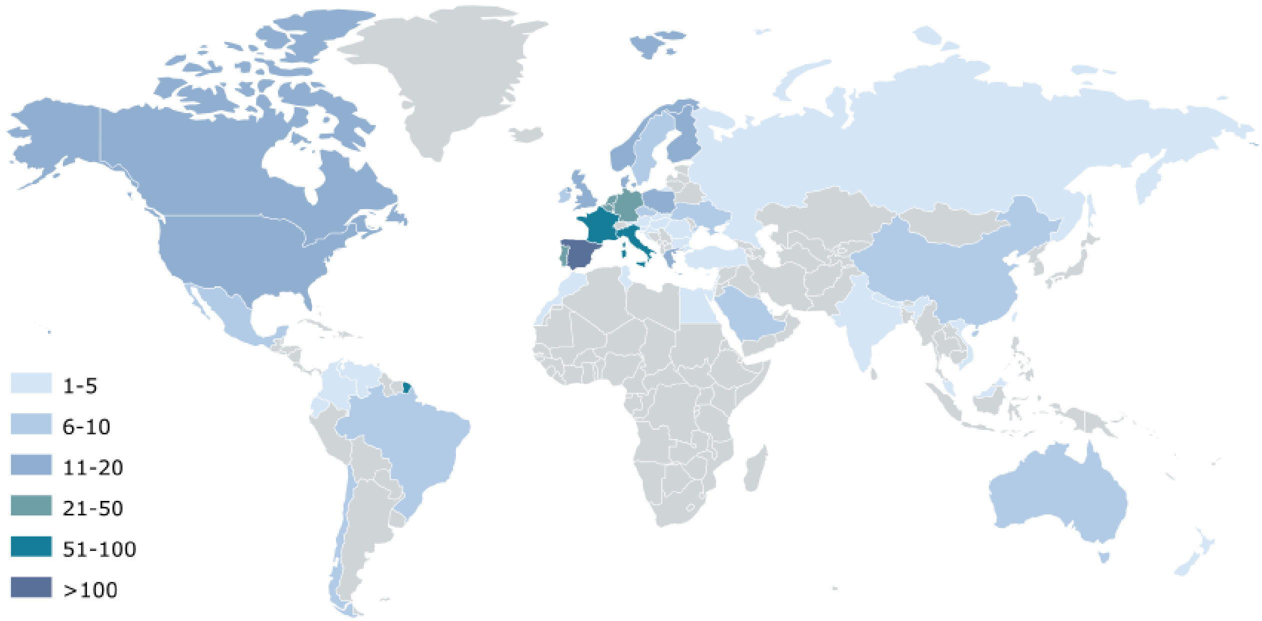

e.

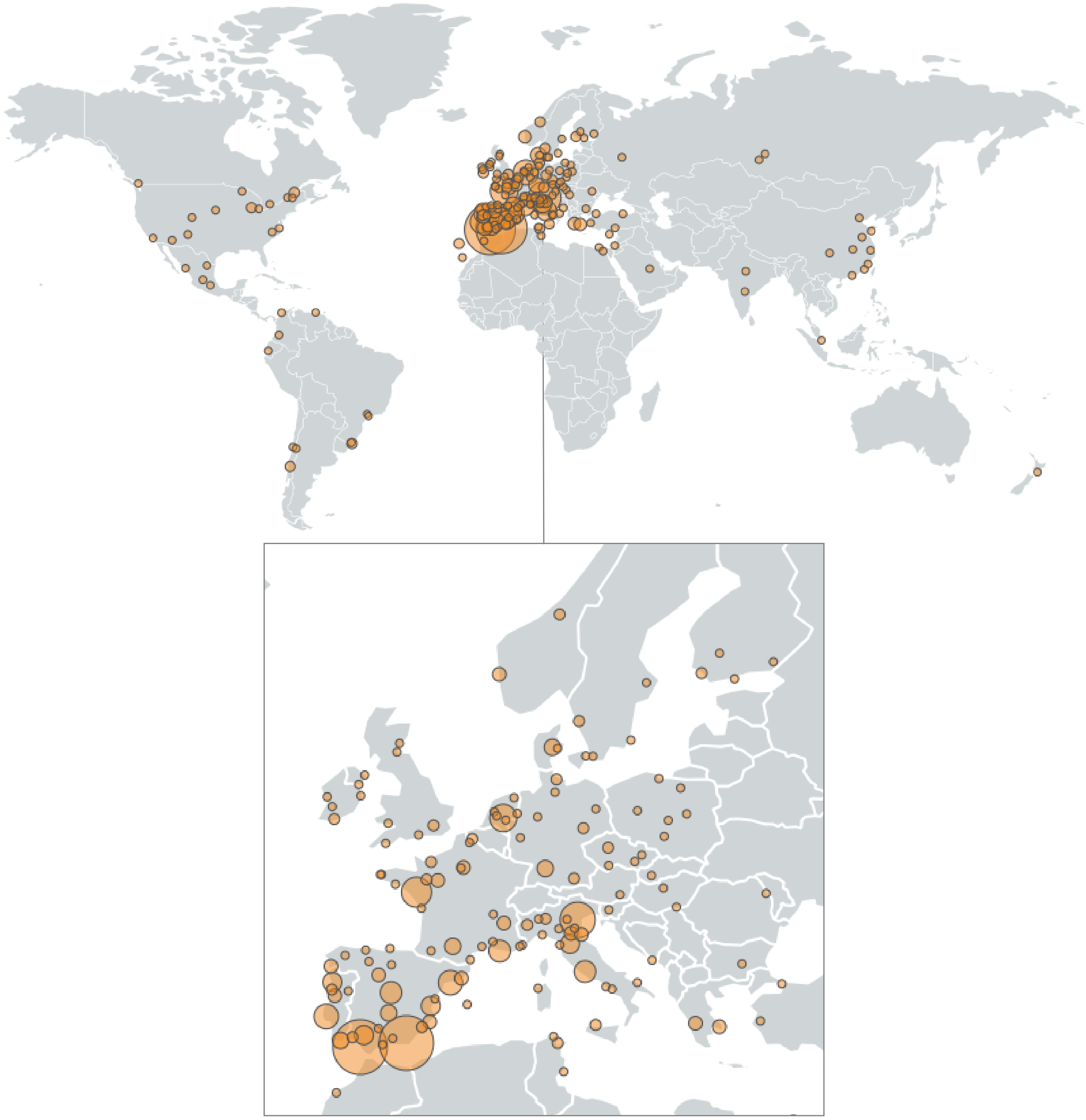

f.

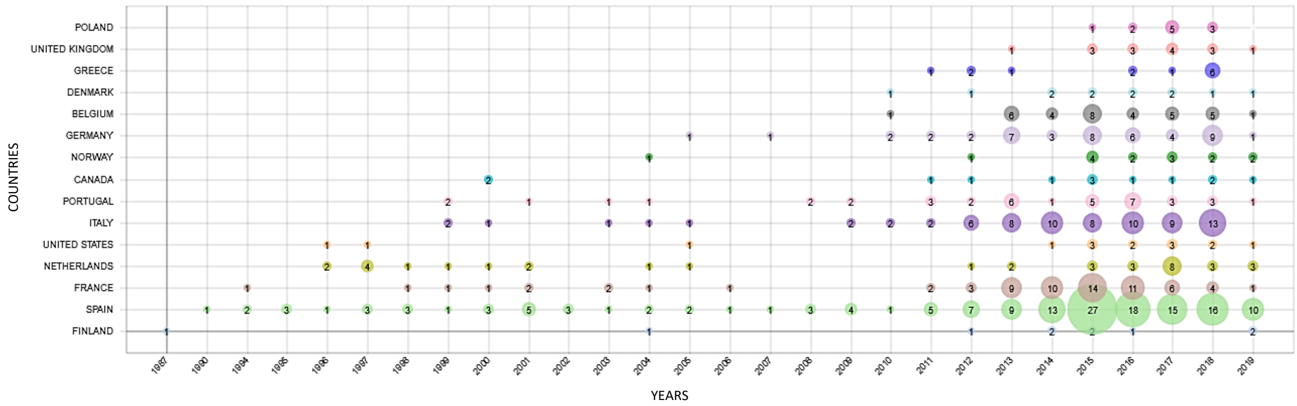

g.

Countries	Publications
SPAIN	160
ITALY	76
FRANCE	70
GERMANY	46
PORTUGAL	40
NETHERLANDS	36
BELGIUM	34
NORWAY	15
UNITED KINGDOM	15
UNITED STATES	15
CANADA	13
GREECE	13
DENMARK	12
POLAND	11
FINLAND	10

h.

Cities	Publications
Cadiz	42
Almeria	41
Padova	26
Nantes	21
Wageningen	19
Lisbon	16
Tarragone	15
Rome	14
Marseille	13
Madrid	12
Florence	11
Porto	11
Seville	10
Valencia	10
Stuttgart	9

i.

Emerging concepts	GF
Springer nature	8
Switzerland	5
Basel	4
Farm	4
John	4
John wiley	4
Maintenance	4
Son	4
Bioavailability	3
Coal	3
Economy	3
Fishery	3
Food chain	3
Gamma	3
Human consumption	3
Nan	3
Nannochloropsis oceanica ccmp1779	3
Oceanica ccmp1779	3
Render	3
Separately	3

j.

Journals and number of publications	IF	Publications
BIORESOURCE TECHNOLOGY	6,669	62
Algal Research-Biomass Biofuels and Bioproducts	3,723	47
JOURNAL OF APPLIED PHYCOLOGY	2,635	25
AQUACULTURE	3,022	14
Marine Drugs	3,772	10
FOOD CHEMISTRY	5,399	8
ORGANIC GEOCHEMISTRY	3,12	7
Biotechnology for Biofuels	5,452	7
Chemical Engineering Transactions	-	7
PLoS One	2,776	6
PHYTOCHEMISTRY	2,905	6
HYDROBIOLOGIA	2,325	6
JOURNAL OF AGRICULTURAL AND FOOD CHEMISTRY	3,571	6
FUEL	5,128	5
AQUACULTURE RESEARCH	1,502	5

k.

Title of publications	Citations	Date
Microalgae for oil: Strain selection, induction of lipid synthesis and outdoor mass cultivation in a low-cost photobioreactor	1585	2009
Microalgae as a raw material for biofuels production	727	2009
Effect of temperature and nitrogen concentration on the growth and lipid content of *Nannochloropsis oculata* and *Chlorella vulgaris* for biodiesel production	647	2009
Flocculation of microalgae using cationic starch	193	2010
Thermogravimetric-mass spectrometric analysis of lignocellulosic and marine biomass pyrolysis	165	2012
Cellular DNA content of marine phytoplankton using two new fluorochromes: Taxonomic and ecological implications	163	1997
Distribution of aliphatic, nonhydrolyzable biopolymers in marine microalgae	157	1999
The response of *Nannochloropsis gaditana* to nitrogen starvation includes de novo biosynthesis of triacylglycerols, a decrease of chloroplast galactolipids, and reorganization of the photosynthetic apparatus	156	2013
Production of eicosapentaenoic acid by *Nannochloropsis* sp. cultures in outdoor tubular photobioreactors	149	1999
Oil production by the marine microalgae *Nannochloropsis* sp. F&M-M24 and *Tetraselmis suecica* F&M-M33	130	2012
Adjusted light and dark cycles can optimize photosynthetic efficiency in algae growing in photobioreactors	124	2012
Solvent-free' ultrasound-assisted extraction of lipids from fresh microalgae cells: A green, clean and scalable process	123	2012
Resistant biomacromolecules in marine microalgae of the classes eustigmatophyceae and chlorophyceae: Geochemical implications	123	1997
Pyrolysis, combustion and gasification characteristics of *Nannochloropsis gaditana* microalgae	120	2013
Supercritical fluid extraction of carotenoids and chlorophyll a from *Nannochloropsis gaditana*	116	2005

S4. Bibliometric overview of the research on *Nannochloropsis* sp. in 465 European scientific papers. Main concepts (a), concepts network (b), annual production (c), global collaborations (d), European collaborations (e), annual production by countries (f), main countries (g), main cities (h), emerging concepts (i), main journals (j) and main citations (k).

***Dunaliella* sp.**

a.

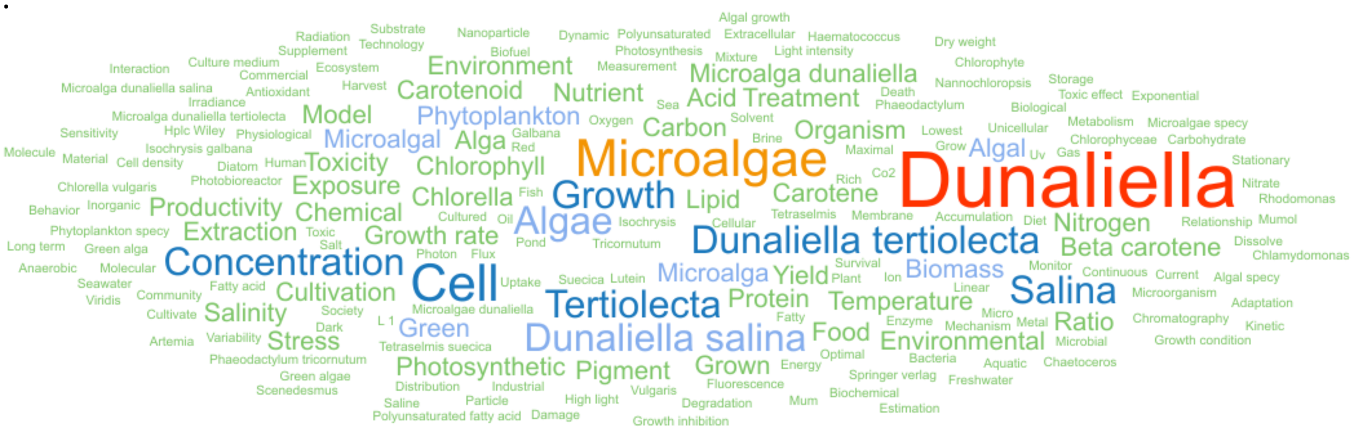

b.

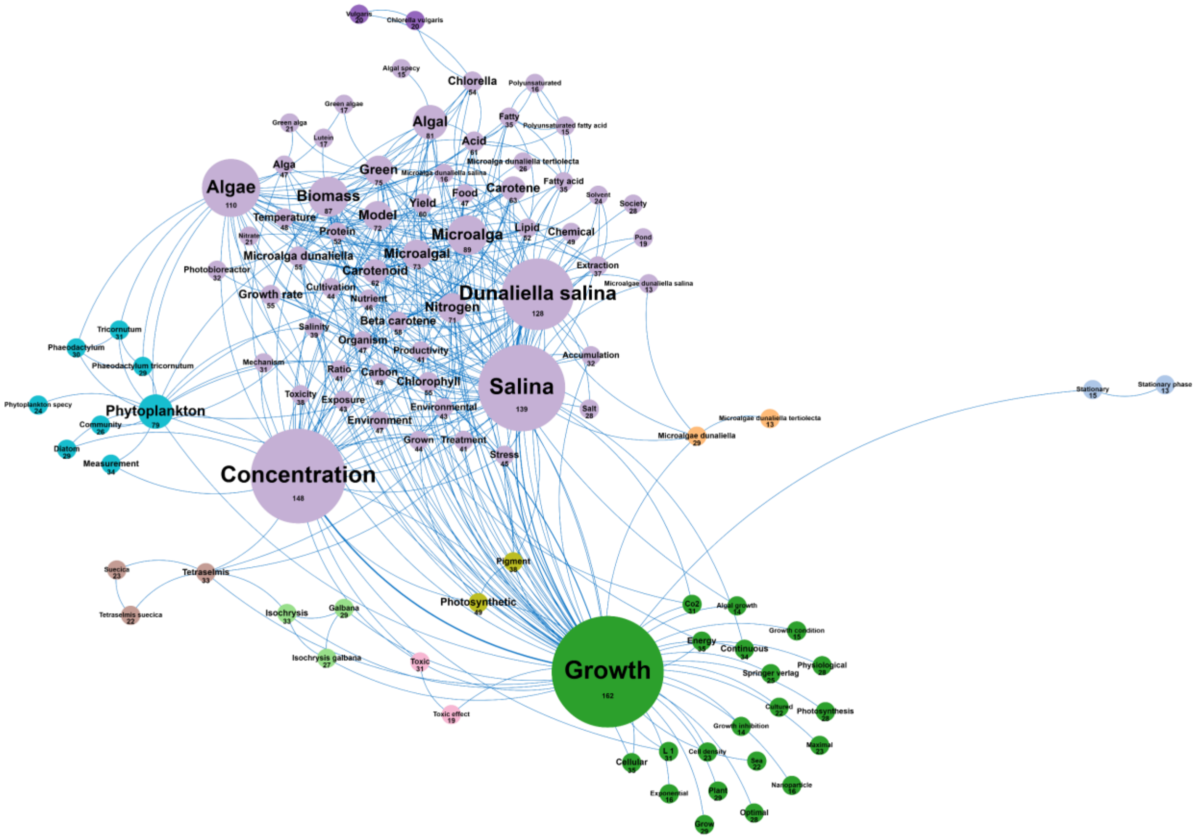

c.

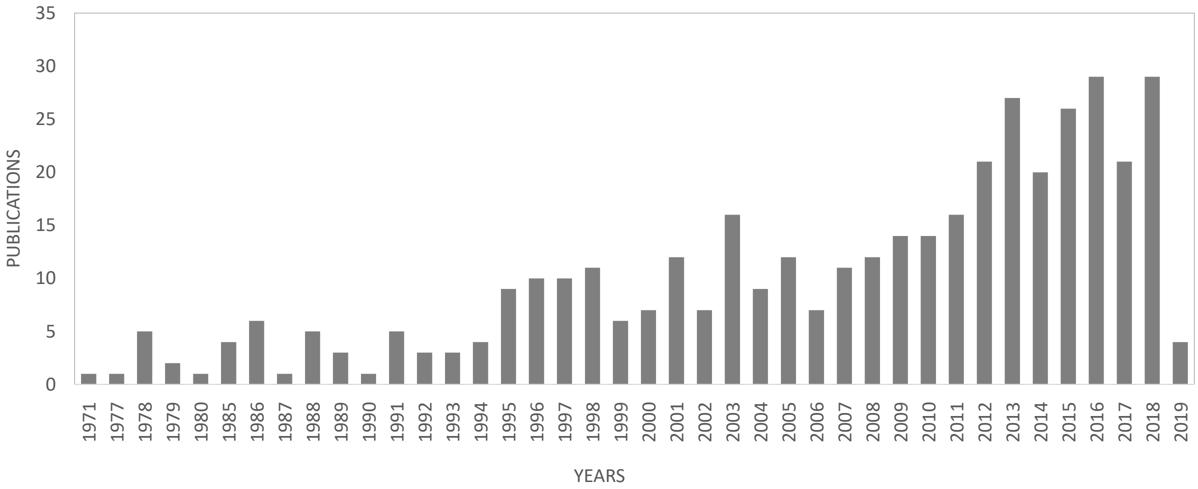

d.

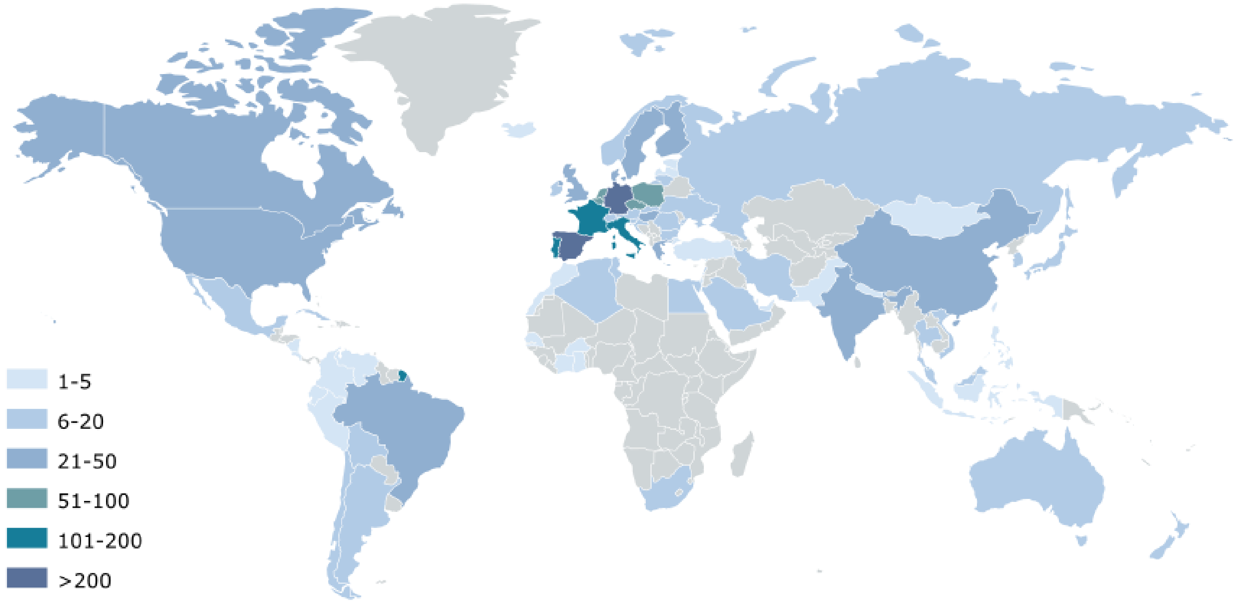

e.

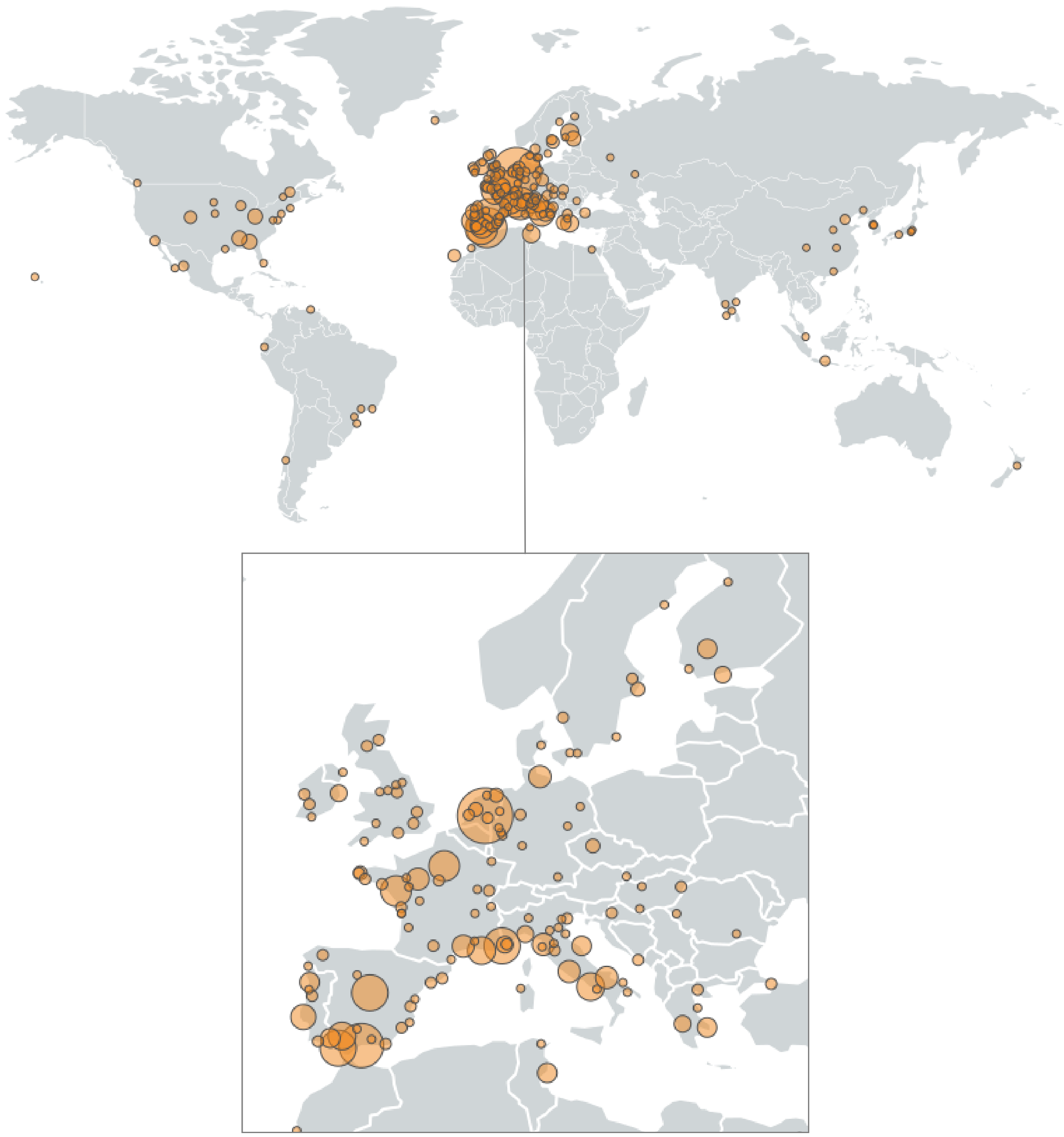

f.

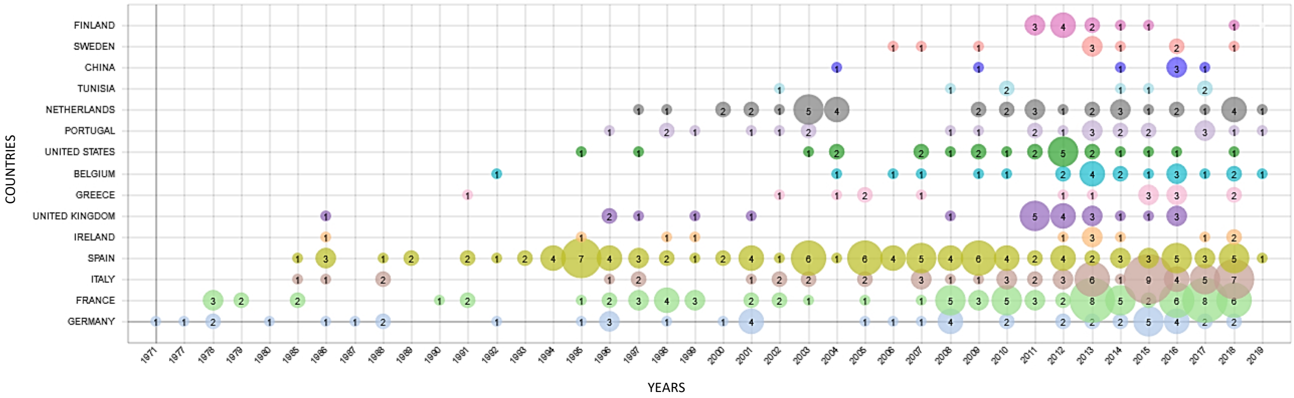

g.

Countries	Publications
SPAIN	104
FRANCE	83
ITALY	59
GERMANY	48
NETHERLANDS	38
PORTUGAL	25
UNITED KINGDOM	24
UNITED STATES	24
BELGIUM	22
GREECE	16
FINLAND	12
IRELAND	12
SWEDEN	10
TUNISIA	8
CHINA	7

h.

Cities	Publications
Wageningen	26
Malaga	20
Cadiz	16
Madrid	15
Sophia Antipolis	15
Nantes	13
Paris	13
Marseille	12
Naples	12
Seville	12
Lisbon	10
Kiel	9
Pisa	9
Foggia	8
Le Mans	8

i.

Emerging concepts	GF
Ag	3
Membrane filtration	3
Pilot scale	3
Raceway	3
Aff	2
Algal pond	2
Algal productivity	2
Algal productivity model	2
Bioactivity	2
Biomass concentration	2
Cell disruption	2
Cheaper	2
Combine diet	2
Dry sample	2
Dynamic filtration	2
Elongase	2
Eventually	2
Explosion	2
Final concentration	2
Food supplement	2

j.

Journals and number of publications	IF	Publications
BIORESOURCE TECHNOLOGY	6,669	25
JOURNAL OF APPLIED PHYCOLOGY	2,635	22
AQUACULTURE	3,022	11
Algal Research-Biomass Biofuels and Bioproducts	3,723	11
BIOTECHNOLOGY AND BIOENGINEERING	4,26	9
MARINE BIOLOGY	2,134	8
Marine Drugs	3,772	8
SCIENCE OF THE TOTAL ENVIRONMENT	5,589	7
APPLIED MICROBIOLOGY AND BIOTECHNOLOGY	3,67	7
AQUATIC TOXICOLOGY	3,794	7
JOURNAL OF EXPERIMENTAL MARINE BIOLOGY AND ECOLOGY	2,365	7
EUROPEAN JOURNAL OF PHYCOLOGY	2,526	7
JOURNAL OF BIOTECHNOLOGY	3,163	6
BIOMOLECULAR ENGINEERING	-	6
LIMNOLOGY AND OCEANOGRAPHY	4,325	6

k.

Title of publications	Citations	Date
Microalgae as a raw material for biofuels production	727	2009
Outdoor cultivation of microalgae for carotenoid production: Current state and perspectives	355	2007
Microalgae as substrates for fermentative biogas production in a combined biorefinery concept	332	2010
Microorganisms and microalgae as sources of pigments for food use: A scientific oddity or an industrial reality?	280	2005
Supercritical carbon dioxide extraction of compounds with pharmaceutical importance from microalgae	271	2003
Potential carbon dioxide fixation by industrially important microalgae	239	2010
Transport and degradation of phytoplankton in permeable sediment	229	2000
Carotenoid content of chlorophycean microalgae: factors determining lutein accumulation in *Muriellopsis* sp. (Chlorophyta)	157	2000
Inhibitory effects of silver nanoparticles in two green algae, *Chlorella vulgaris* and *Dunaliella tertiolecta*	156	2012
Production of *Dunaliella salina* biomass rich in 9-cis-beta-carotene and lutein in a closed tubular photobioreactor	141	2005
Dissolved esterase activity as a tracer of phytoplankton lysis: Evidence of high phytoplankton lysis rates in the northwestern Mediterranean	141	1998
Comparison of supercritical fluid and ultrasound-assisted extraction of carotenoids and chlorophyll a from *Dunaliella salina*	127	2009
Study on the microalgal pigments extraction process: Performance of microwave assisted extraction	123	2011
Milking of microalgae	114	2004
Cell fragility - The key problem of microalgae mass production in closed photobioreactors	114	1991

S5. Bibliometric overview of the research on *Dunaliella* sp. in 405 European scientific papers. Main concepts (a), concepts network (b), annual production (c), global collaborations (d), European collaborations (e), annual production by countries (f), main countries (g), main cities (h), emerging concepts (i), main journals (j) and main citations (k).

Isochrysis **sp.**

a.

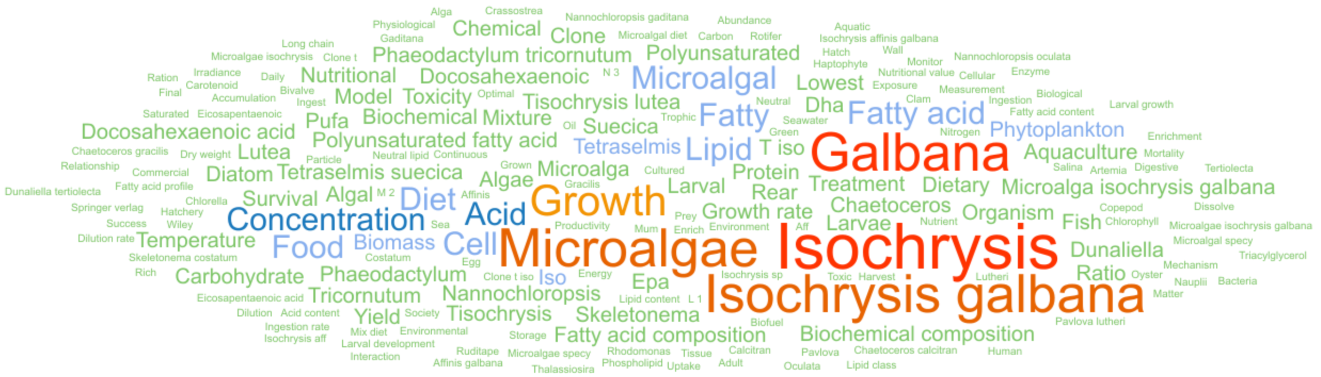

b.

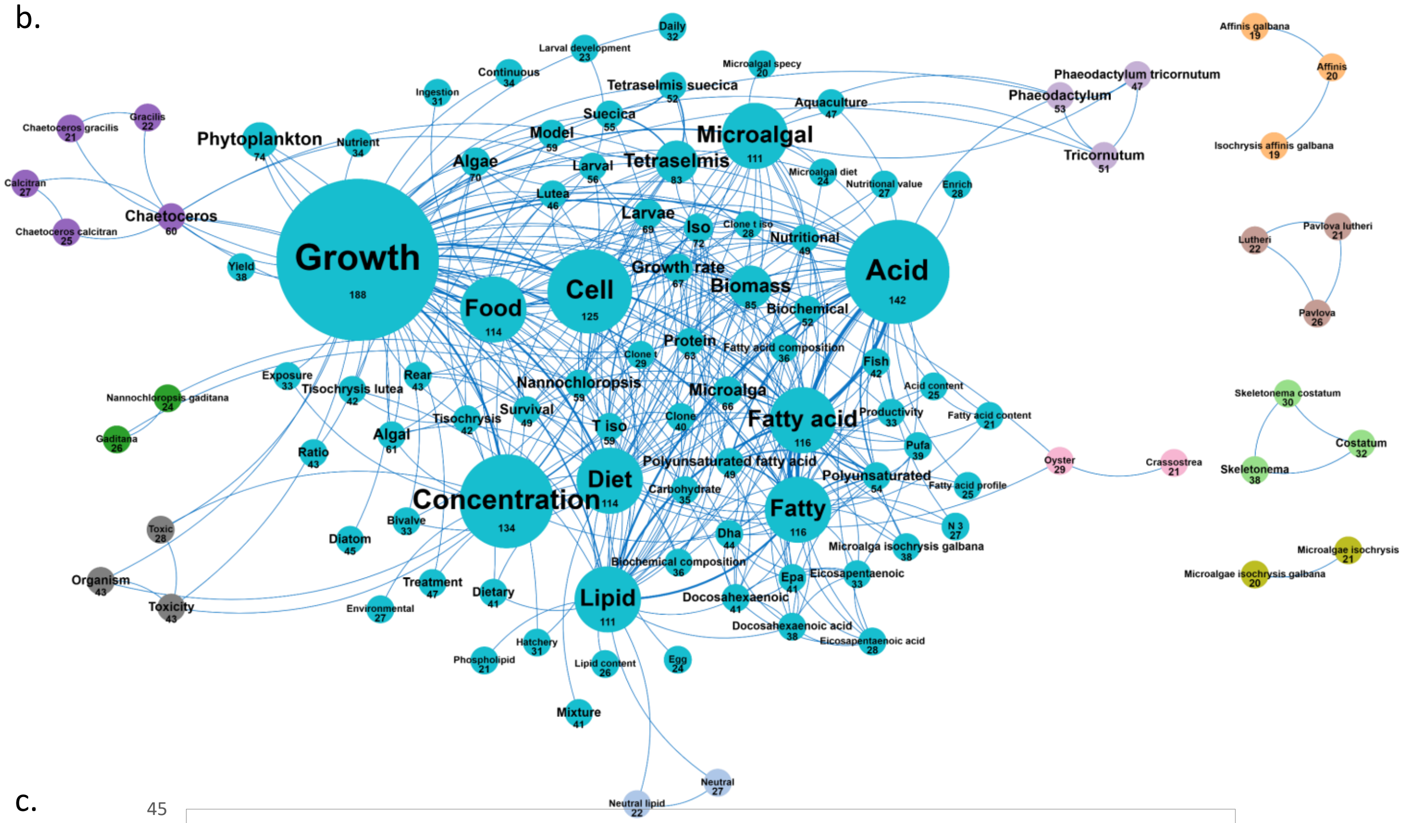

c.

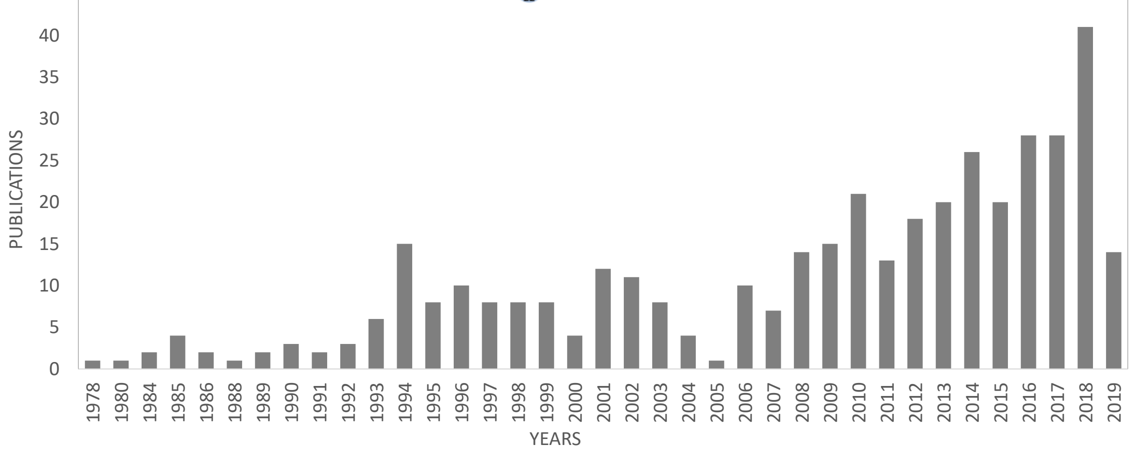

d.

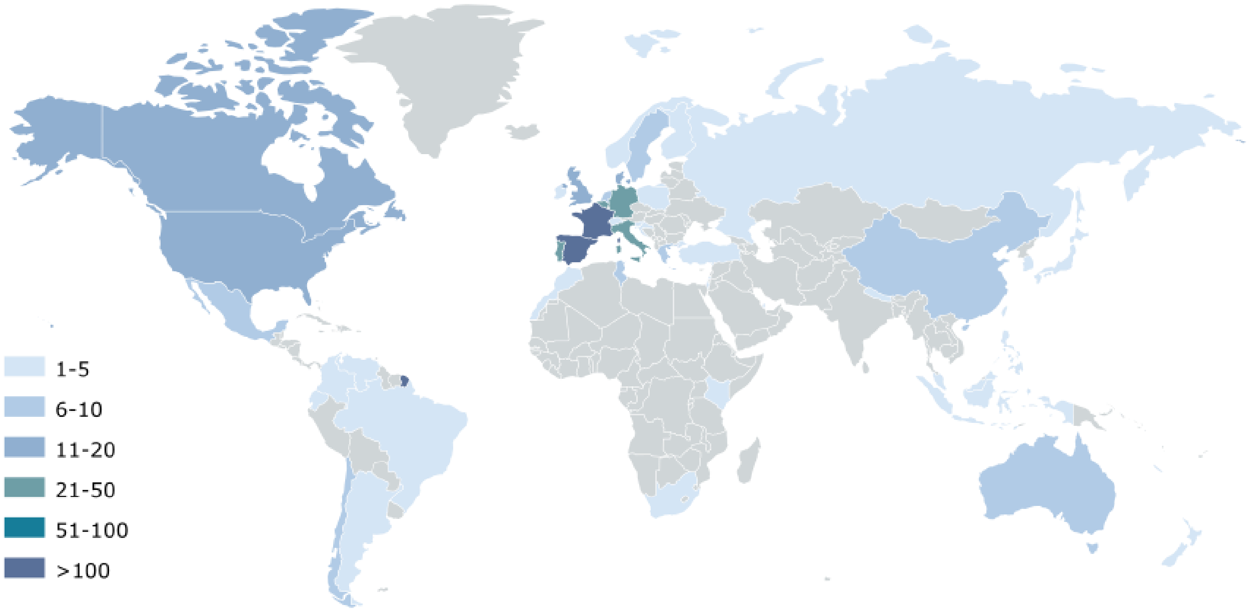

e.

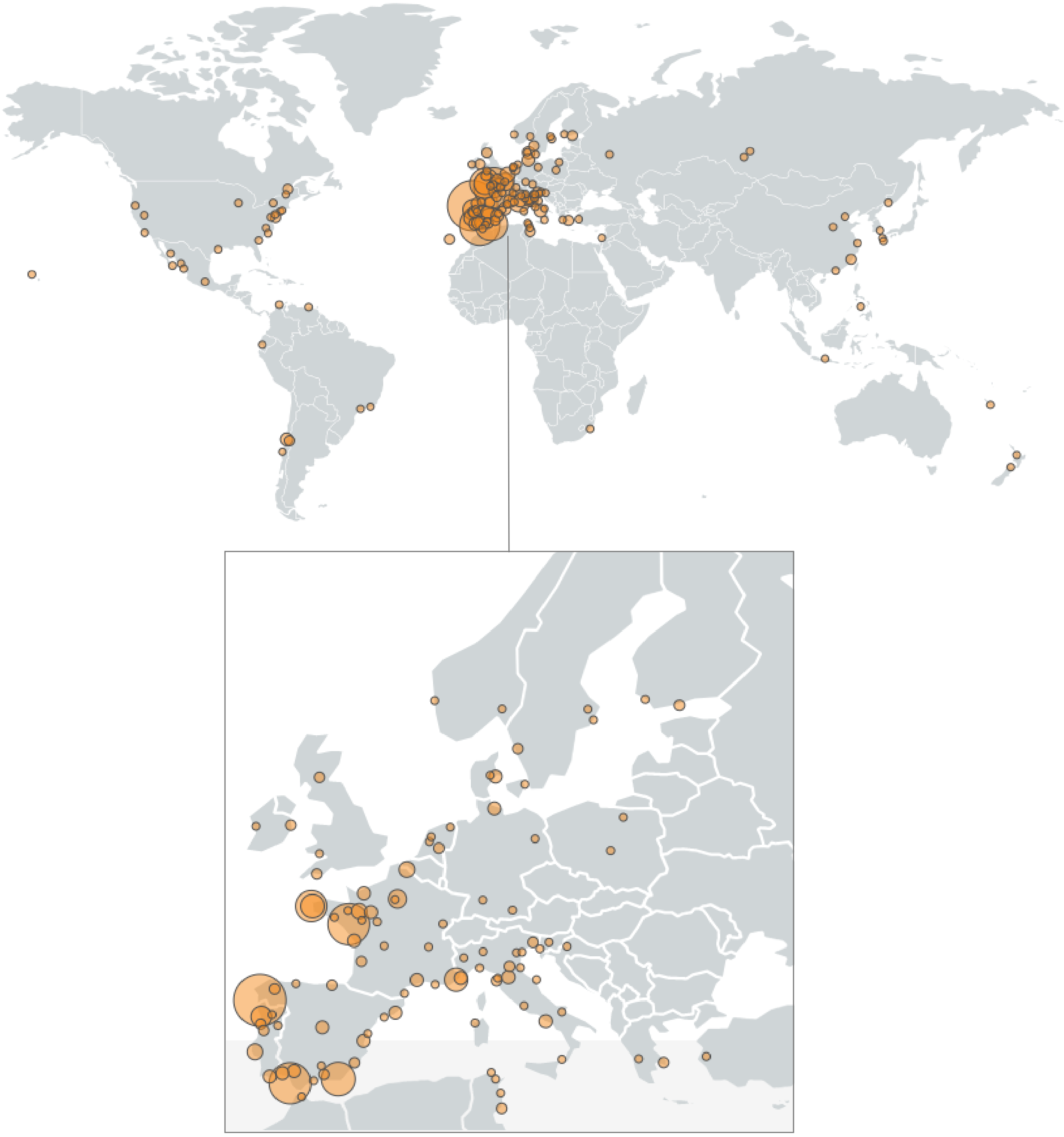

f.

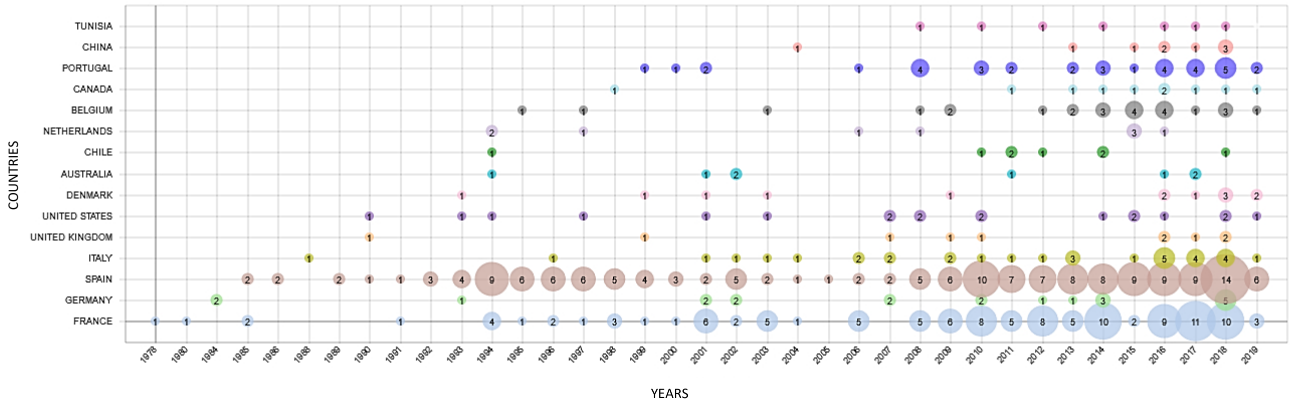

g.

Countries	Publications
SPAIN	167
FRANCE	119
PORTUGAL	35
ITALY	33
BELGIUM	25
GERMANY	21
UNITED STATES	19
DENMARK	13
CANADA	10
UNITED KINGDOM	10
CHINA	9
NETHERLANDS	9
AUSTRALIA	8
CHILE	8
TUNISIA	7

h.

Cities	Publications
Vigo	43
Nantes	34
Cadiz	33
Almeria	26
Plouzane	22
Brest	17
Sophia Antipolis	15
Porto	13
Paris	10
Laval	9
Lisbon	9
Lille	8
Barcelona	7
Le Mans	7
Faro	6

i.

Emerging concepts	GF
Springer nature	10
Almeriensis	4
Blend	4
Isolipidic	4
Microalga tisochrysis lutea	4
Aminopeptidase	3
Anti inflammatory	3
Functionality	3
Germany	3
Oxidative stress	3
Pesticide	3
Potential effect	3
Protease	3
Scale production	3
Scenedesmus almeriensis	3
Senegalensis	3
Senegalese	3
Solea	3
Solea senegalensis	3
Springer verlag gmbh	3

j.

Journals and number of publications	IF	Publications
AQUACULTURE	3,022	52
JOURNAL OF APPLIED PHYCOLOGY	2,635	27
Algal Research-Biomass Biofuels and Bioproducts	3,723	19
AQUACULTURE RESEARCH	1,502	15
JOURNAL OF EXPERIMENTAL MARINE BIOLOGY AND ECOLOGY	2,365	13
MARINE BIOLOGY	2,134	11
AQUACULTURE INTERNATIONAL	1,455	9
MARINE ECOLOGY PROGRESS SERIES	2,359	8
CHEMOSPHERE	5,108	7
ECOTOXICOLOGY AND ENVIRONMENTAL SAFETY	4,527	7
JOURNAL OF PHYCOLOGY	2,831	7
JOURNAL OF PLANKTON RESEARCH	2,209	6
BIORESOURCE TECHNOLOGY	6,669	6
PHYTOCHEMISTRY	2,905	6
SCIENCE OF THE TOTAL ENVIRONMENT	5,589	5

k.

Title of publications	Citations	Date
Biomass production and variation in the biochemical profile (total protein, carbohydrates, RNA, lipids and fatty acids) of seven species of marine microalgae	190	1989
Effects of nitrogen source and growth phase on proximate biochemical composition, lipid classes and fatty acid profile of the marine microalga *Isochrysis galbana*	160	1998
Antioxidant potential of microalgae in relation to their phenolic and carotenoid content	119	2012
Effects of temperature on photosynthetic parameters and TEP production in eight species of marine microalgae	107	2008
Comparison between extraction of lipids and fatty acids from microalgal biomass	106	1994
Determination of DPPH radical oxidation caused by methanolic extracts of some microalgal species by linear regression analysis of spectrophotometric measurements	103	2007
A study on simultaneous photolimitation and photoinhibition in dense microalgal cultures taking into account incident and averaged irradiances	102	1996
Comparison of microalgal biomass profiles as novel functional ingredient for food products	95	2013
Modelling neutral lipid production by the microalga *Isochrysis* aff. *galbana* under nitrogen limitation	95	2011
Influence of cellular density on determination of EC50 in microalgal growth inhibition tests	94	2000
Influence of phytoplankton diet mixtures on microalgae consumption, larval development and settlement of the Pacific oyster *Crassostrea gigas* (Thunberg)	91	2006
Toxicity and bioaccumulation of copper and lead in five marine microalgae	88	2009
Iron-mediated effects on nitrate reductase in marine phytoplankton	85	1994
Estimation of oxygen evolution by marine phytoplankton from measurement of the efficiency of Photosystem II electron flow	83	1997
Concentration and purification of stearidonic, eicosapentaenoic, and docosahexaenoic acids from cod liver oil and the marine microalga *Isochrysis galbana*	82	1995

S6. Bibliometric overview of the research on *Isochrysis* sp. in 399 European scientific papers. Main concepts (a), concepts network (b), annual production (c), global collaborations (d), European collaborations (e), annual production by countries (f), main countries (g), main cities (h), emerging concepts (i), main journals (j) and main citations (k).

***Tetraselmis* sp.**

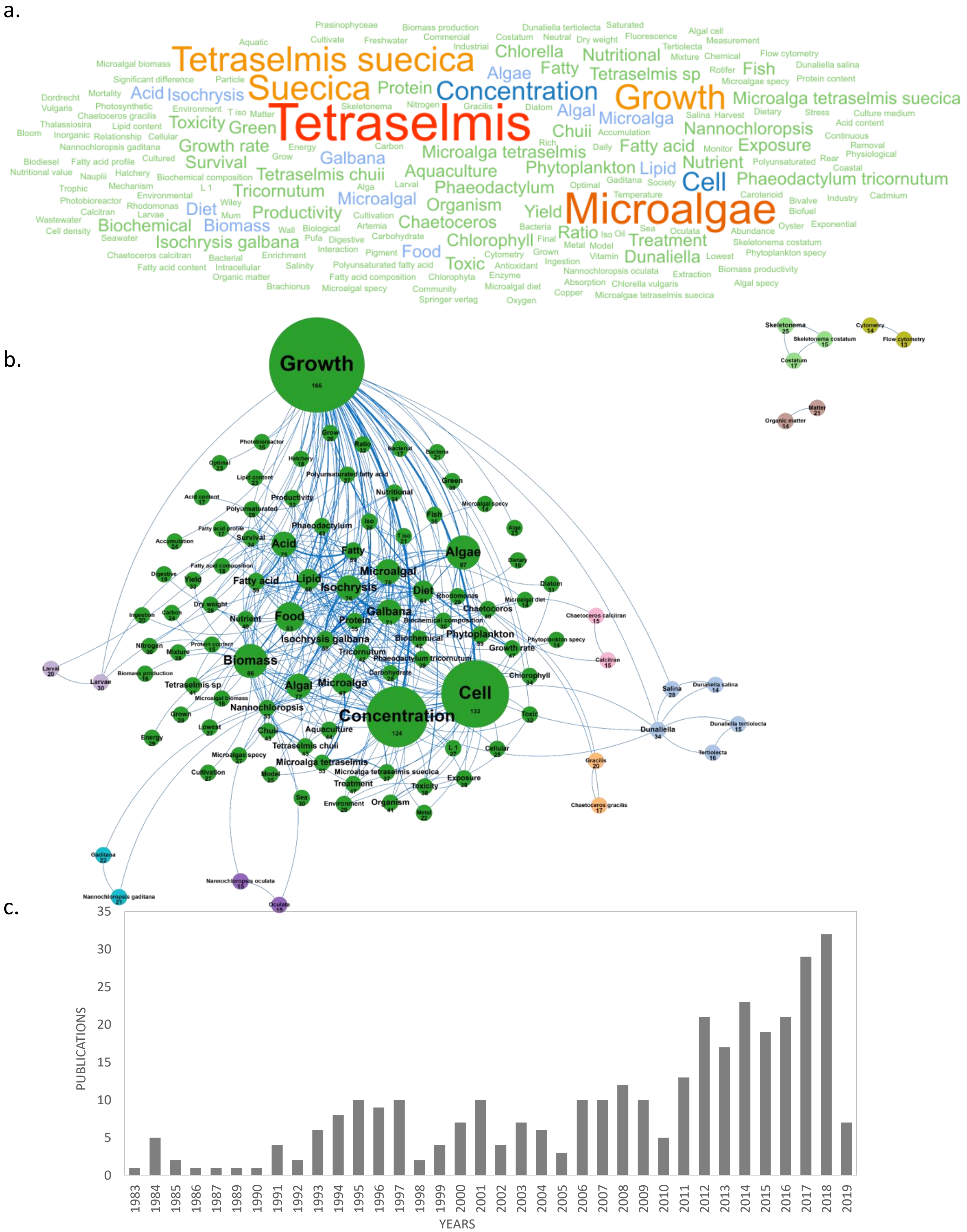

d.

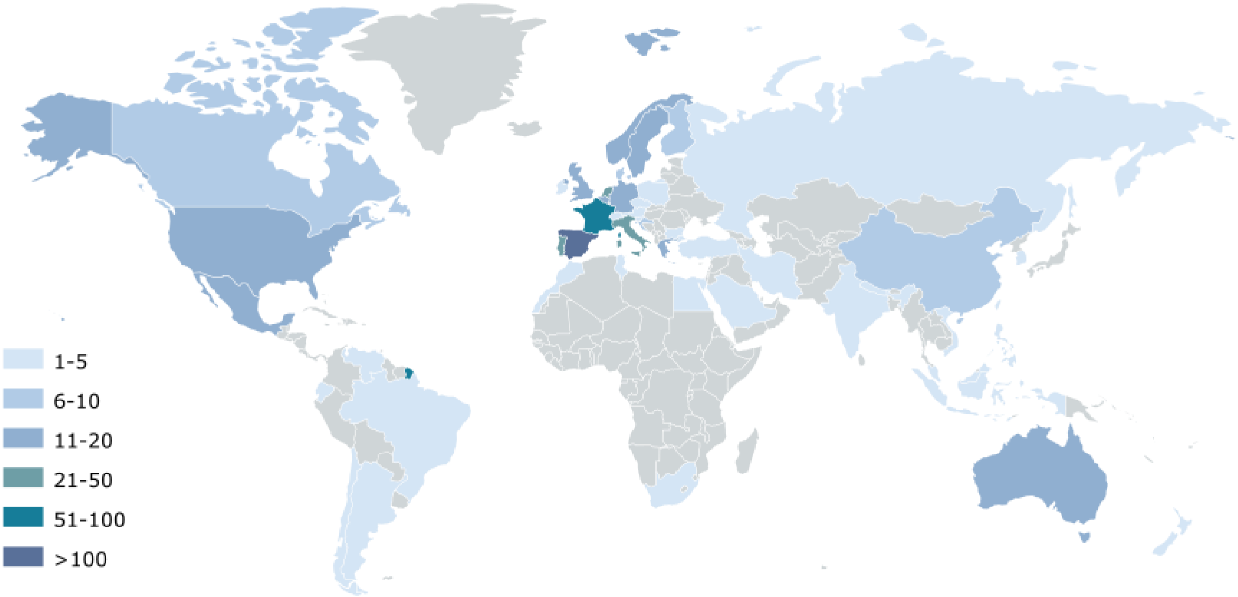

e.

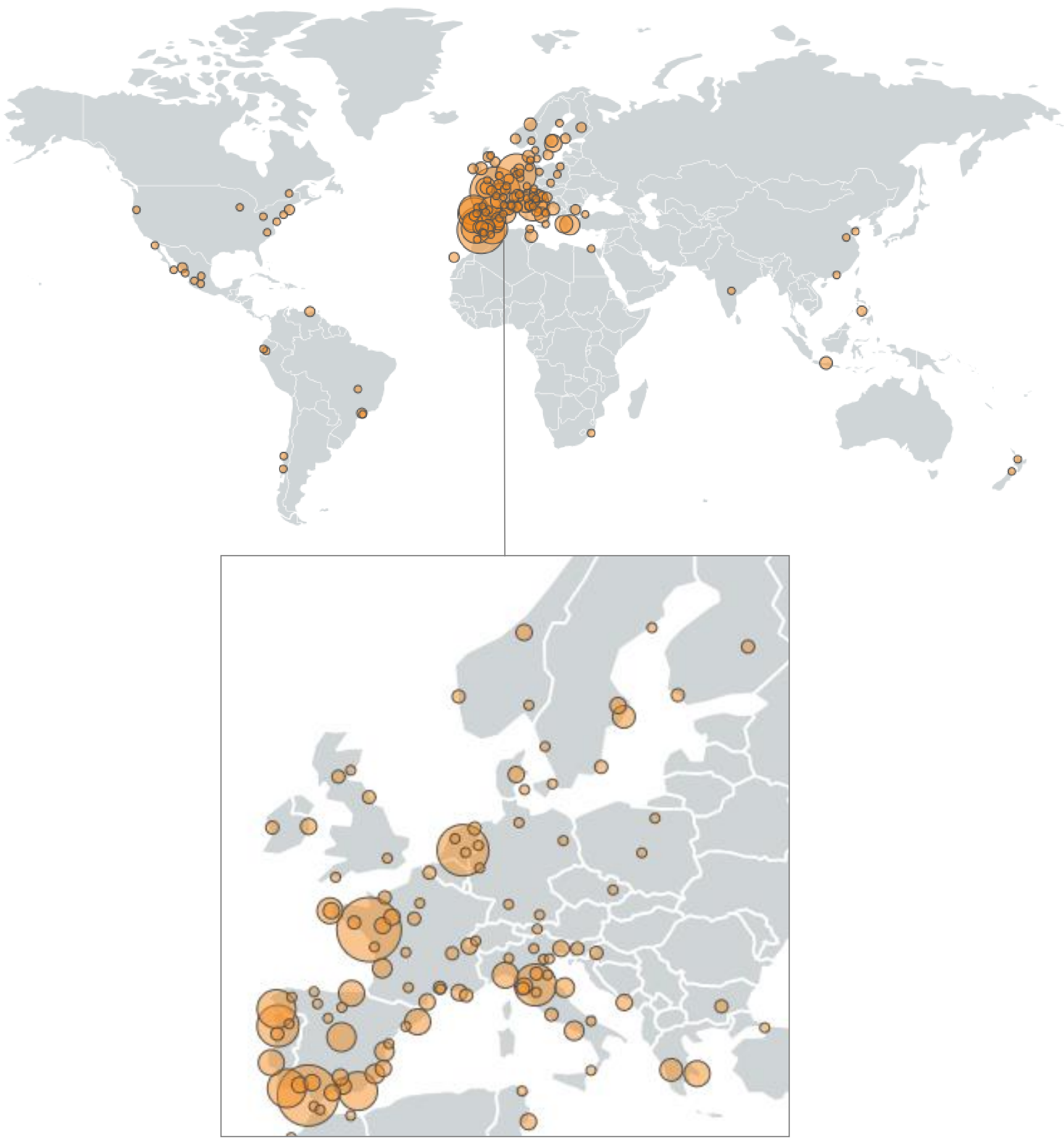

f.

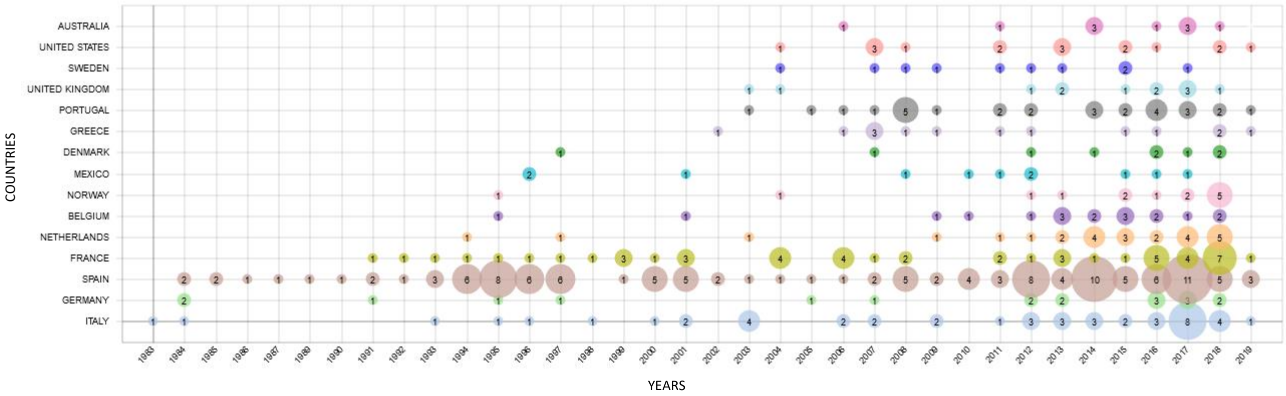

g.

Countries	Publications
SPAIN	125
FRANCE	51
ITALY	47
PORTUGAL	29
NETHERLANDS	26
GERMANY	19
BELGIUM	18
UNITED STATES	16
GREECE	14
NORWAY	14
UNITED KINGDOM	12
MEXICO	11
AUSTRALIA	10
SWEDEN	10
DENMARK	9

h.

Cities	Publications
Nantes	23
Cadiz	22
Wageningen	18
Florence	14
Porto	14
Almeria	13
Vigo	13
Faro	12
Madrid	9
Athens	8
Barcelona	8
Lisbon	8
Bilbao	7
Genova	7
Plouzane	7

i.

Emerging concepts	GF
Single specy	4
Bass	3
Dicentrarchus labrax	3
European sea bass	3
Labrax	3
Sea bass	3
Share	3
Alkaline phosphatase	2
Anthropogenic	2
Associate bacteria	2
Bacterial community	2
Biotechnological application	2
Blend	2
Call	2
Chlorella sorokiniana	2
Co 2	2
Co2 enrichment	2
Co2 injection	2
Complete diet	2
Consecutive	2

j.

Journals and number of publications	IF	Publications
AQUACULTURE	3,022	35
JOURNAL OF APPLIED PHYCOLOGY	2,635	28
BIORESOURCE TECHNOLOGY	6,669	18
AQUACULTURE RESEARCH	1,502	11
JOURNAL OF EXPERIMENTAL MARINE BIOLOGY AND ECOLOGY	2,365	10
Algal Research-Biomass Biofuels and Bioproducts	3,723	9
JOURNAL OF PLANKTON RESEARCH	2,209	6
AQUATIC TOXICOLOGY	3,794	6
AQUACULTURAL ENGINEERING	2,143	5
CHEMOSPHERE	5,108	5
ECOTOXICOLOGY AND ENVIRONMENTAL SAFETY	4,527	4
APPLIED MICROBIOLOGY AND BIOTECHNOLOGY	3,67	4
CHEMISTRY AND ECOLOGY	1,214	4
AQUACULTURE INTERNATIONAL	1,455	4
AQUACULTURE NUTRITION	2,098	4

k.

Title of publications	Citations	Date
Production of microalgal concentrates by flocculation and their assessment as aquaculture feeds	195	2006
Biomass production and variation in the biochemical profile (total protein, carbohydrates, RNA, lipids and fatty acids) of seven species of marine microalgae	190	1989
Productivity and photosynthetic efficiency of outdoor cultures of *Tetraselmis suecica* in annular columns	137	2006
Oil production by the marine microalgae *Nannochloropsis* sp. F&M-M24 and *Tetraselmis suecica* F&M-M33	130	2012
Antioxidant potential of microalgae in relation to their phenolic and carotenoid content	119	2012
Potential for green microalgae to produce hydrogen, pharmaceuticals and other high value products in a combined process	113	2013
Growth of the marine microalga *Tetraselmis suecica* in batch cultures with different salinities and nutrient concentrations	101	1984
Cadmium removal by living cells of the marine microalga *Tetraselmis suecica*	96	2002
Isolation and characterization of soluble protein from the green microalgae *Tetraselmis* sp	88	2011
Toxicity and bioaccumulation of copper and lead in five marine microalgae	88	2009
Iron-mediated effects on nitrate reductase in marine phytoplankton	85	1994
Estimation of oxygen evolution by marine phytoplankton from measurement of the efficiency of Photosystem II electron flow	83	1997
Ratio between autoflocculating and target microalgae affects the energy-efficient harvesting by bio-flocculation	77	2012
Vitamin E (alpha-tocopherol) production by the marine microalgae *Dunaliella tertiolecta* and *Tetraselmis suecica* in batch cultivation	75	2003
Growth inhibition of cultured marine phytoplankton by toxic algal-derived polyunsaturated aldehydes	74	2007

S7. Bibliometric overview of the research on *Tetraselmis* sp. in 333 European scientific papers. Main concepts (a), concepts network (b), annual production (c), global collaborations (d), European collaborations (e), annual production by countries (f), main countries (g), main cities (h), emerging concepts (i), main journals (j) and main citations (k).

***Arthrospira* sp.**

a.

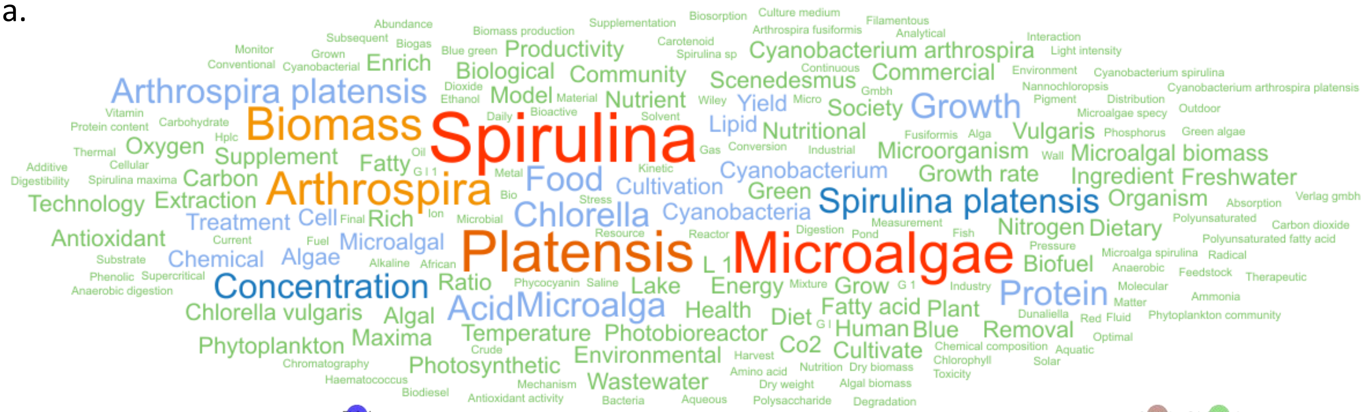

b.

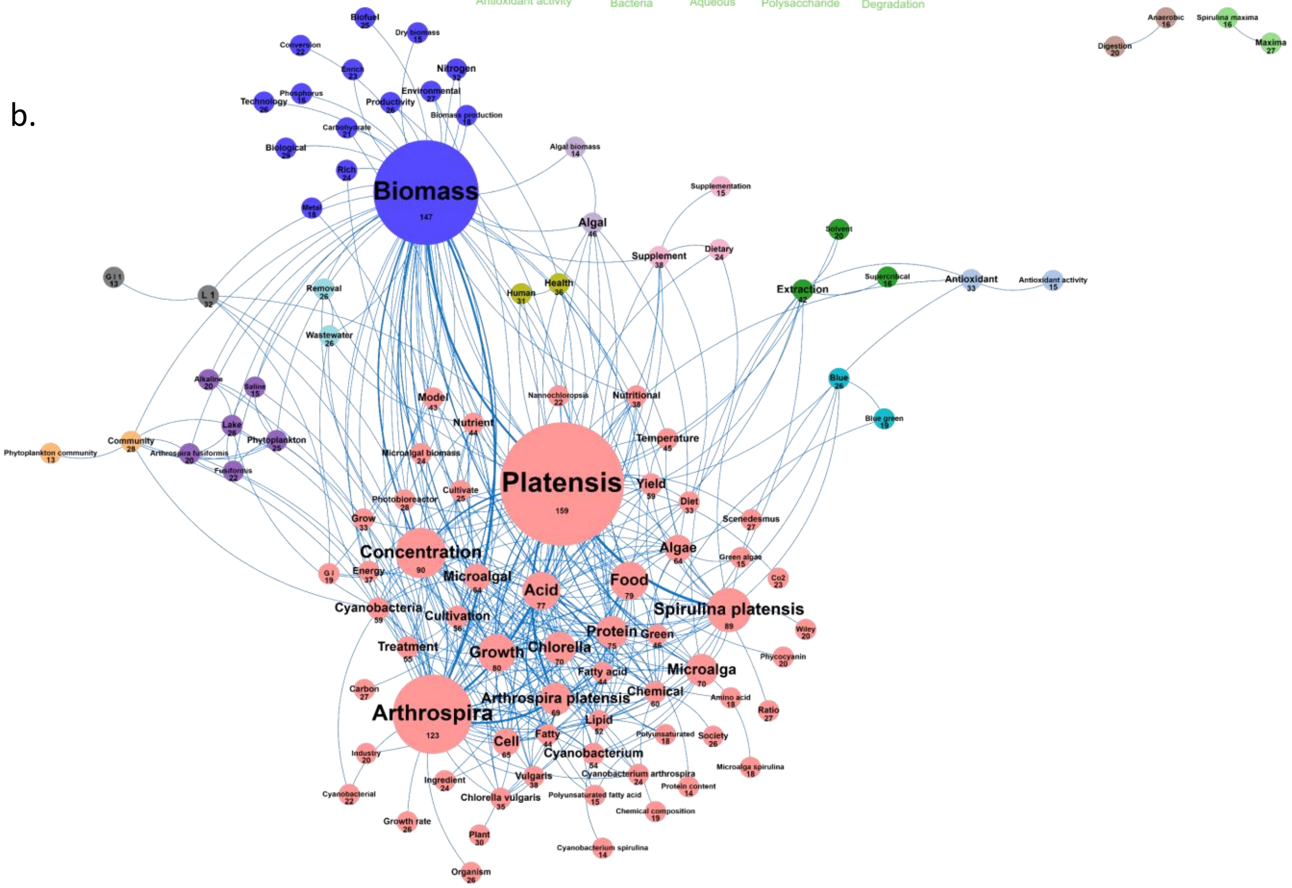

c.

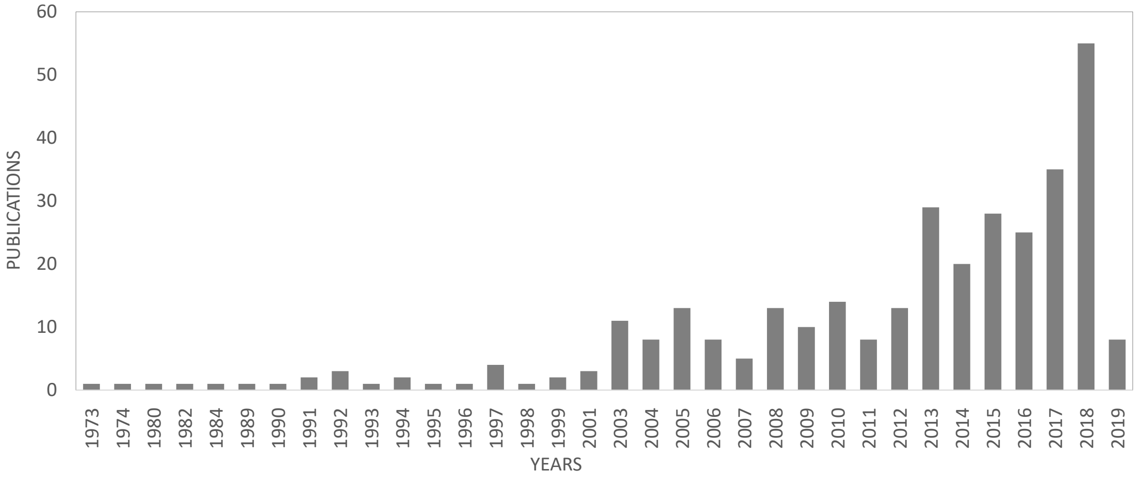

d.

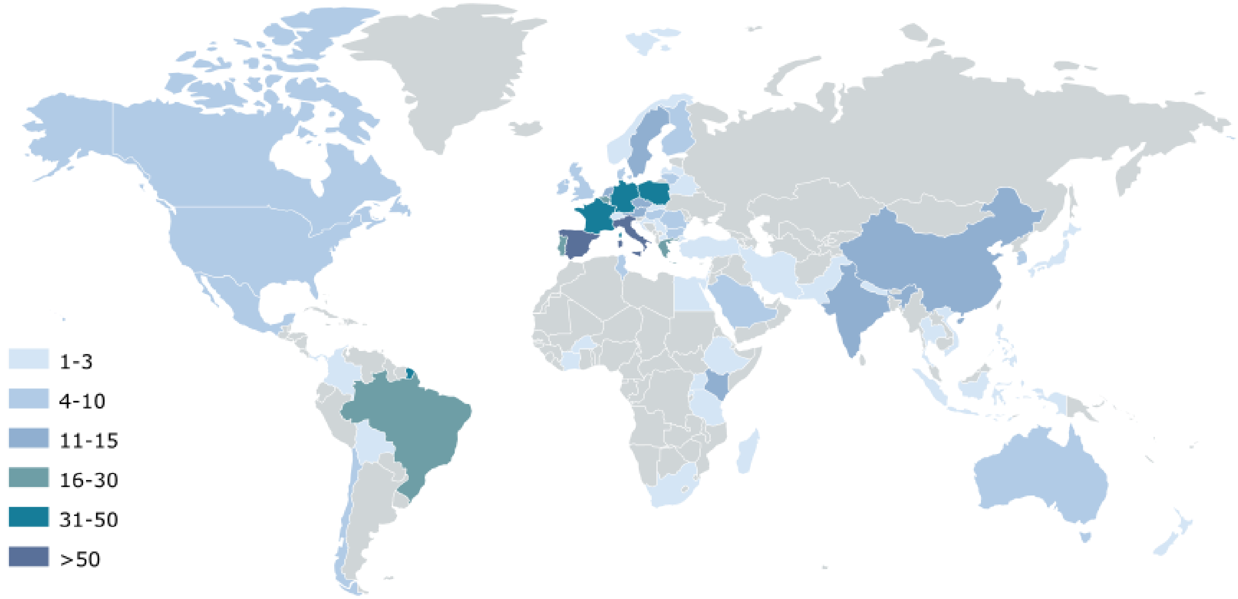

e.

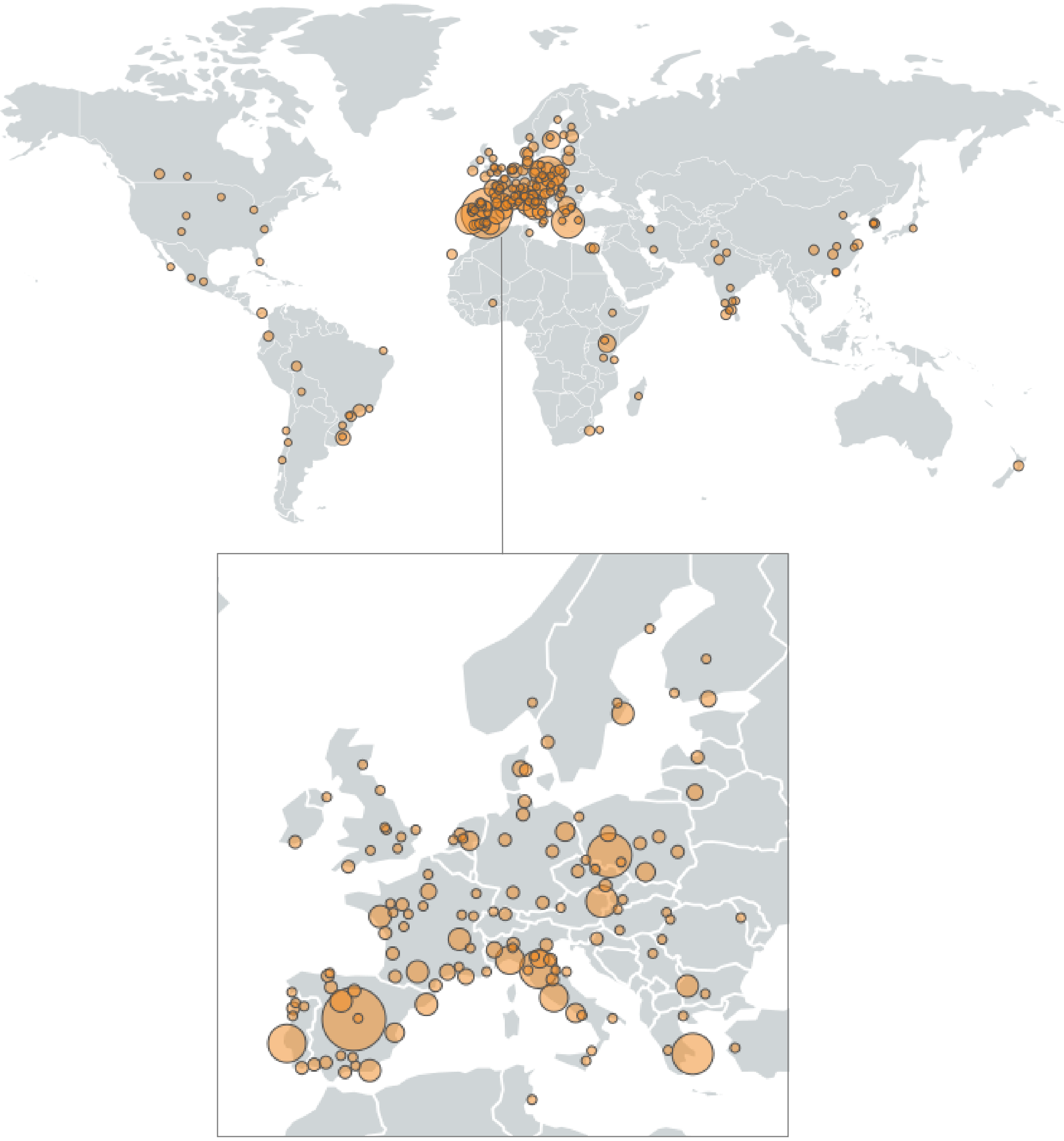

f.

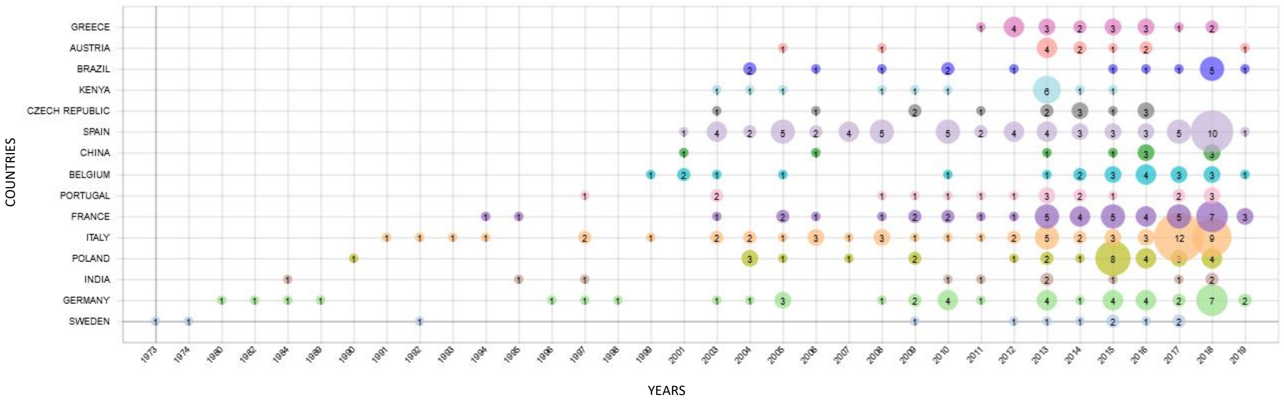

g.

Countries	Publications
SPAIN	63
ITALY	58
FRANCE	46
GERMANY	44
POLAND	31
BELGIUM	23
GREECE	19
PORTUGAL	19
BRAZIL	16
CZECH REPUBLIC	14
KENYA	14
AUSTRIA	12
SWEDEN	12
INDIA	11
CHINA	10

h.

Cities	Publications
Madrid	25
Wroclaw	17
Athens	15
Florence	14
Lisbon	14
Vienna	11
Genova	10
Rome	10
Barcelona	7
Lyon	7
Nairobi	7
Nantes	7
Stockholm	7
Almeria	6
Sofia	6

i.

Emerging concepts	GF
Springer nature	5
Alternative protein	4
Alternative protein source	4
Bean	4
Proximate	4
Soy	4
Stream	4
Substitution	4
Biomass cultivation	3
Continuous system	3
Corn	3
Differential	3
Energy return	3
Fourier	3
Fourier transform	3
Gastrointestinal	3
Glycerol	3
Grain	3
Grass	3
Htl	3

j.

Journals and number of publications	IF	Publications
BIORESOURCE TECHNOLOGY	6,669	28
JOURNAL OF APPLIED PHYCOLOGY	2,635	19
Algal Research-Biomass Biofuels and Bioproducts (	3,723	18
HYDROBIOLOGIA	2,325	7
FOOD CHEMISTRY	5,399	7
JOURNAL OF INDUSTRIAL MICROBIOLOGY & BIOTECHNOLOGY	2,993	5
JOURNAL OF SUPERCRITICAL FLUIDS	3,481	4
JOURNAL OF FOOD ENGINEERING	3,625	4
Energies	2,707	4
PLoS One	2,776	3
JOURNAL OF ANALYTICAL AND APPLIED PYROLYSIS	3,47	3
JOURNAL OF AGRICULTURAL AND FOOD CHEMISTRY	3,571	3
FUEL	5,128	3
ELECTROPHORESIS	2,754	3
ENVIRONMENTAL TECHNOLOGY	1,918	3

k.

Title of publications	Citations	Date
Microalgae as a raw material for biofuels production	727	2009
Biosorption of Cr3 , Cd2 and Cu2 ions by blue-green algae *Spirulina* sp.: Kinetics, equilibrium and the mechanism of the process	366	2005
FATTY ACID CONTENT AND CHEMICAL COMPOSITION OF FRESHWATER MICROALGAE	338	1992
Microalgae as substrates for fermentative biogas production in a combined biorefinery concept	332	2010
Supercritical carbon dioxide extraction of compounds with pharmaceutical importance from microalgae	271	2003
Potential carbon dioxide fixation by industrially important microalgae	239	2010
Protein measurements of microalgal and cyanobacterial biomass	233	2010
Antioxidant activity of different fractions of *Spirulina platensis* protean extract	229	2001
Cultivation of filamentous cyanobacteria (blue-green algae) in agro-industrial wastes and wastewaters: A review	220	2011
Evaluation of *Spirulina* sp. growth in photoautotrophic, heterotrophic and mixotrophic cultures	174	2004
Nondestructive tracing of migratory rhythms of intertidal benthic microalgae using in vivo chlorophyll a fluorescence	160	1997
The role of rabbit meat as functional food	149	2011
From open ponds to vertical alveolar panels: the Italian experience in the development of reactors for the mass cultivation of phototrophic microorganisms	148	1992
Optimization of accelerated solvent extraction of antioxidants from *Spirulina platensis* microalga	144	2005
A vertical alveolar panel (VAP) for outdoor mass cultivation of microalgae and cyanobacteria	135	1991

S8. Bibliometric overview of the research on *Arthrospira* sp. in 330 European scientific papers. Main concepts (a), concepts network (b), annual production (c), global collaborations (d), European collaborations (e), annual production by countries (f), main countries (g), main cities (h), emerging concepts (i), main journals (j) and main citations (k).

***Selenastrum* sp.**

a.

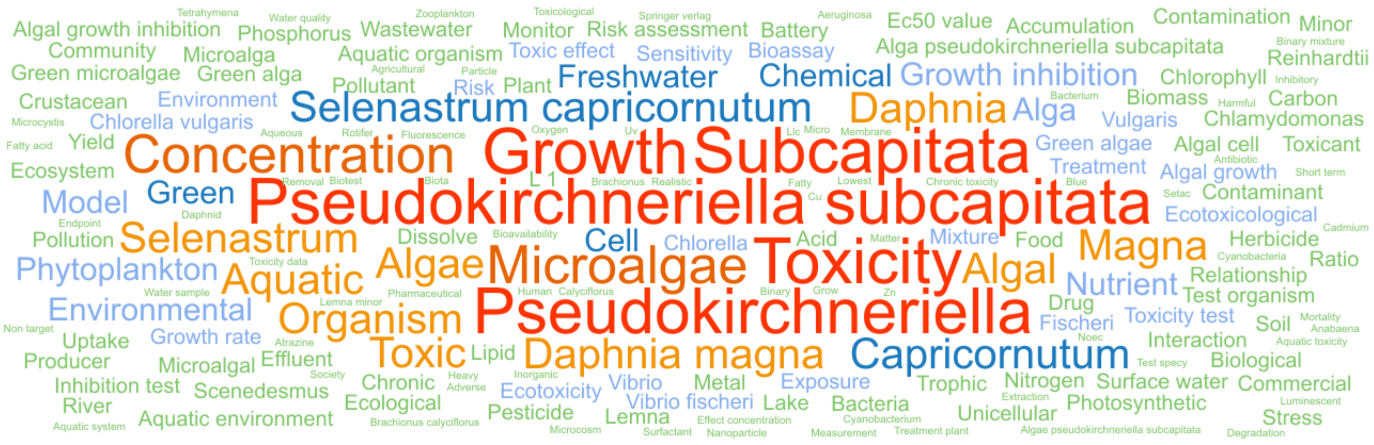

b.

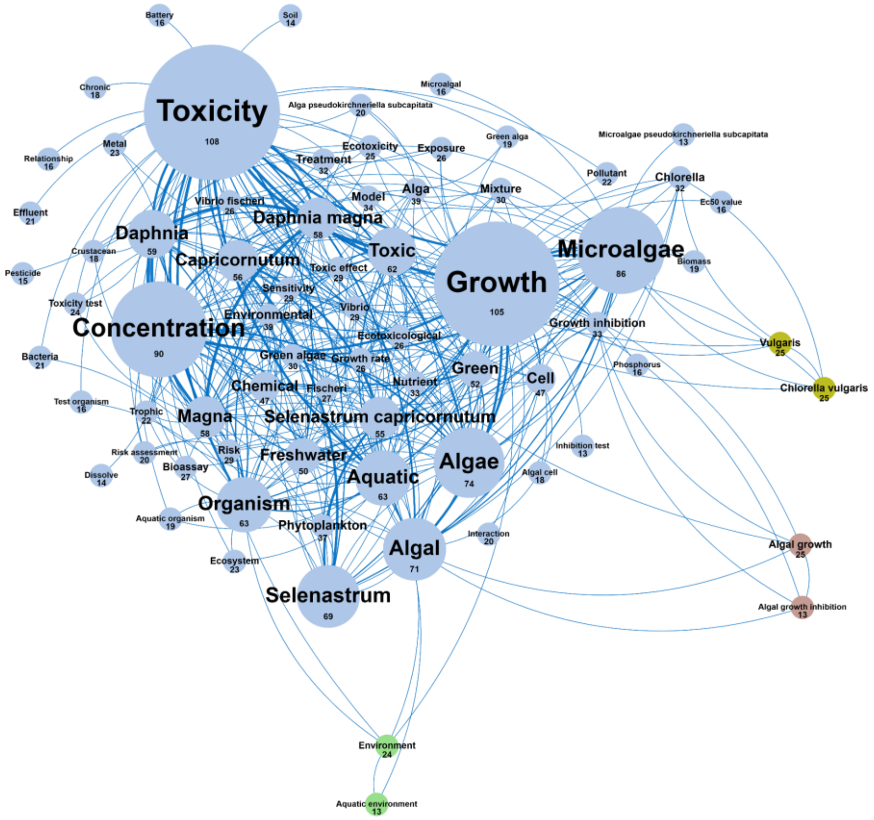

c.

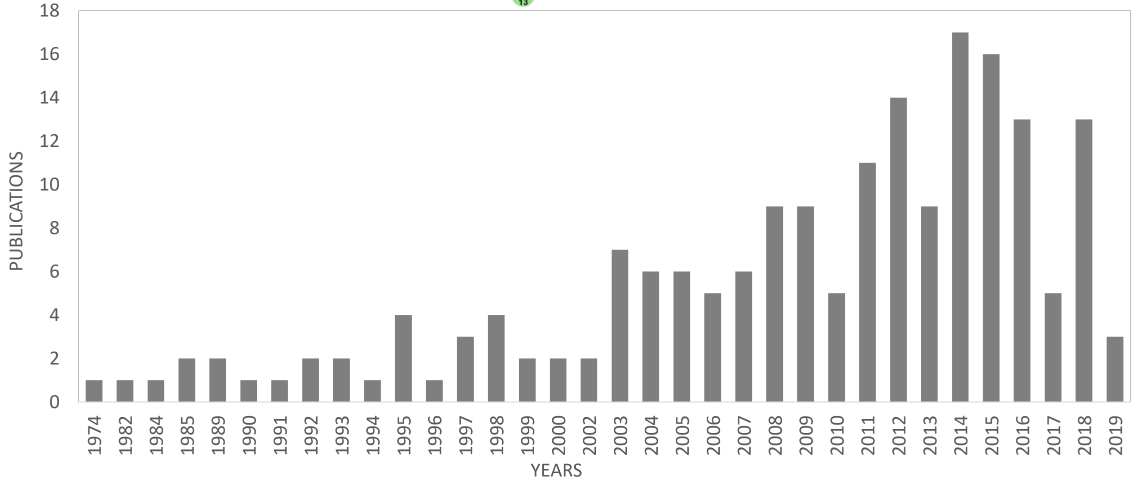

d.

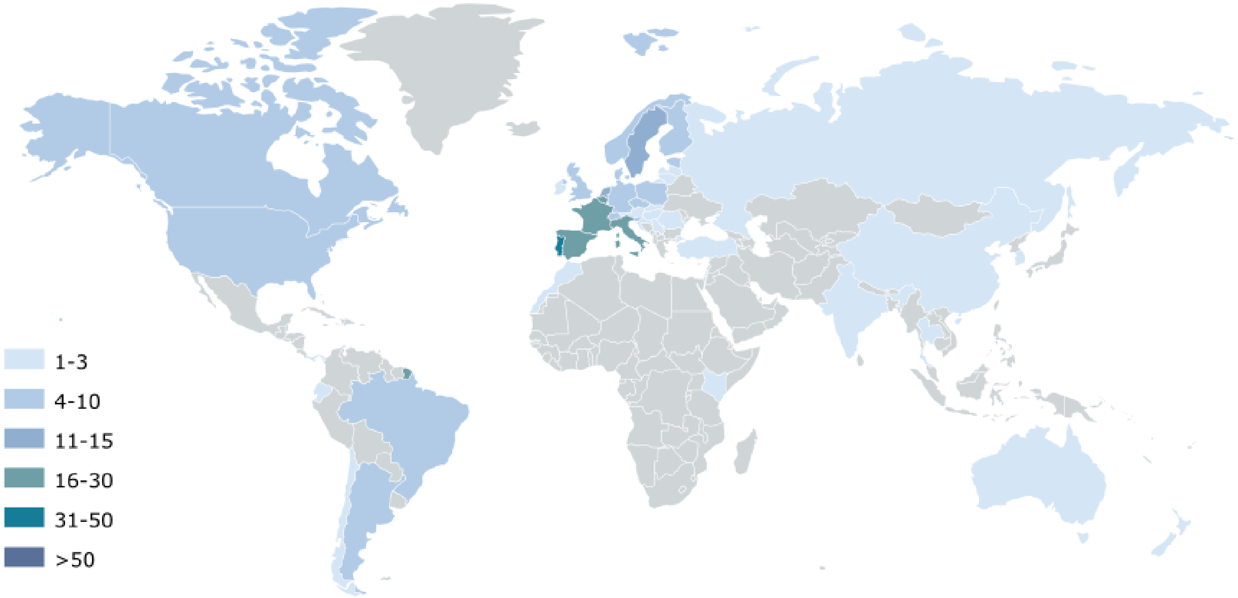

e.

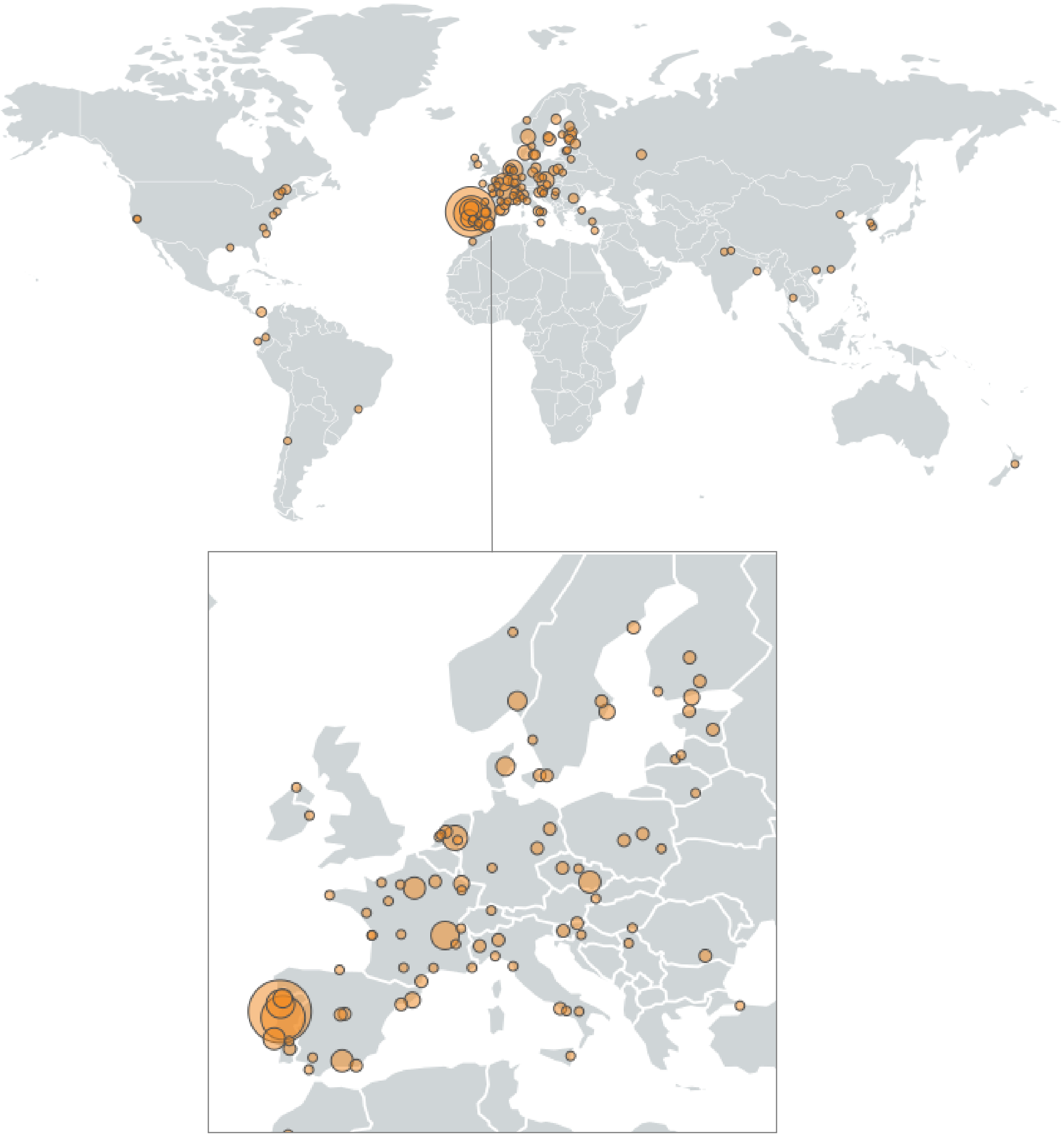

f.

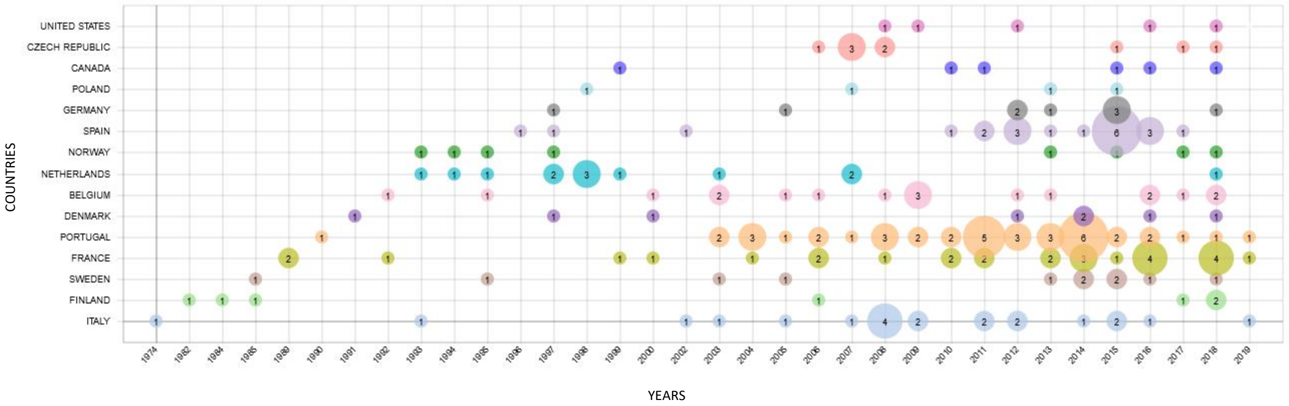

g.

Countries	Publications
PORTUGAL	41
FRANCE	28
ITALY	21
SPAIN	21
BELGIUM	18
NETHERLANDS	13
SWEDEN	11
CZECH REPUBLIC	9
GERMANY	9
DENMARK	8
NORWAY	8
FINLAND	7
CANADA	6
UNITED STATES	5
POLAND	4

h.

Cities	Publications
Aveiro	25
Coimbra	16
Lyon	9
Porto	9
Wageningen	8
Brno	7
Granada	7
Lisbon	6
Paris	6
Braga	5
Lyngby	5
Oslo	5
Barcelona	4
Helsinki	4
Metz	4

i.

Emerging concepts	GF
Biochemical	3
Freshwater microalga	3
Additivity	2
Algal culture	2
Amend	2
Biochemical composition	2
Biomass production	2
Calibrate	2
Dha	2
Ecosar	2
Euglena	2
Euglena gracilis	2
Fate	2
Freshwater microalga pseudokirchneriella subcapitata	2
Friendly	2
Gracilis	2
Macrolide	2
Macrolide antibiotic	2
Mix algal	2
Mix algal culture	2

j.

Journals and number of publications	IF	Publications
CHEMOSPHERE	5,108	16
ECOTOXICOLOGY AND ENVIRONMENTAL SAFETY	4,527	12
ENVIRONMENTAL TOXICOLOGY AND CHEMISTRY	3,421	12
ECOTOXICOLOGY	2,46	9
FRESENIUS ENVIRONMENTAL BULLETIN	0,691	7
SCIENCE OF THE TOTAL ENVIRONMENT	5,589	7
AQUATIC TOXICOLOGY	3,794	6
HYDROBIOLOGIA	2,325	5
ENVIRONMENTAL POLLUTION	5,714	5
ENVIRONMENTAL SCIENCE AND POLLUTION RESEARCH	2,914	5
JOURNAL OF HAZARDOUS MATERIALS	7,65	5
ENVIRONMENTAL TOXICOLOGY	2,649	5
WATER RESEARCH	7,913	5
WATER AIR AND SOIL POLLUTION	1,774	4
JOURNAL OF APPLIED PHYCOLOGY	2,635	4

k.

Title of publications	Citations	Date
Toxicity of nanoparticles of CuO, ZnO and TiO2 to microalgae *Pseudokirchneriella subcapitata*	699	2009
Algal toxicity of antibacterial agents used in intensive farming	299	2000
Toxicity of five antibiotics and their mixtures towards photosynthetic aquatic organisms: Implications for environmental risk assessment	236	2013
Altered cell wall morphology in nutrient-deficient phytoplankton and its impact on grazers	183	1997
Toxicity evaluation of single and mixed antifouling biocides measured with acute toxicity bioassays	170	2002
Comparative use of bacterial, algal and protozoan tests to study toxicity of azo- and anthraquinone dyes	161	2006
Degradation of diclofenac by TiO2 photocatalysis: UV absorbance kinetics and process evaluation through a set of toxicity bioassays	157	2009
Combined exposure to hydrogen peroxide and light-selective effects on cyanobacteria, green algae, and diatoms	135	2007
Grazing resistance in nutrient-stressed phytoplankton	125	1993
Designing ionic liquids: The chemical structure role in the toxicity	113	2013
Potential for green microalgae to produce hydrogen, pharmaceuticals and other high value products in a combined process	113	2013
Biochar properties regarding to contaminants content and ecotoxicological assessment	110	2013
Toxicity evaluation of three pesticides on non-target aquatic and soil organisms: Commercial formulation versus active ingredient	107	2009
Physicochemical characterization and ecotoxicological assessment of CeO2 nanoparticles using two aquatic microorganisms	100	2011
Differential sensitivity of green algae to allelopathic substances from Chara	100	2003

S9. Bibliometric overview of the research on *Selenastrum* sp. in 186 European scientific papers. Main concepts (a), concepts network (b), annual production (c), global collaborations (d), European collaborations (e), annual production by countries (f), main countries (g), main cities (h), emerging concepts (i), main journals (j) and main citations (k).

***Botryococcus* sp.**

a.

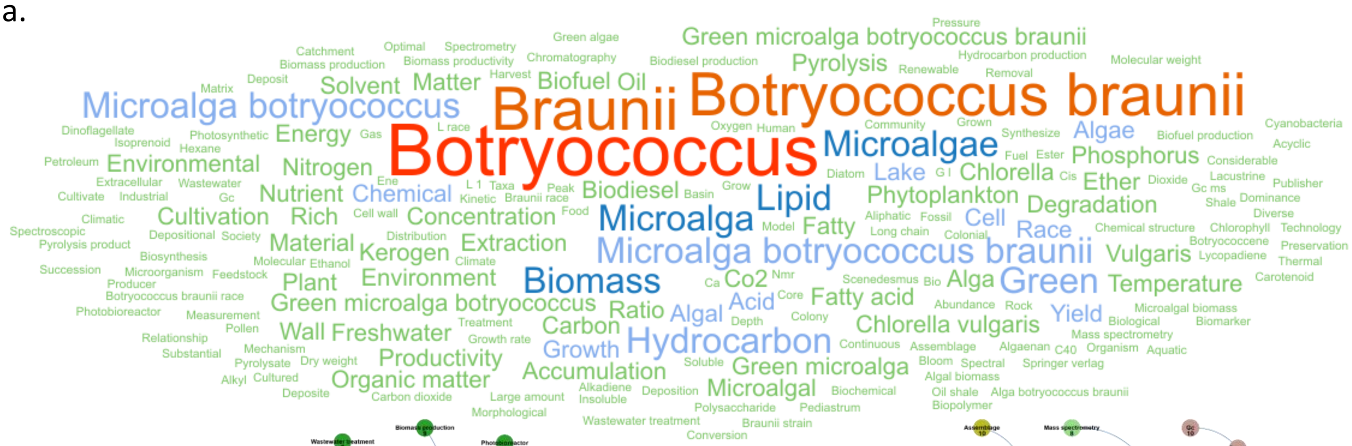

b.

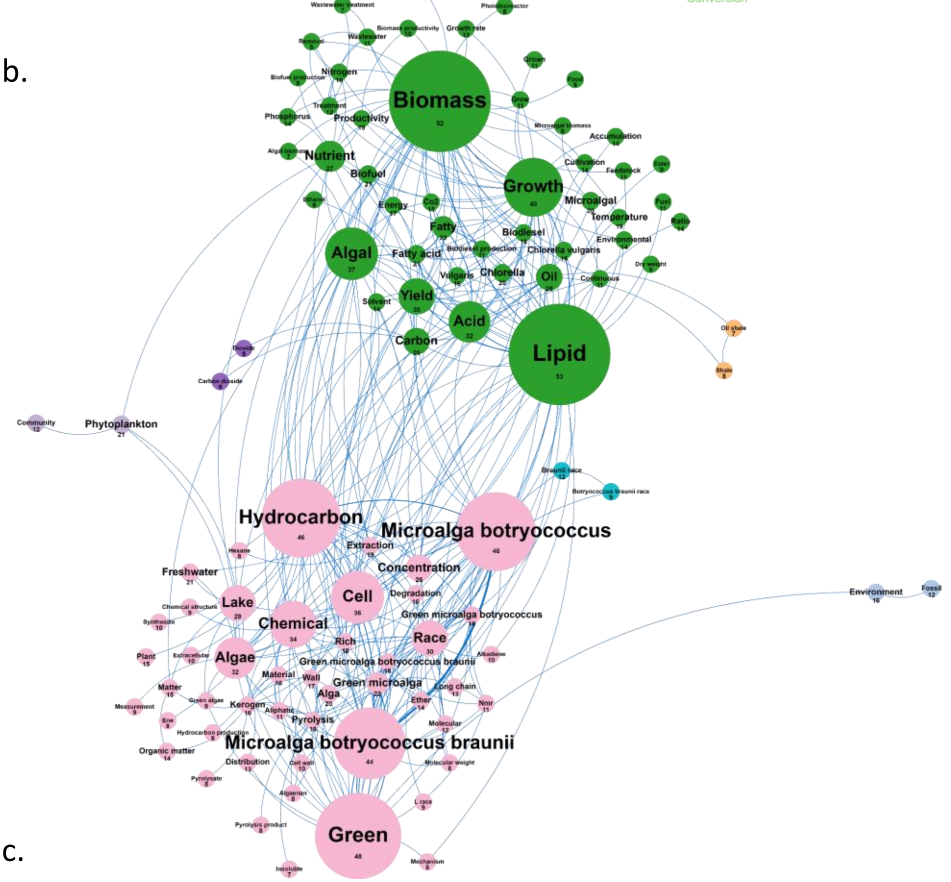

c.

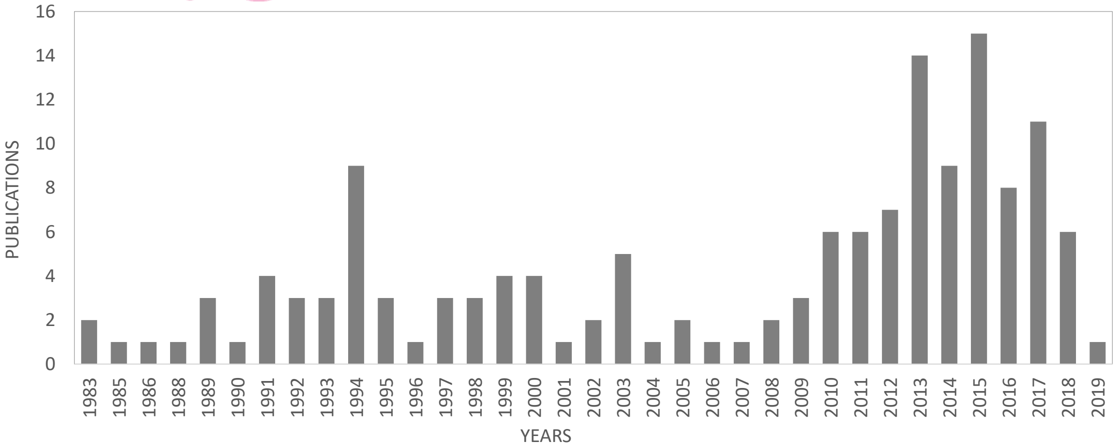

d.

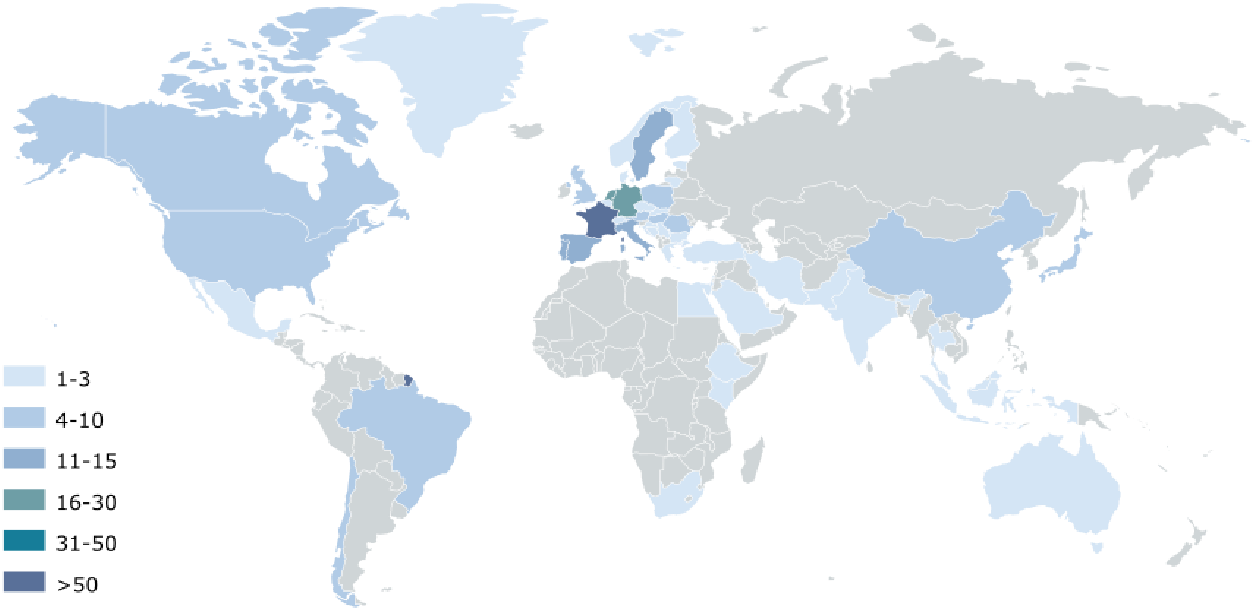

e.

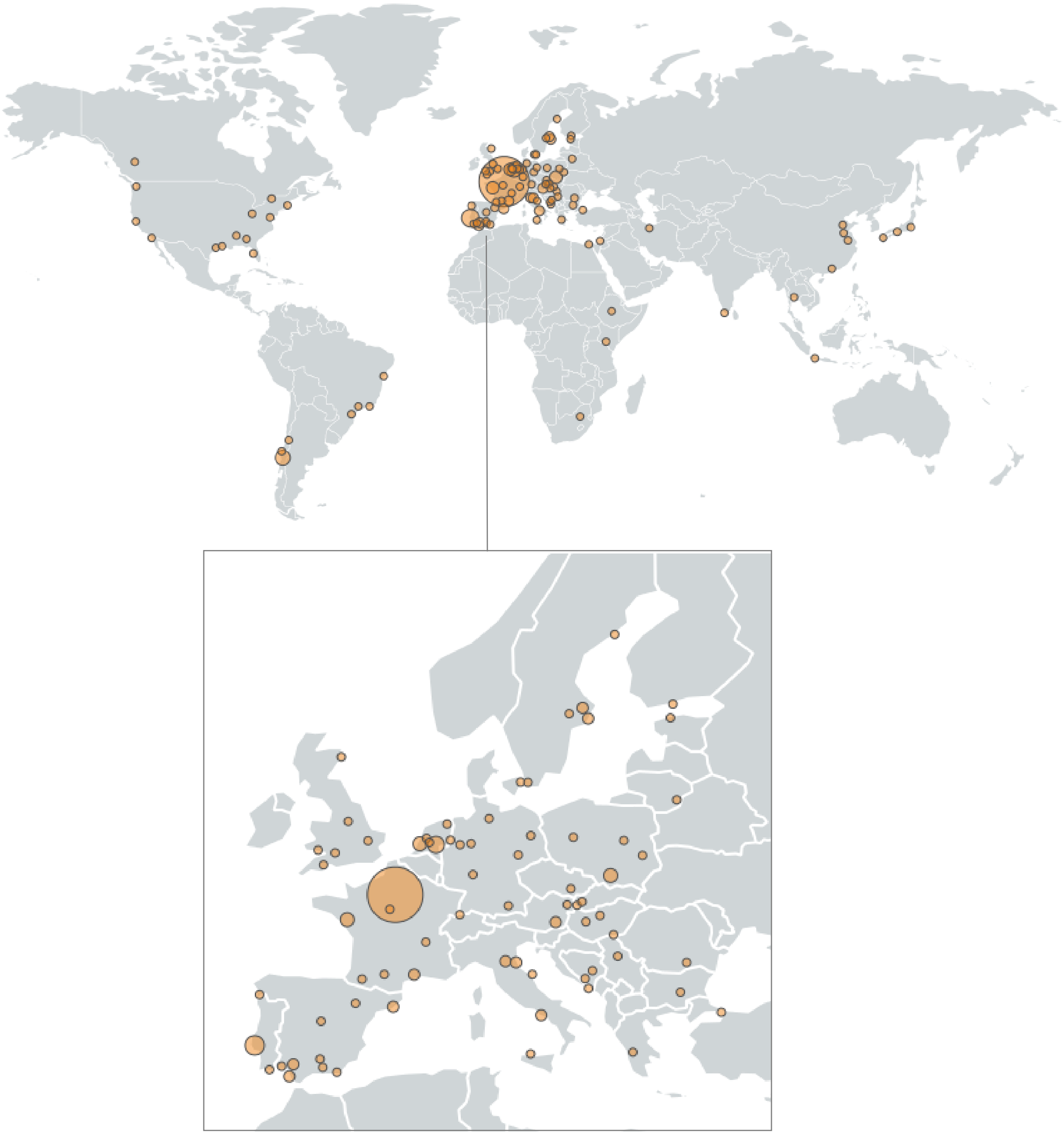

f.

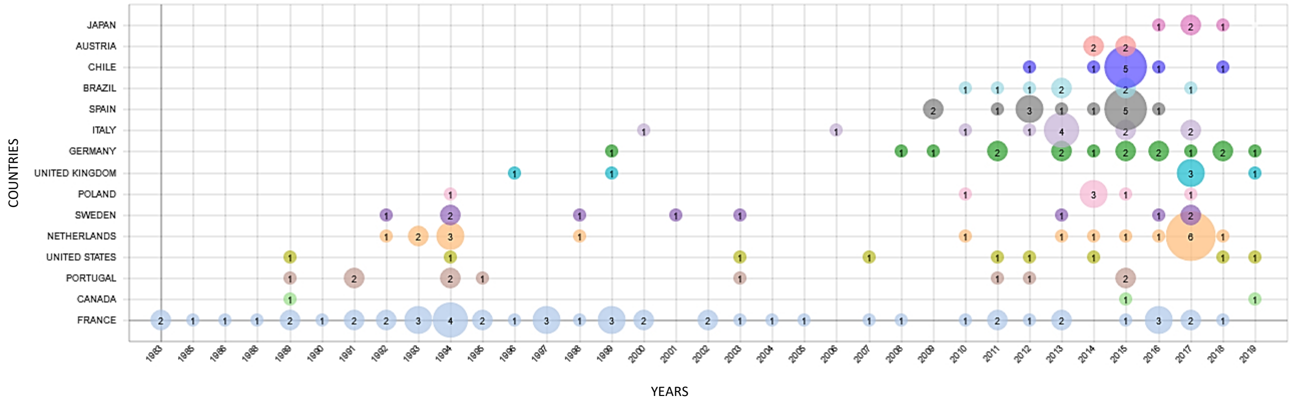

g.

Countries	Publications
FRANCE	51
NETHERLANDS	19
GERMANY	16
SPAIN	14
ITALY	12
PORTUGAL	11
SWEDEN	10
CHILE	9
UNITED STATES	9
BRAZIL	8
POLAND	7
UNITED KINGDOM	6
AUSTRIA	4
JAPAN	4
CANADA	3

h.

Cities	Publications
Paris	38
Lisbon	10
Temuco	8
Wageningen	8
Delft	5
Krakow	5
Nantes	5
Bologna	4
Ravenna	4
Stockholm	4
Barcelona	3
Cadiz	3
Graz	3
Montpellier	3
Naples	3

i.

Emerging concepts	GF
Absorption	2
Fatty acid composition	2
Accumulation response	1
Adaptive	1
Adaptive cell response	1
Adna	1
Adna sequence	1
Algae cultivation	1
Algae specy	1
Algal system	1
Alkaline medium	1
Ally	1
Alpha linolenic	1
Alpha linolenic acid	1
Analize	1
Ancient	1
Ancient dna	1
Aquaculture	1
Aquaculture effluent	1
Aquaculture production	1

j.

Journals and number of publications	IF	Publications
ORGANIC GEOCHEMISTRY	3,12	15
PHYTOCHEMISTRY	2,905	10
JOURNAL OF APPLIED PHYCOLOGY	2,635	8
BIORESOURCE TECHNOLOGY	6,669	7
Algal Research-Biomass Biofuels and Bioproducts	3,723	6
APPLIED MICROBIOLOGY AND BIOTECHNOLOGY	3,67	4
GEOCHIMICA ET COSMOCHIMICA ACTA	4,258	4
HYDROBIOLOGIA	2,325	4
PALAEOGEOGRAPHY PALAEOCLIMATOLOGY PALAEOECOLOGY	2,616	3
ENZYME AND MICROBIAL TECHNOLOGY	3,553	2
APPLIED ENERGY	8,426	2
FUEL	5,128	2
JOURNAL OF ANALYTICAL AND APPLIED PYROLYSIS	3,47	2
POLAR BIOLOGY	2,002	2
REVIEW OF PALAEOBOTANY AND PALYNOLOGY	1,674	2

k.

Title of publications	Citations	Date
An outlook on microalgal biofuels	1049	2010
*Botryococcus b*raunii: A rich source for hydrocarbons and related ether lipids	435	2005
Supercritical carbon dioxide extraction of compounds with pharmaceutical importance from microalgae	271	2003
Potential carbon dioxide fixation by industrially important microalgae	239	2010
Glacial-interglacial environmental changes inferred from molecular and compound-specific delta13C analyses of sediments from Sacred Lake, Mt. Kenya	169	1999
Screening of microalgae with potential for biodiesel production and nutrient removal from treated domestic sewage	134	2011
Antioxidant potential of microalgae in relation to their phenolic and carotenoid content	119	2012
Potential for green microalgae to produce hydrogen, pharmaceuticals and other high value products in a combined process	113	2013
Extraction of hydrocarbons from microalga *Botryococcus braunii* with switchable solvents	107	2010
Raman microspectroscopy of individual algal cells: Sensing unsaturation of storage lipids in vivo	94	2010
Possible algal origin of long chain odd n-alkanes in immature sediments as revealed by distributions and carbon isotope ratios	93	1994
Phytoplankton dynamics and structure: A comparative analysis in natural and man-made water bodies of different trophic state	83	2000
Growth of the microalga *Botryococcus braunii* in secondarily treated sewage	75	2009
Similar morphological and chemical variations of *Gloeocapsomorpha prisca* in Ordovician sediments and cultured *Botryococcus braunii* as a response to changes in salinity	75	1992
A remarkable paradox: Sulfurised freshwater algal (*Botryococcus braunii*) lipids in an ancient hypersaline euxinic ecosystem	74	1998

S10. Bibliometric overview of the research on *Botryococcus* sp. in 147 European scientific papers. Main concepts (a), concepts network (b), annual production (c), global collaborations (d), European collaborations (e), annual production by countries (f), main countries (g), main cities (h), emerging concepts (i), main journals (j) and main citations (k).

***Haematococcus* sp.**

a.

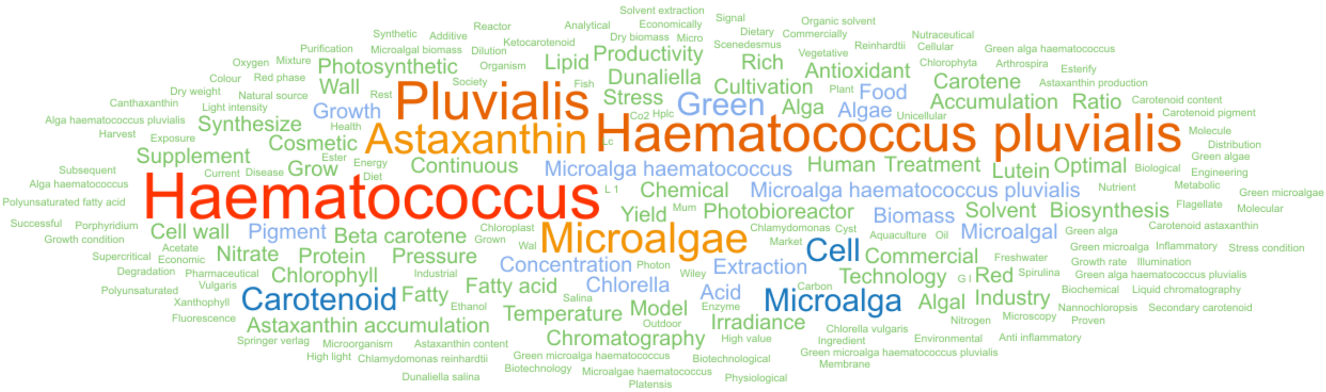

b.

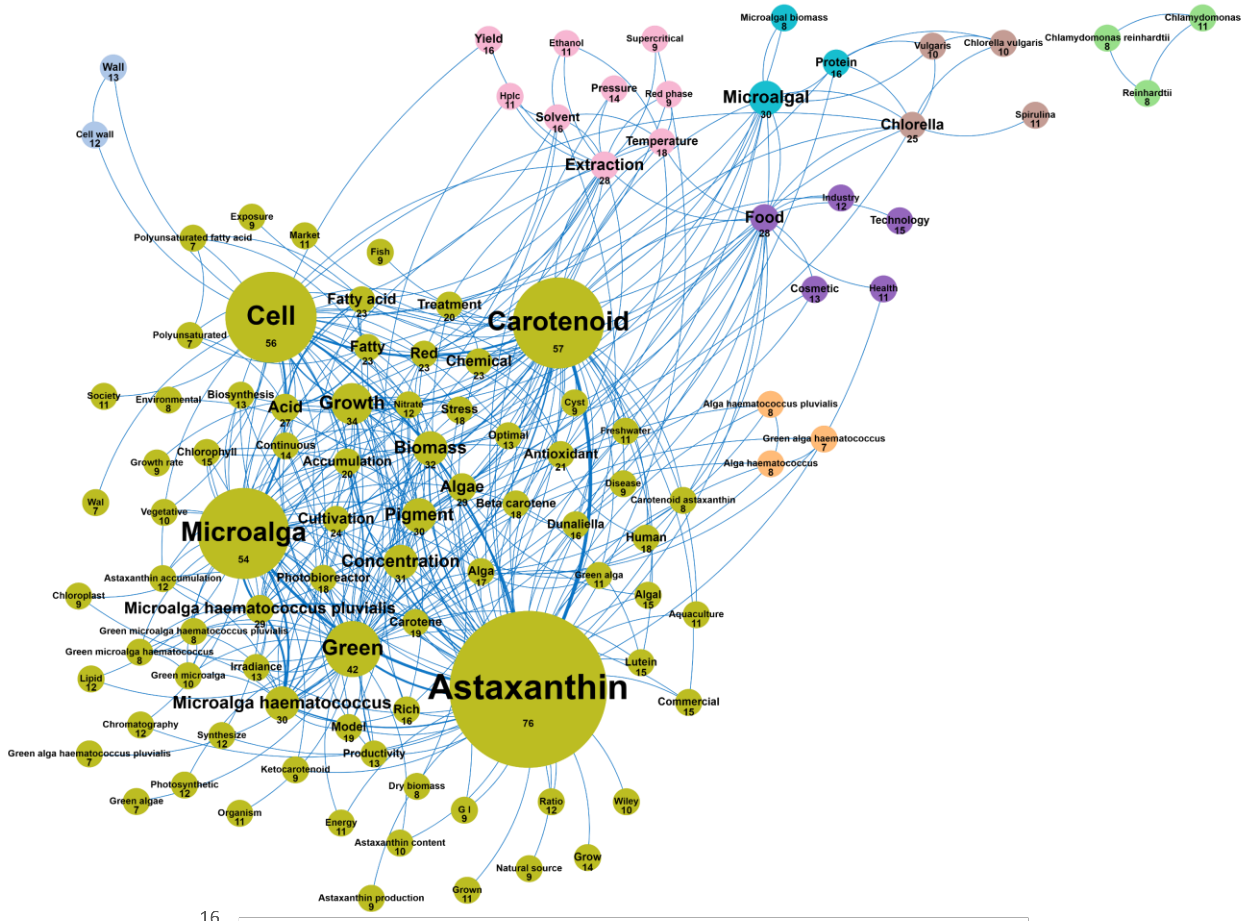

c.

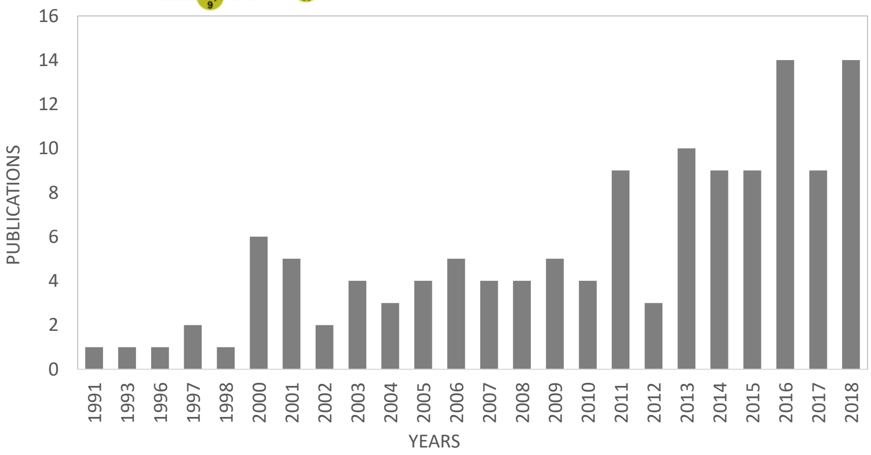

d.

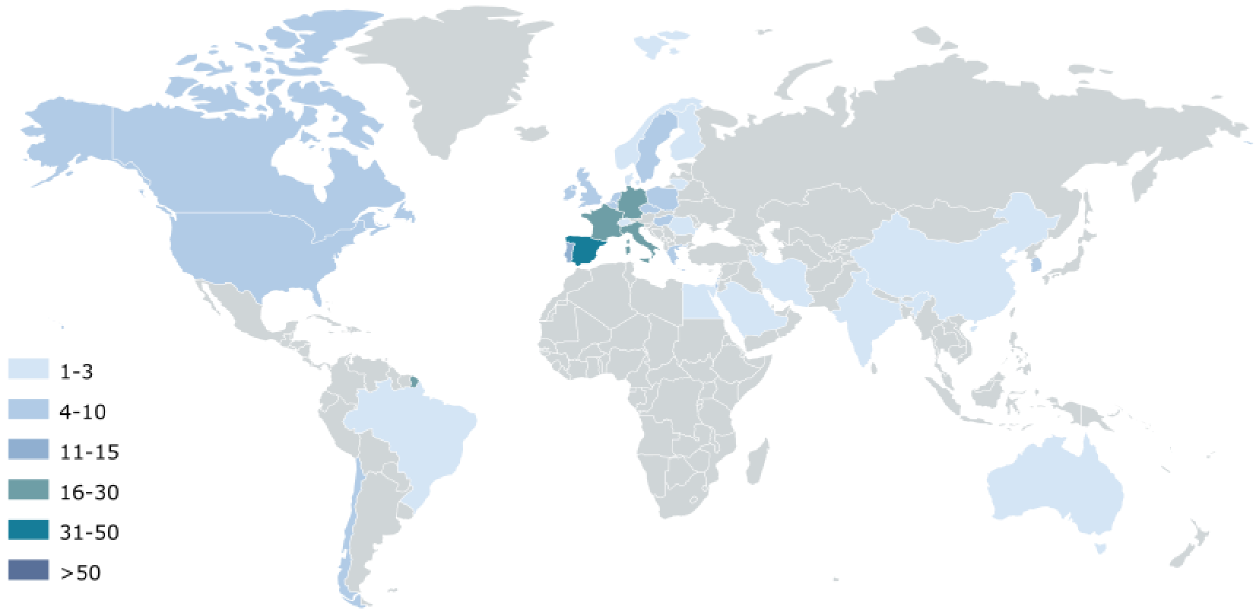

e.

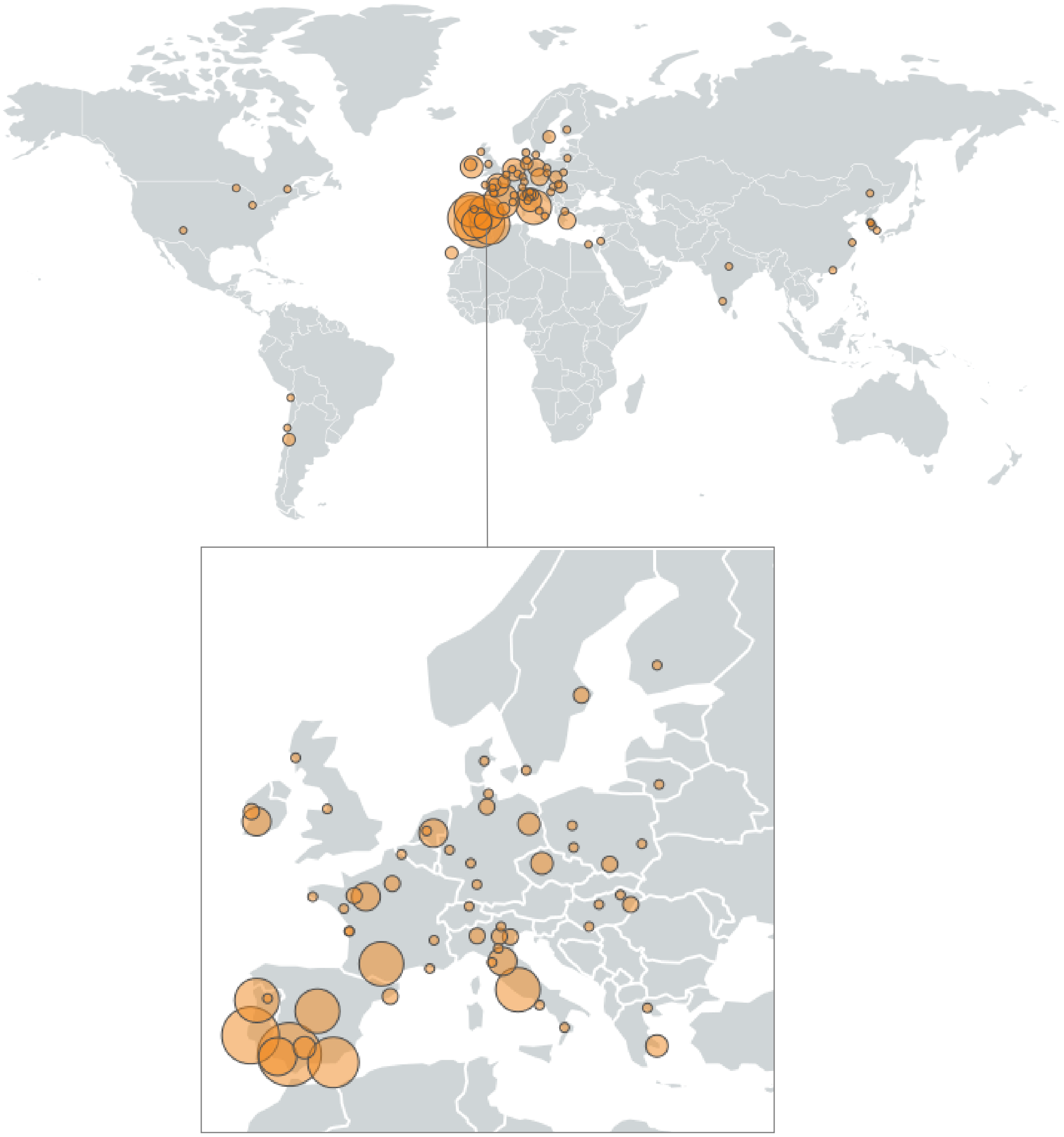

f.

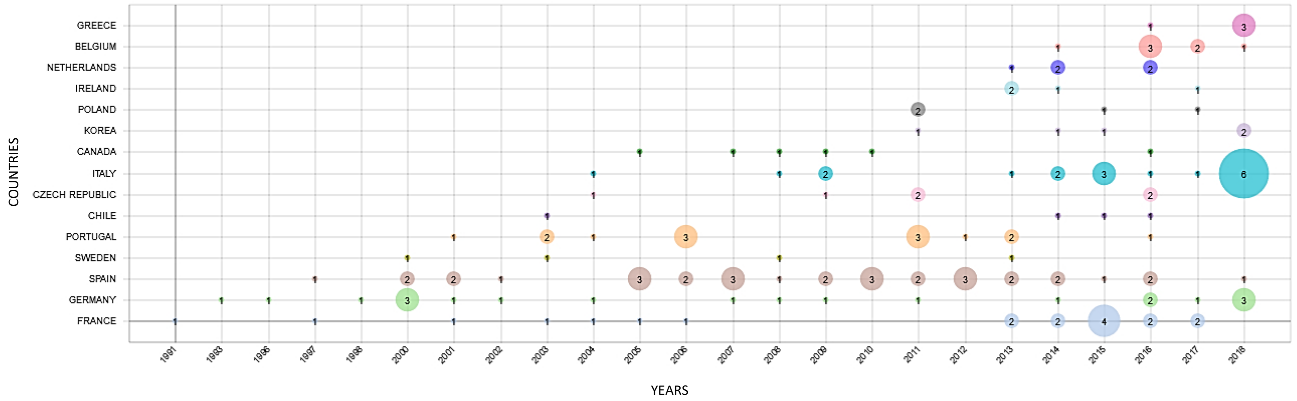

g.

Countries	Publications
SPAIN	33
GERMANY	20
FRANCE	19
ITALY	18
PORTUGAL	14
BELGIUM	7
CANADA	6
CZECH REPUBLIC	6
KOREA	5
NETHERLANDS	5
CHILE	4
GREECE	4
IRELAND	4
POLAND	4
SWEDEN	4

h.

Cities	Publications
Seville	9
Lisbon	8
Almeria	7
Madrid	6
Porto	6
Rome	6
Toulouse	6
Huelva	5
Florence	4
Le Mans	4
Limerick	4
Wageningen	4
Athens	3
Berlin	3
Cordoba	3

i.

Emerging concepts	GF
Diverse	4
Medicine	4
Bar	3
Microalgal specy	3
Nutraceutic	3
Almeriensis	2
Aquatic	2
Art	2
Bench	2
Bench scale	2
Bench scale reactor	2
Biofilm	2
Carbohydrate	2
Carotenoid extraction	2
Chloroform	2
Contaminant	2
Contamination	2
Crucial	2
Deal	2
Delivery	2

j.

Journals and number of publications	IF	Publications
JOURNAL OF APPLIED PHYCOLOGY	2,635	10
Algal Research-Biomass Biofuels and Bioproducts	3,723	10
APPLIED MICROBIOLOGY AND BIOTECHNOLOGY	3,67	5
Marine Drugs	3,772	5
AQUACULTURE NUTRITION	2,098	4
BIOTECHNOLOGY AND BIOENGINEERING	4,26	3
BIORESOURCE TECHNOLOGY	6,669	3
BIOTECHNOLOGY LETTERS	2,154	3
JOURNAL OF BIOTECHNOLOGY	3,163	3
AQUACULTURE	3,022	2
ANALYTICAL AND BIOANALYTICAL CHEMISTRY	3,286	2
EUROPEAN FOOD RESEARCH AND TECHNOLOGY	2,056	2
EUROPEAN JOURNAL OF PHYCOLOGY	2,526	2
FOOD CHEMISTRY	5,399	2
JOURNAL OF PHYCOLOGY	2,831	2

k.

Title of publications	Citations	Date
Outdoor cultivation of microalgae for carotenoid production: Current state and perspectives	355	2007
Microorganisms and microalgae as sources of pigments for food use: A scientific oddity or an industrial reality?	280	2005
Carotenoid content of chlorophycean microalgae: factors determining lutein accumulation in *Muriellopsis* sp. (Chlorophyta)	157	2000
Evaluation of different cell disruption processes on encysted cells of *Haematococcus pluvialis*: Effects on astaxanthin recovery and implications for bio-availability	153	2001
Microalgae as sources of high added-value compounds-a brief review of recent work	140	2011
Cell fragility - The key problem of microalgae mass production in closed photobioreactors	114	1991
Potential for green microalgae to produce hydrogen, pharmaceuticals and other high value products in a combined process	113	2013
Astaxanthin-rich algal meal and vitamin C inhibit *Helicobacter pylori* infection in BALB/cA mice	102	2000
Biotechnological production of astaxanthin with Phaffia rhodozyma/Xanthophyllomyces dendrorhous	101	2011
Optimization of culture medium for the continuous cultivation of the microalga *Haematococcus pluvialis*	98	2000
Comparison of microalgal biomass profiles as novel functional ingredient for food products	95	2013
Colouring ornamental fish (*Cyprinus carpio* and *Carassius auratus*) with microalgal biomass	87	2003
Aqueous extraction of proteins from microalgae: Effect of different cell disruption methods	84	2014
Subcritical water extraction and characterization of bioactive compounds from *Haematococcus pluvialis* microalga	84	2010
Analysis and enhancement of astaxanthin accumulation in *Haematococcus pluvialis*	79	2005

S11. Bibliometric overview of the research on *Haematococcus* sp. in 129 European scientific papers. Main concepts (a), concepts network (b), annual production (c), global collaborations (d), European collaborations (e), annual production by countries (f), main countries (g), main cities (h), emerging concepts (i), main journals (j) and main citations (k).

***Acutodesmus* sp.**

a.

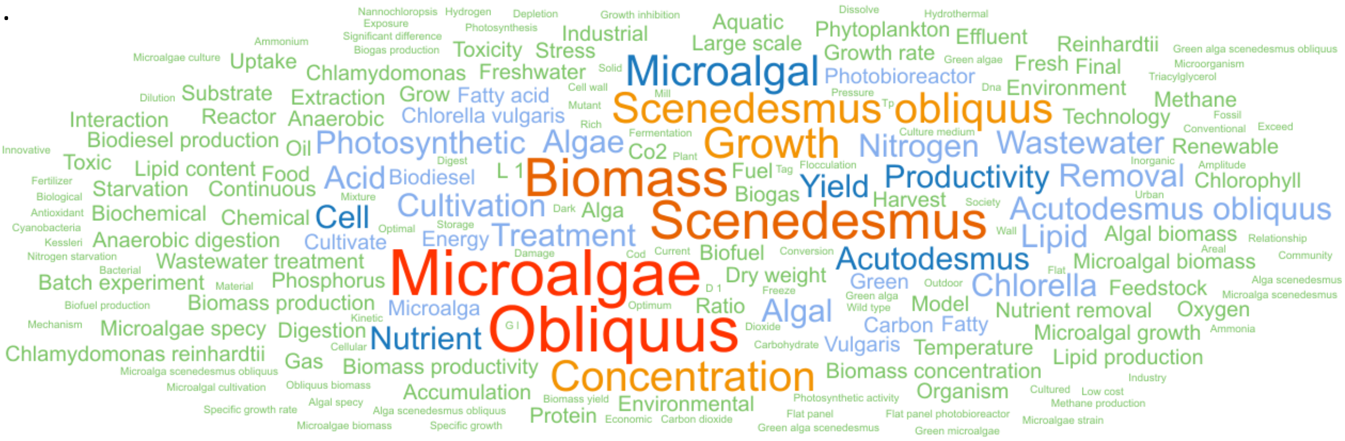

b.

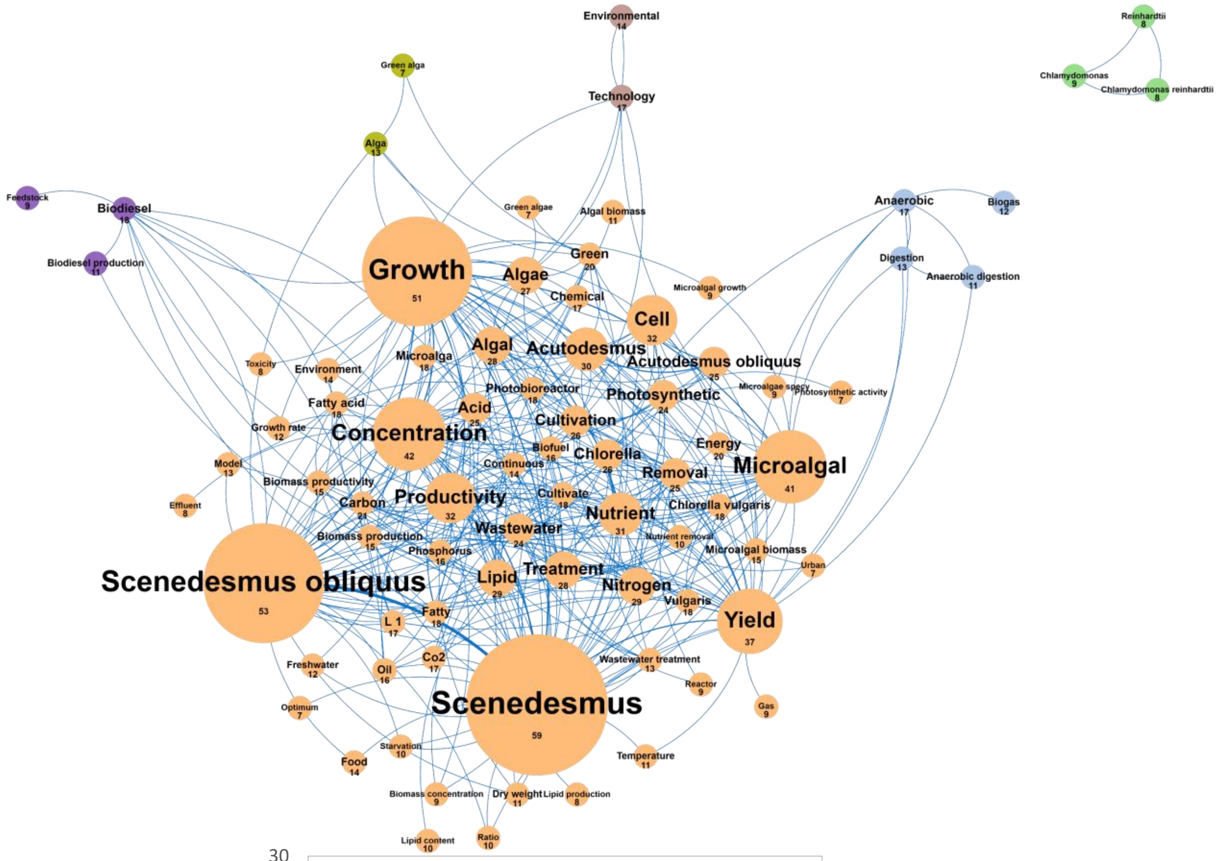

c.

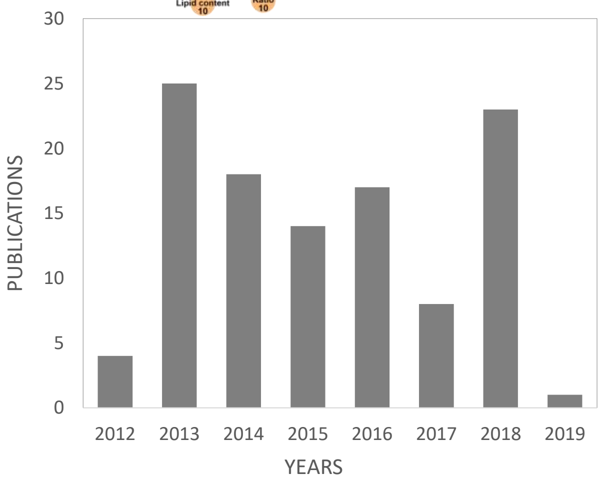

d.

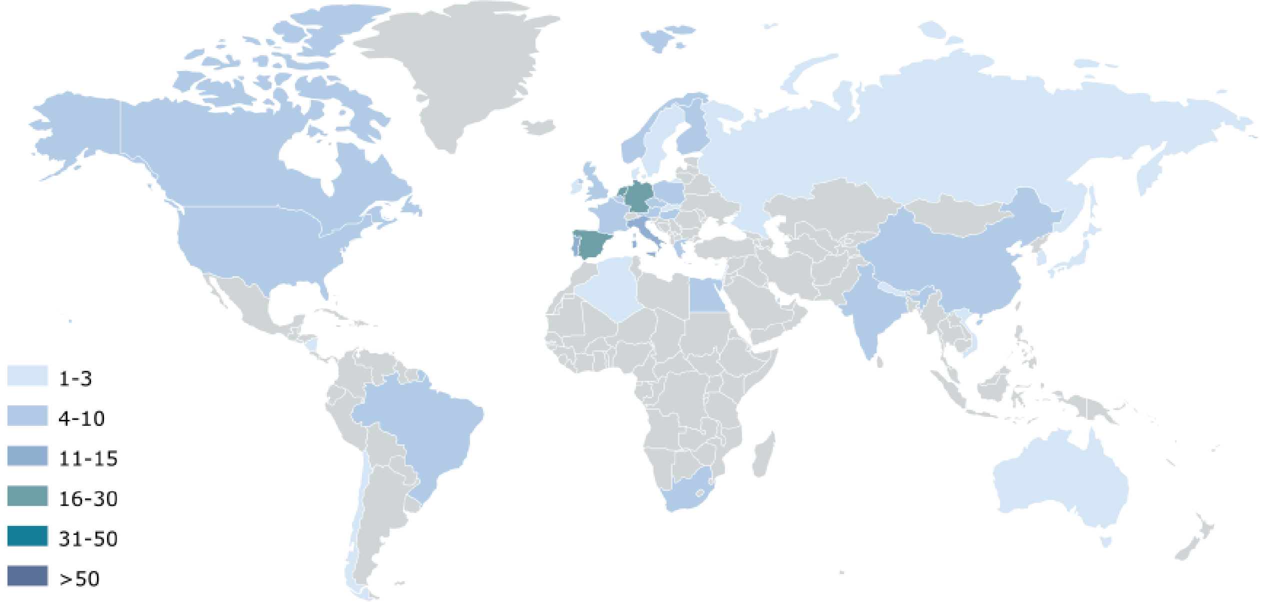

e.

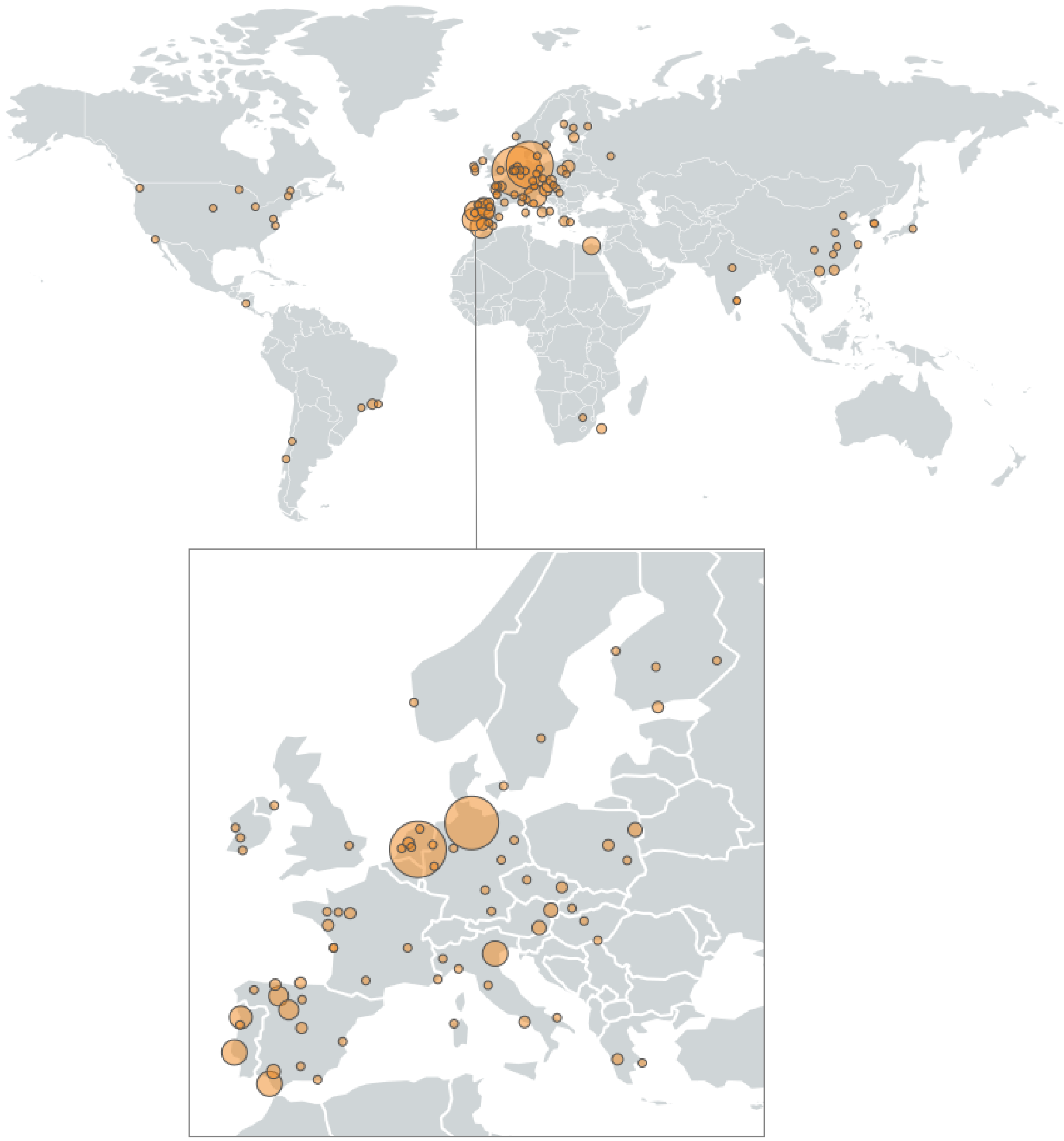

f.

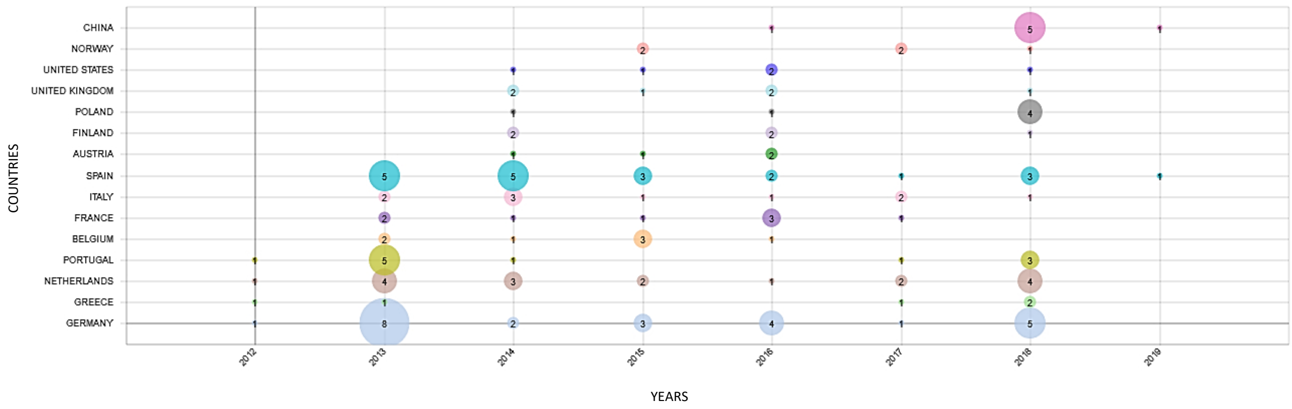

g.

Countries	Publications
GERMANY	24
SPAIN	20
NETHERLANDS	17
PORTUGAL	11
ITALY	10
FRANCE	8
BELGIUM	7
CHINA	7
POLAND	6
UNITED KINGDOM	6
FINLAND	5
GREECE	5
NORWAY	5
UNITED STATES	5
AUSTRIA	4

h.

Cities	Publications
Wageningen	15
Hamburg	14
Cadiz	6
Lisbon	6
Padova	6
Porto	5
Leon	4
Tanta	4
Valladolid	4
Bialystok	3
Graz	3
Seville	3
Vienna	3
Amsterdam	2
Guangzhou	2

i.

Emerging concepts	GF
Tp	5
Cod	4
Cell number	3
Continuous mode	3
Dynamic	3
Exogenous	3
Tn	3
Aeration	2
Antibacterial	2
Aquatic organism	2
Ascorbate	2
Ascorbate peroxidase	2
Auxin	2
Batch operation	2
Bioaccumulation	2
Bioenergy	2
Bioremediation	2
Breed	2
Carotene	2
Catalase	2

j.

Journals and number of publications	IF	Publications
BIORESOURCE TECHNOLOGY	6,669	26
WATER RESEARCH	7,913	7
BIOPROCESS AND BIOSYSTEMS ENGINEERING	2,371	5
Algal Research-Biomass Biofuels and Bioproducts	3,723	5
BIOMASS & BIOENERGY	3,537	4
JOURNAL OF APPLIED PHYCOLOGY	2,635	4
JOURNAL OF BIOTECHNOLOGY	3,163	4
Marine Drugs	3,772	3
PLoS One	2,776	3
AQUATIC TOXICOLOGY	3,794	2
APPLIED MICROBIOLOGY AND BIOTECHNOLOGY	3,67	2
ENVIRONMENTAL SCIENCE & TECHNOLOGY	7,149	2
FRESHWATER BIOLOGY	3,404	2
SCIENCE OF THE TOTAL ENVIRONMENT	5,589	2
SEPARATION AND PURIFICATION TECHNOLOGY	5,107	2

k.

Title of publications	Citations	Date
The impact of nitrogen starvation on the dynamics of triacylglycerol accumulation in nine microalgae strains	259	2012
Nanoplastic affects growth of *S. obliquus* and reproduction of *D. magna*	148	2014
Effect of light intensity, pH, and temperature on triacylglycerol (TAG) accumulation induced by nitrogen starvation in *Scenedesmus obliquus*	114	2013
Anaerobic digestate as substrate for microalgae culture: The role of ammonium concentration on the microalgae productivity	79	2014
Nitrogen availability influences phosphorus removal in microalgae-based wastewater treatment	79	2015
Capability of different microalgae species for phytoremediation processes: Wastewater tertiary treatment, CO2 bio-fixation and low cost biofuels production	71	2014
Performance of a flat panel reactor in the continuous culture of microalgae in urban wastewater: Prediction from a batch experiment	71	2013
Growth of three microalgae strains and nutrient removal from an agro-zootechnical digestate	65	2013
Impacts of microalgae pre-treatments for improved anaerobic digestion: Thermal treatment, thermal hydrolysis, ultrasound and enzymatic hydrolysis	64	2014
Microalgal Cultivation in Treating Liquid Digestate from Biogas Systems	63	2016
Influence of strain-specific parameters on hydrothermal liquefaction of microalgae	57	2013
Highly valuable microalgae: Biochemical and topological aspects	52	2013
Superior triacylglycerol (TAG) accumulation in starchless mutants of *Scenedesmus obliquus*: (I) mutant generation and characterization	50	2014
Optimization of biomass and fatty acid productivity of *Scenedesmus obliquus* as a promising microalga for biodiesel production	50	2013
Superior triacylglycerol (TAG) accumulation in starchless mutants of *Scenedesmus obliquus*: (II) evaluation of TAG yield and productivity in controlled photobioreactors	49	2014

S12. Bibliometric overview of the research on *Acutodesmus* sp. in 110 European scientific papers. Main concepts (a), concepts network (b), annual production (c), global collaborations (d), European collaborations (e), annual production by countries (f), main countries (g), main cities (h), emerging concepts (i), main journals (j) and main citations (k).

***Synechocystis* sp.**

a.

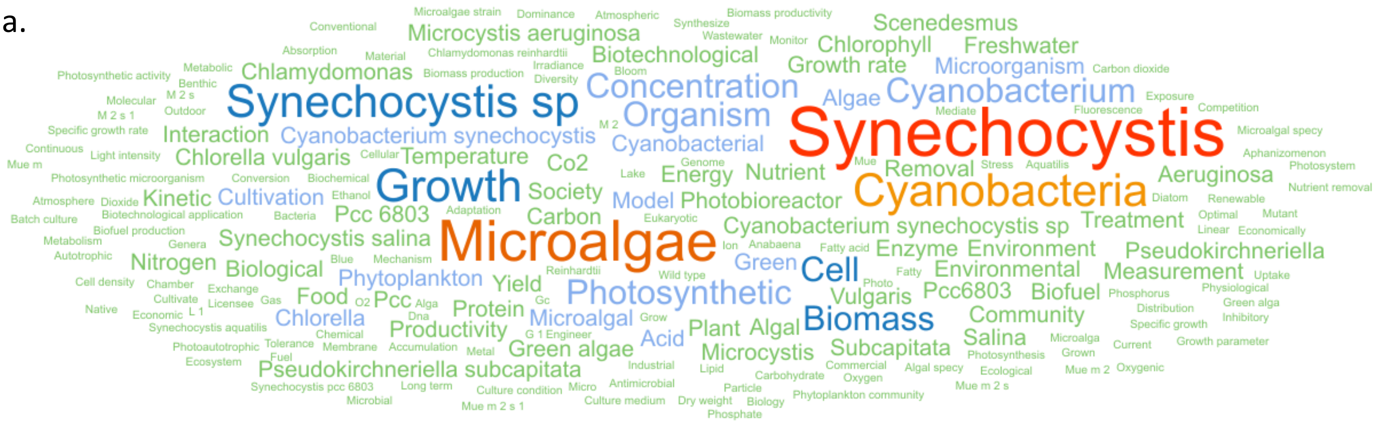

b.

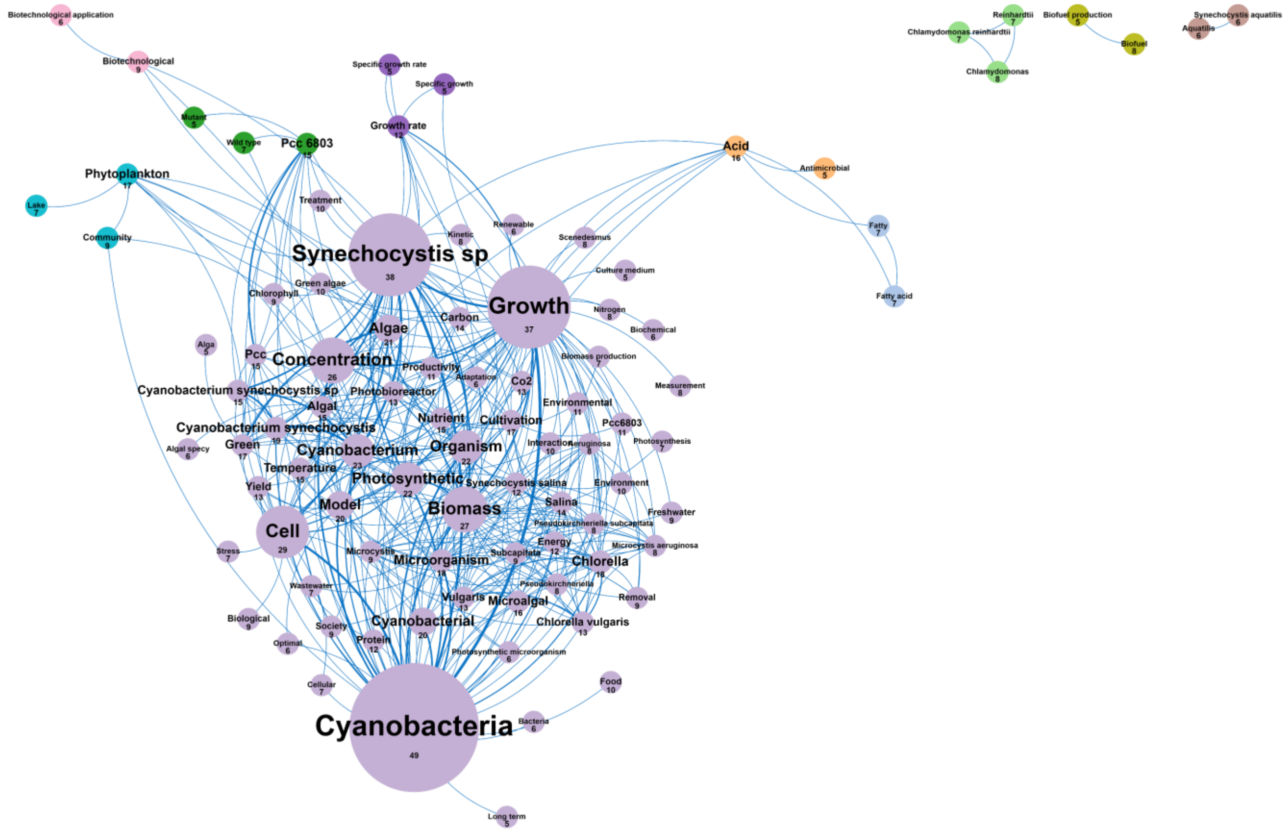

c.

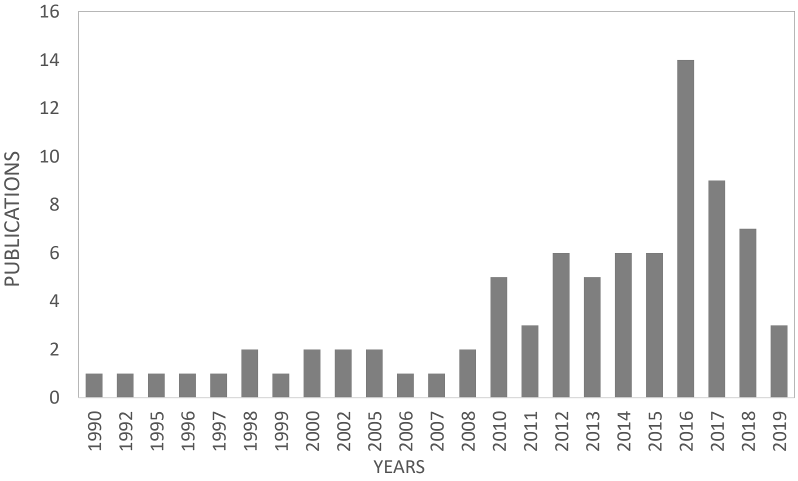

d.

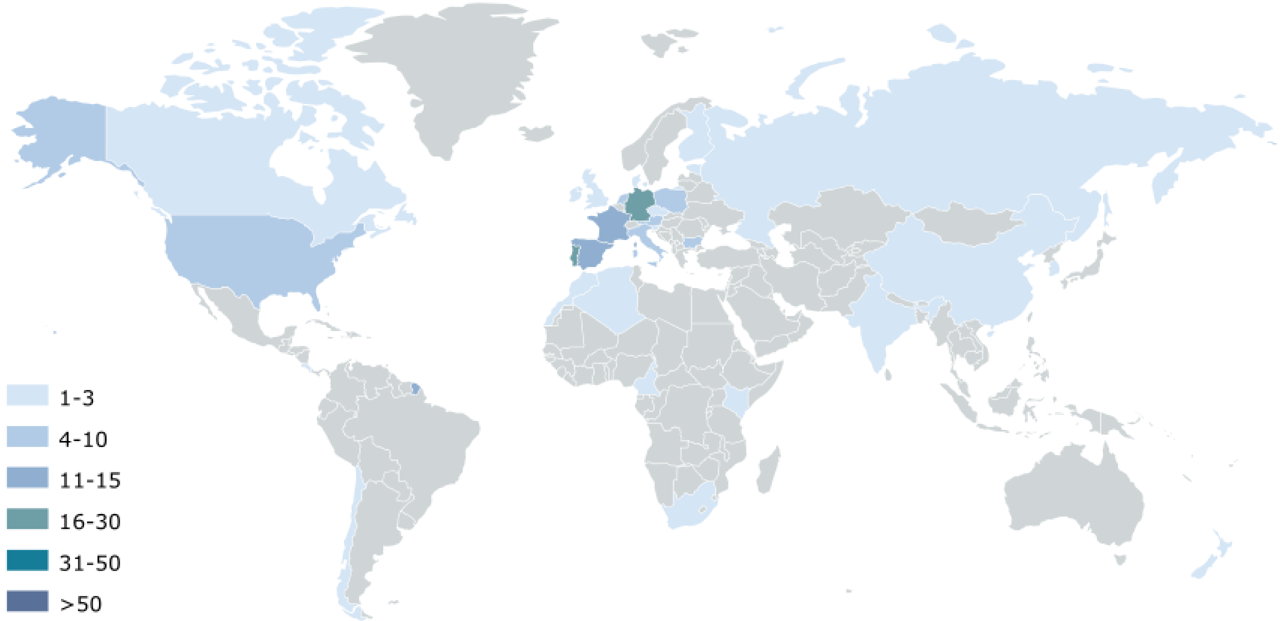

e.

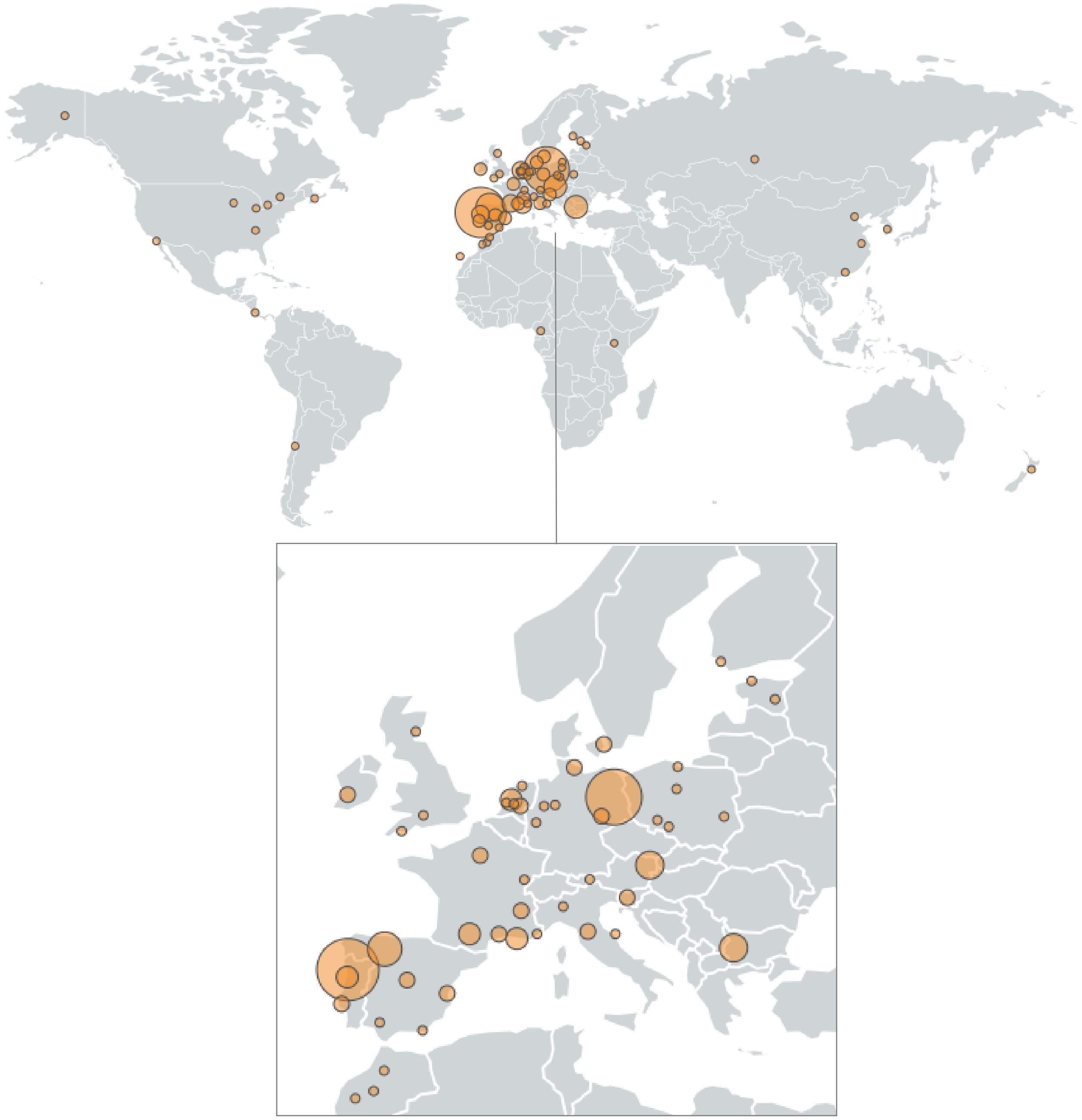

f.

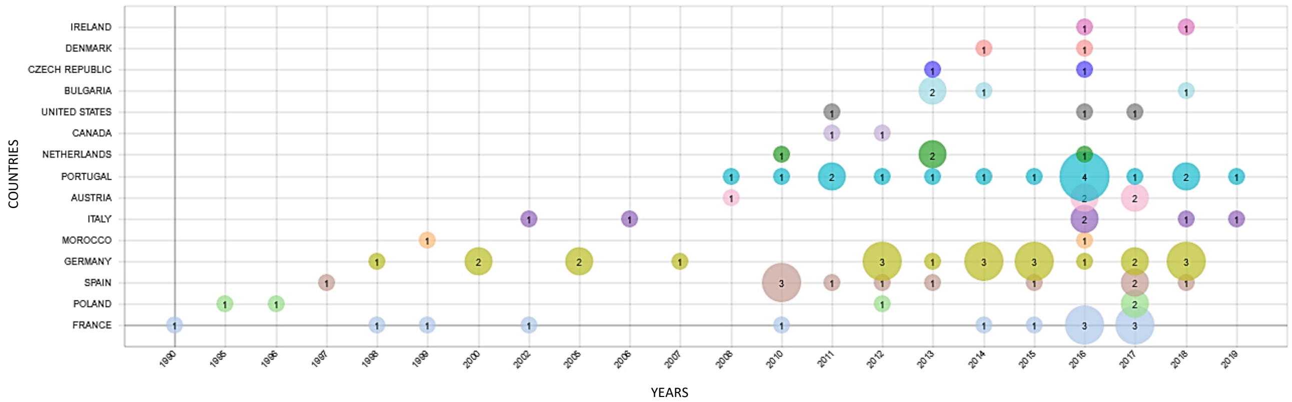

g.

Countries	Publications
GERMANY	22
PORTUGAL	16
FRANCE	13
SPAIN	11
ITALY	6
AUSTRIA	5
POLAND	5
BULGARIA	4
NETHERLANDS	4
UNITED STATES	3
CANADA	2
CZECH REPUBLIC	2
DENMARK	2
IRELAND	2
MOROCCO	2

h.

Cities	Publications
Porto	10
Berlin	9
Leon	5
Sofia	4
Vienna	4
Amsterdam	3
Aveiro	3
Marseille	3
Toulouse	3
Copenhagen	2
Florence	2
Grenoble	2
Kiel	2
Leipzig	2
Limerick	2

i.

Emerging concepts	GF
Springer nature	3
Highest value	2
Pharmaceutical	2
Production process	2
Springer nature b	2
Synthase	2
Technological	2
Acclimation	1
Acclimation process	1
Acid phosphatase	1
Acid phosphatase activity	1
Acting	1
Adaptation process	1
Adhere	1
Adhesion	1
Adhesion assay	1
Adsorbent	1
Adsorbent material	1
Adsorption	1
Adsorptive	1

j.

Journals and number of publications	IF	Publications
JOURNAL OF APPLIED PHYCOLOGY	2,635	5
Algal Research-Biomass Biofuels and Bioproducts	3,723	5
BIORESOURCE TECHNOLOGY	6,669	3
APPLIED MICROBIOLOGY AND BIOTECHNOLOGY	3,67	2
ENERGY CONVERSION AND MANAGEMENT	7,181	2
HYDROBIOLOGIA	2,325	2
INTERNATIONAL JOURNAL OF HYDROGEN ENERGY	4,084	2
JOURNAL OF BIOTECHNOLOGY	3,163	2
Marine Drugs	3,772	2
RSC Advances	3,049	2
Water	2,524	2
ENVIRONMENTAL MICROBIOLOGY	5,147	1
CURRENT OPINION IN BIOTECHNOLOGY	8,083	1
CHEMOSPHERE	5,108	1
BMC MICROBIOLOGY	3,287	1

k.

Title of publications	Citations	Date
Protein measurements of microalgal and cyanobacterial biomass	233	2010
Screening for bioactive compounds from algae	183	2010
Potential of industrial biotechnology with cyanobacteria and eukaryotic microalgae	177	2013
Determination of DPPH radical oxidation caused by methanolic extracts of some microalgal species by linear regression analysis of spectrophotometric measurements	103	2007
Auxiliary electron transport pathways in chloroplasts of microalgae	99	2010
Growth rate of four freshwater algae in relation to light and temperature	80	1990
Limiting steps of hydrogen production in *Chlamydomonas reinhardtii* and *Synechocystis* PCC 6803 as analysed by light-induced gas exchange transients	76	2002
Parameterization of photosystem II photoinactivation and repair	70	2012
The effect of light supply on microalgal growth, CO2 uptake and nutrient removal from wastewater	42	2014
Phytoplankton community relationship to environmental variables in three Kenyan Rift Valley saline-alkaline lakes	41	2008
Elevated CO2 concentrations affect the elemental stoichiometry and species composition of an experimental phytoplankton community	40	2013
Axenic cultivation of anoxygenic phototrophic bacteria, cyanobacteria, and microalgae in a new closed tubular glass photobioreactor	38	2000
Retene in pyrolysates of algal and bacterial organic matter	36	2000
Transcriptomic response to prolonged ethanol production in the cyanobacterium *Synechocystis* sp. PCC6803	35	2014
Antibacterial and antifungal activities of selected microalgae and cyanobacteria	33	2013

S13. Bibliometric overview of the research on *Synechocystis* sp. in 82 European scientific papers. Main concepts (a), concepts network (b), annual production (c), global collaborations (d), European collaborations (e), annual production by countries (f), main countries (g), main cities (h), emerging concepts (i), main journals (j) and main citations (k).

***Schizochytrium* sp.**

a.

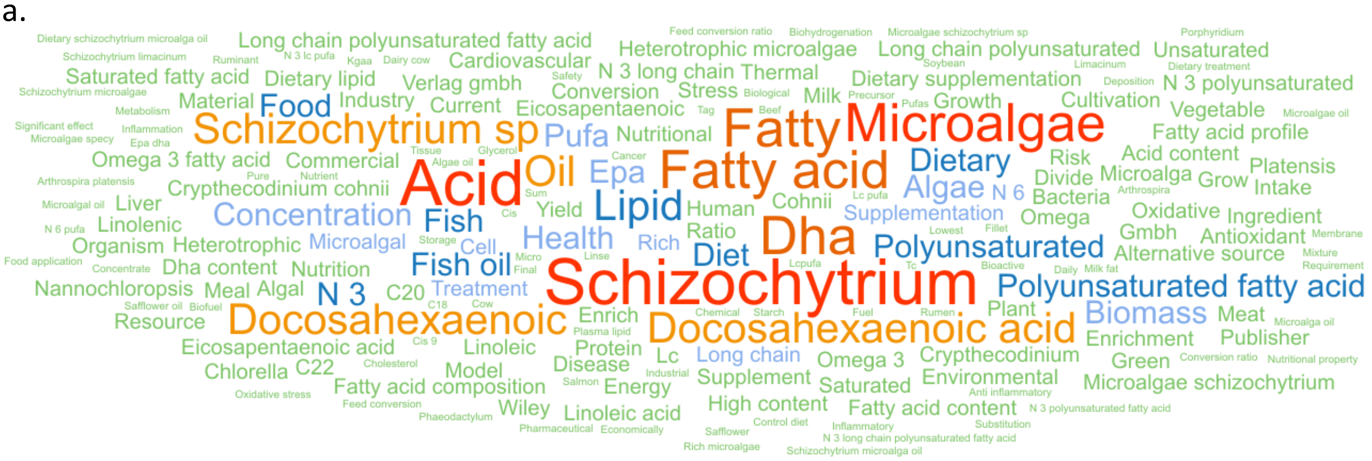

b.

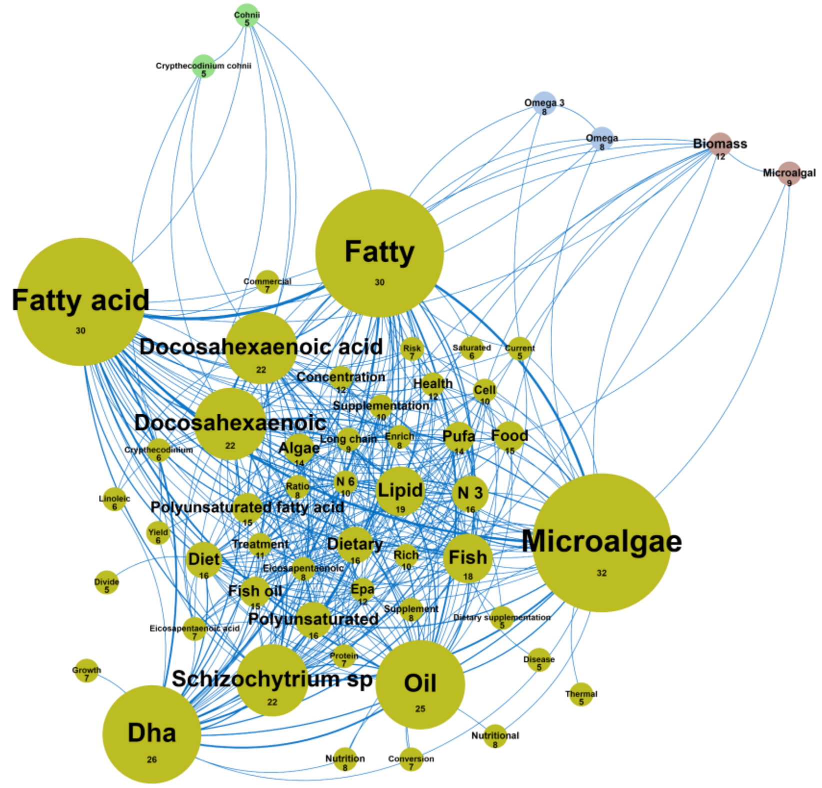

c.

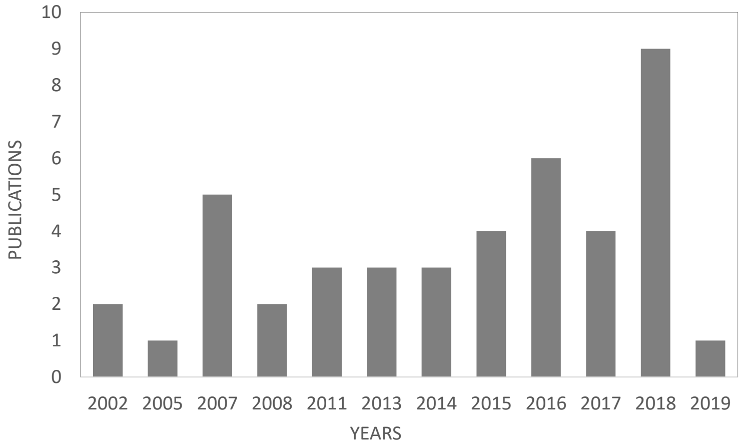

d.

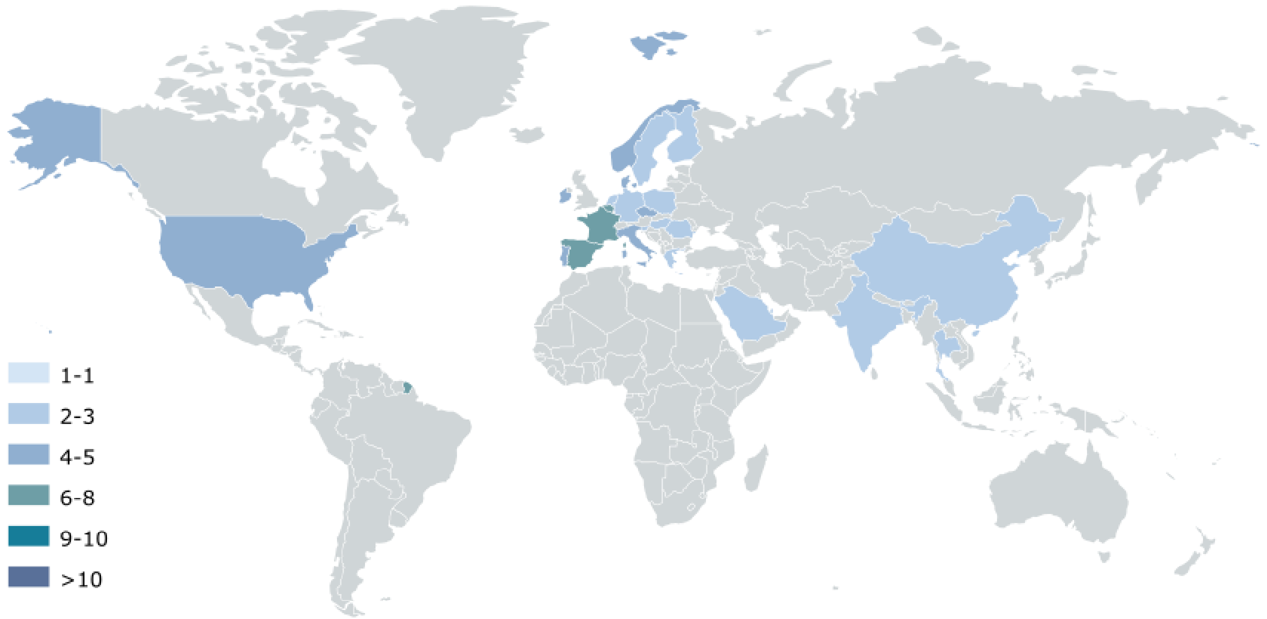

e.

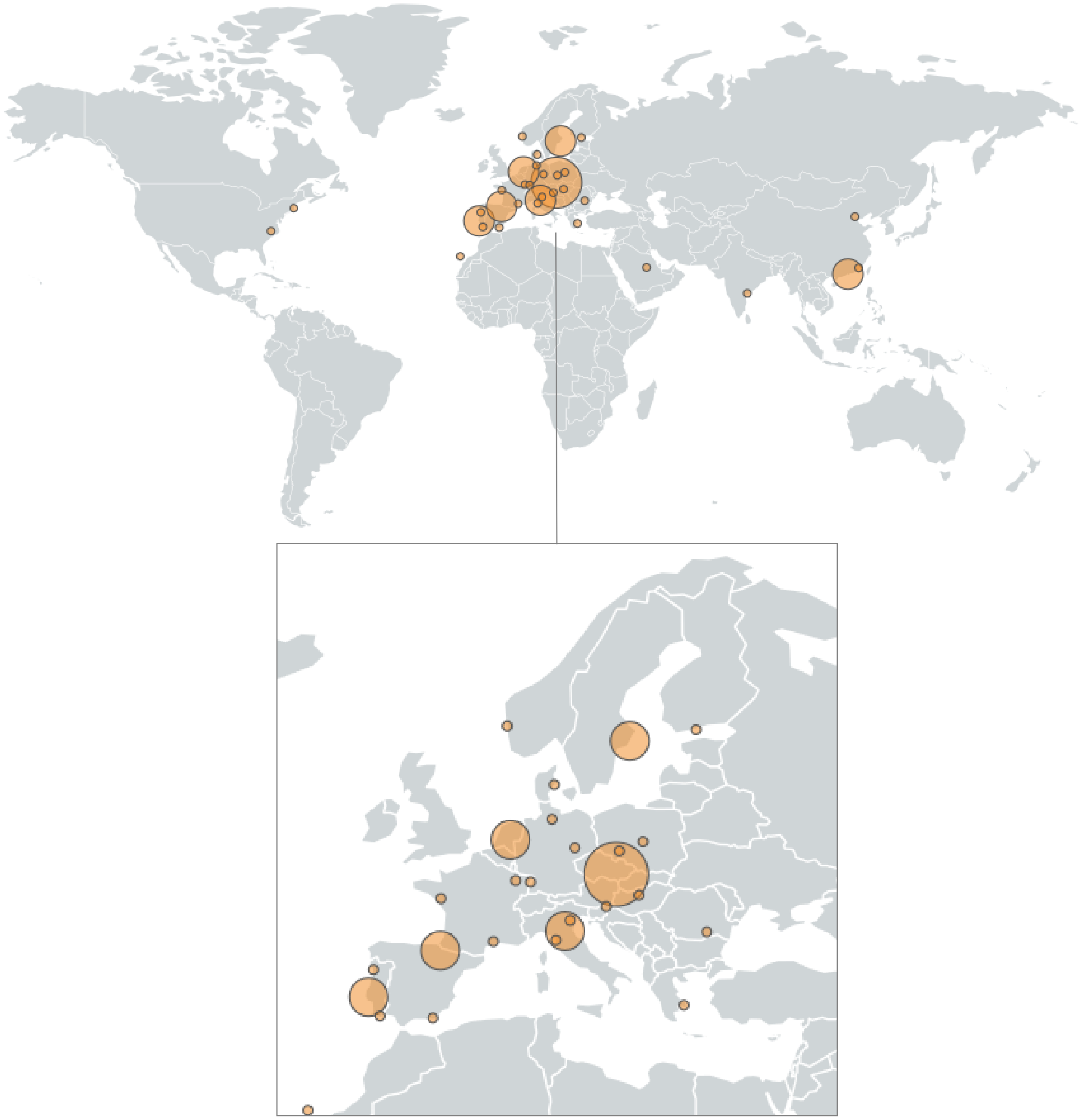

f.

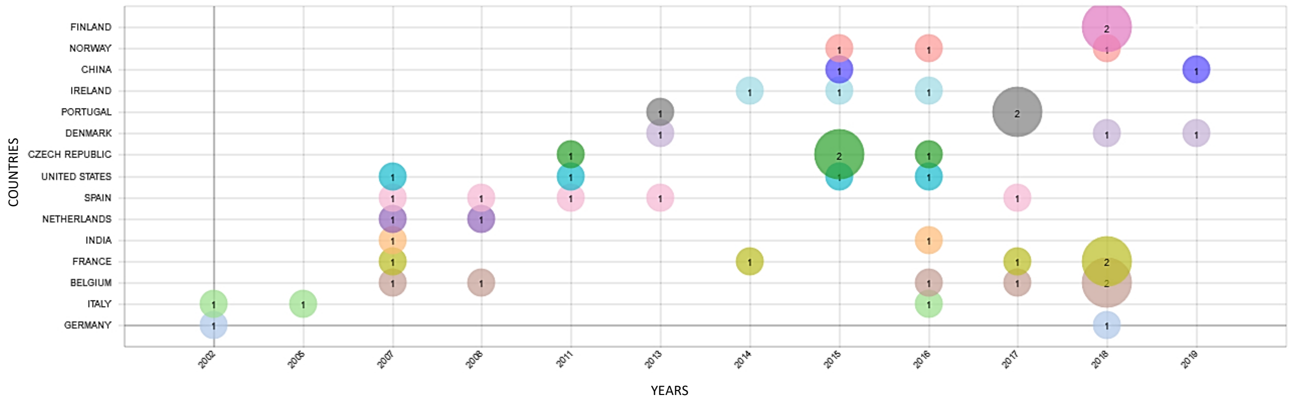

g.

Countries	Publications
BELGIUM	6
FRANCE	5
SPAIN	5
CZECH REPUBLIC	4
UNITED STATES	4
DENMARK	3
IRELAND	3
ITALY	3
NORWAY	3
PORTUGAL	3
CHINA	2
FINLAND	2
GERMANY	2
INDIA	2
NETHERLANDS	2

h.

Cities	Publications
Brno	3
Bologna	2
Lisbon	2
Pampelune	2
Shenzhen	2
Stockholm	2
Wageningen	2
Almeria	1
Arhus	1
Athens	1
Beijing	1
Bergen	1
Boston	1
Bucharest	1
Budapest	1

i.

Emerging concepts	GF
Bioactive	2
Chemical	2
Concentrate	2
Consume	2
Enzymatic	2
Food application	2
Glycerol	2
Limacinum	2
Microalgal oil	2
N 6 pufa	2
Nmr	2
Phaeodactylum	2
Porphyridium	2
Pure	2
Rapeseed	2
Rapeseed oil	2
Regardless	2
Rm	2
Ruminant	2
Schizochytrium limacinum	2

j.

Journals and number of publications	IF	Publications
EUROPEAN JOURNAL OF LIPID SCIENCE AND TECHNOLOGY	1,852	4
Algal Research-Biomass Biofuels and Bioproducts	3,723	3
AQUACULTURE	3,022	2
Livestock Science	1,376	2
FOOD CHEMISTRY	5,399	2
JOURNAL OF ANIMAL PHYSIOLOGY AND ANIMAL NUTRITION	1,703	2
Progress in Nutrition	0,265	2
Microbial Cell Factories	4,402	1
MEAT SCIENCE	3,483	1
Marine Drugs	3,772	1
FOOD AND CHEMICAL TOXICOLOGY	3,775	1
JOURNAL OF SUPERCRITICAL FLUIDS	3,481	1
JOURNAL OF POLYMERS AND THE ENVIRONMENT	2,765	1
JOURNAL OF DAIRY SCIENCE	3,082	1
ENERGY CONVERSION AND MANAGEMENT	7,181	1

k.

Title of publications	Citations	Date
Omega-3 fatty acids for nutrition and medicine: Considering microalgae oil as a vegetarian source of EPA and DHA	85	2007
Alternative sources of n-3 long-chain polyunsaturated fatty acids in marine microalgae	82	2013
Effect of dietary starch or micro algae supplementation on rumen fermentation and milk fatty acid composition of dairy cows	80	2008
Crypthecodinium cohnii and *Schizochytrium* sp. as potential substitutes to fisheries-derived oils from seabream (*Sparus aurata*) microdiets	70	2008
Development of dry fermented sausages rich in docosahexaenoic acid with oil from the microalgae *Schizochytrium* sp.: Influence on nutritional properties, sensorial quality and oxidation stability	53	2007
Changes in rumen biohydrogenation intermediates and ciliate protozoa diversity after algae supplementation to dairy cattle	44	2007
Biological potential of microalgae in China for biorefinery-based production of biofuels and high value compounds	28	2015
A comparative study on the effect of algal and fish oil on viability and cell proliferation of Caco-2 cells	26	2007
Metabolism, health and fillet nutritional quality in Atlantic salmon (*Salmo salar*) fed diets containing n-3-rich microalgae	26	2015
Microalgae and organic minerals enhance lipid retention efficiency and fillet quality in Atlantic salmon (*Salmo salar* L.)	23	2016
Seaweed lipids as nutraceuticals	23	2011
Supercritical fluid extraction from microalgae with high content of LC-PUFAs. A case of study: Sc-CO2 oil extraction from *Schizochytrium* sp	16	2016
Microalgae as feed ingredients for livestock production and meat quality: A review	15	2017
Manipulation of fatty acid and antioxidant profiles of the microalgae *Schizochytrium* sp. through flaxseed oil supplementation	10	2014
Docosahexaenoic acid in the treatment of rheumatoid arthritis: A double-blind, placebo-controlled, randomized cross-over study with microalgae vs. sunflower oil	8	2018

S14. Bibliometric overview of the research on *Schizochytrium* sp. in 43 European scientific papers. Main concepts (a), concepts network (b), annual production (c), global collaborations (d), European collaborations (e), annual production by countries (f), main countries (g), main cities (h), emerging concepts (i), main journals (j) and main citations (k).
